# COVID-19 non-pharmaceutical intervention portfolio effectiveness and risk communication predominance

**DOI:** 10.1038/s41598-021-88309-1

**Published:** 2021-05-19

**Authors:** Louis Yat Hin Chan, Baoyin Yuan, Matteo Convertino

**Affiliations:** 1 Graduate School of Medicine, Hokkaido University, Sapporo, Japan; 2 Nexus Group, Graduate School of Information Science and Technology, Hokkaido University, Sapporo, Japan; 3 Department of Infectious Disease Epidemiology and Modelling, Norwegian Institute of Public Health, Oslo, Norway; 4 School of Mathematics, South China University of Technology, Guangzhou, China; 5 Institute of Environment and Ecology, Tsinghua Shenzhen International Graduate School, Tsinghua University, Shenzhen, China

**Keywords:** Viral infection, Applied mathematics, Statistics

## Abstract

Non-pharmaceutical interventions (NPIs) including resource allocation, risk communication, social distancing and travel restriction, are mainstream actions to control the spreading of Coronavirus disease 2019 (COVID-19) worldwide. Different countries implemented their own combinations of NPIs to prevent local epidemics and healthcare system overloaded. Portfolios, as temporal sets of NPIs have various systemic impacts on preventing cases in populations. Here, we developed a probabilistic modeling framework to evaluate the effectiveness of NPI portfolios at the macroscale. We employed a deconvolution method to back-calculate incidence of infections and estimate the effective reproduction number by using the package EpiEstim. We then evaluated the effectiveness of NPIs using ratios of the reproduction numbers and considered them individually and as a portfolio systemically. Based on estimates from Japan, we estimated time delays of symptomatic-to-confirmation and infection-to-confirmation as 7.4 and 11.4 days, respectively. These were used to correct surveillance data of other countries. Considering 50 countries, risk communication and returning to normal life were the most and least effective yielding the aggregated effectiveness of 0.11 and − 0.05 that correspond to a 22.4% and 12.2% reduction and increase in case growth. The latter is quantified by the change in reproduction number before and after intervention implementation. Countries with the optimal NPI portfolio are along an empirical Pareto frontier where mean and variance of effectiveness are maximized and minimized independently of incidence levels. Results indicate that implemented interventions, regardless of NPI portfolios, had distinct incidence reductions and a clear timing effect on infection dynamics measured by sequences of reproduction numbers. Overall, the successful suppression of the epidemic cannot work without the non-linear effect of NPI portfolios whose effectiveness optimality may relate to country-specific socio-environmental factors.

## Introduction

### Pandemic and interventions

The ongoing COVID-19 pandemic is determining an unprecedented systemic impact on socio-economic activities worldwide as well as positive environmental outcomes^1,2^. COVID-19 is certainly the most serious public health crisis after the 1918 flu pandemic, but in many regards the worst crisis of humanity considering the systemicity of the problem in relation to the globalization of humanity^3^. For this very precise reason and the unparalleled availability of data, this pandemic also offers the opportunity to analyze and reflect, as never before, about all elements of risks—vulnerability, exposure, and hazard—in an holistic way. The pandemic initially emerged in Wuhan, China in December 2019^4^. As of the 14th June 2020, more than 7.6 million confirmed cases and 0.4 million deaths from 215 countries or regions all over the world have been reported according to the World Health Organization (WHO)^5^. Currently, the virus continues to spread globally, independently of country development status, or rather affecting more developed countries than low- and middle-income countries due to their globalization enhancing transmission. Europe experienced a downward trend^6^ after the second wave but third waves are emerging in many places, including Europe and Asia, where the epidemic was thought to be contained. Thus, we cannot expect the pandemic to end anytime soon and comprehensive public health efforts are necessary to control the pandemic in the future. In relation to this, data- and decision-driven efforts that look into the effectiveness of multiple joined interventions in a portfolio perspective^7–10^ are necessary rather than just producing case forecasts as many efforts are ongoing^11,12^. Some evaluation tools have been proposed recently, such as the Global COVID Index^13^ and the Government Response Stringency Index^14^, but these tools focus on ranking countries or measuring the amount of interventions rather than dissecting and quantifying vulnerabilities and interventions precisely. Additionally, these indices rely on information of the pandemic as it is without embracing a probabilistic approach and questioning wether systematic uncertainties to correct are present. With these issues in mind the fundamental question is whether one predominant intervention, connected to all feasible others, exists in abating the risk.

### Intervention portfolio and uncertainty

In response to the rapid spread of COVID-19 governments around the world have implemented multiple non-pharmaceutical interventions (NPIs) to mitigate or suppress COVID-19 infections^15–19^. Pharmaceutical interventions such vaccines started to be available in January 2021 so NPIs only affected incidence rate in previous periods from COVID-19 onset with different degrees of individual restriction. According to various social and political conditions, each country has taken different schemes of NPIs involving different intervention options considering type, timing, duration and hypothesized strength^20,21^. These interventions have been driven by a reaction approach in which most countries were reacting to increasing cases by implementing what other countries implemented to restrict individual behavior leading to infection. Certainly this approach is suboptimal because it does not consider (i) the underlying socio-environmental and economic factors of a country, which can also encompass measures to reduce exposure; and (ii) the portfolio effect related to all interventions’ interdependencies, with the inclusion of this shared effect information^7–9^. A balanced intervention strategy to significantly reduce morbidity and mortality due to infections, but also to minimize the impact on daily life of people, is considered an optimal intervention portfolio. A portfolio where environmental health and risk information quality (or misinformation, vice versa) are also considered as primary non coercive interventions at the population scale. Thus, the systemic evaluation of the effectiveness of interventions is valuable for public health authorities to develop preparedness plans timely, evaluate interventions over time, and design policies to decrease population vulnerabilities in long term. This evaluation should be based on reliable information which has also practical implications for risk communication a posteriori.

### Incidence and information

Epidemic curves of incidence provide the most valuable and intuitive information (in terms of estimates and visualization) of the temporal trend of epidemics. Generally, the epidemic curve available from public health authorities are based on dates of illness onset or reported cases. These dates are defined by the moment in which surveillance systems report cases but these dates may come late and do not take into account the time delay related to symptom manifestation and official reporting. Thus, the reconstruction of unobserved epidemic curves by dates of true infection would be immediately useful to estimate the effective reproduction numbers. In order to reconstruct epidemic curves we employ a deconvolution method that reconstructs the expected signal of infections before a cause (biological and systematic delays) produces modifications on that signal. Some deconvolution methods were proposed in literature to back-calculate infection curves, for example for HIV infections from AIDS time series^22^ and for influenza infections from death curves^23^. The Richardson-Lucy (RL) deconvolution was used in some recent studies to evaluate transmission of the COVID-19 pandemic^24–26^ and this is the model we use here to reconstruct case time series from imperfect surveillance data. The RL model was slightly modified for a better treatment of noise and the easy incorporation of available non-linear a priori information^27^.

### Proposed intervention effectiveness

The aim of this study is to develop a probabilistic pattern-oriented framework to quantitatively analyze the instantaneous and systemic impact of interventions by evaluating their effectiveness considering portfolios of implemented interventions. We emphasize how our approach is a complex system approach like Haug et al.^10^ where with a non-mechanistic model and limited assumptions we search for emerging patterns in data, in this case of effectiveness. This has implications for extracting epidemiological and intervention information useful for epidemic evaluation and control decision making. The analysis is performed by using the incidence curves of COVID-19 for 50 countries. The procedure to evaluate the effectiveness of interventions from incidence curves of confirmed cases consists of three steps:

- First, misreported curves of confirmed cases are corrected by removing negative incidence. After, incidence curves of infections were back-calculated from the curves of confirmation by using the RL deconvolution method^23^. The reporting delay was assumed as the convolution sum of the incubation period (with estimated mean and standard deviation of 5.5 and 2.3 days^28^) and the symptomatic-to-confirmation reporting delay that was estimated using maximum likelihood method (MLE);
- Second, the non-overlapping instantaneous reproduction numbers were estimated by using the EpiEstim package^29,30^. The generation time, defined as the time interval between primary and secondary cases, was assumed to be identical to the serial interval, defined as the time interval of illness onset, following a gamma distribution yielding mean 4.5 days and SD 4.5 days^31^. The non-overlapping reproduction numbers were assumed to be constant between implementations; and,
- Third, the effectiveness of portfolios, defined as the ratio of the reproduction numbers for each time in which one or multiple interventions were implemented, was evaluated. Then single intervention and aggregated portfolio effectiveness was quantified for each country.

The proposed portfolio model of intervention effectiveness is enormously useful because it allows one to (i) rectify data and evaluate surveillance systems; (ii) quantify the best intervention in term of risk abatement and in consideration of portfolio interdependencies, which can be useful to evaluate countries holistically; and (iii) tailor interventions during epidemics and design public health policy where population specific environment and communication, which contribute to systemic vulnerability, are taken into account.

## Results

### Time delay from infection to confirmation

**Figure 1:**
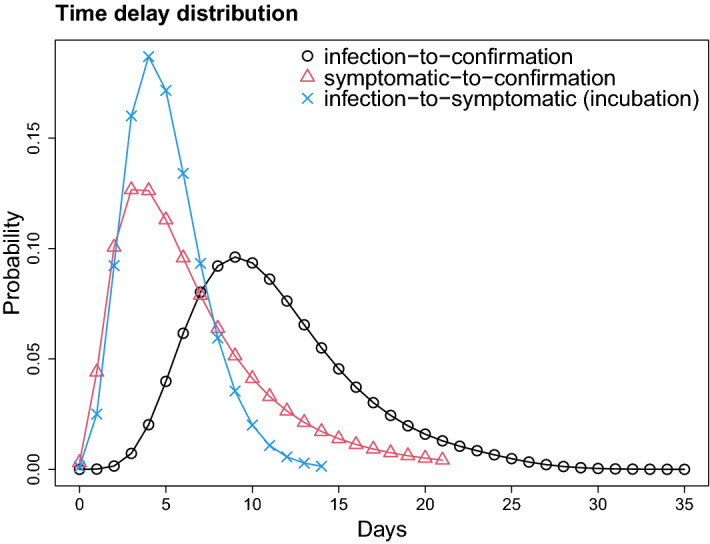
Distribution of time delays. The distribution of infection-to-confirmation $f^{ec}(t)$ (black) is a convolution of log-normal and gamma distributions, symptomatic-to-confirmation $f^{sc}(t)$ (red) and incubation period $f^{se}(t)$ (blue). The log-mean and log-SD of symptomatic-to-confirmation distribution are 1.79 and 0.65, estimated using the data from Japan. The shape and scale parameters of incubation period distribution are 5.807 and 0.948 respectively^28^. The mean values of infection-to-confirmation, symptomatic-to-confirmation and incubation period were 11.4, 7.4, and 5.5. Standard deviations were 4.7, 5.4 and 2.3, respectively.

The first step was to remove misreported incidence that is the reported negative incidence; then the % of misreporting was the proportion of this negative incidence. The overall misreporting percentage is low, i.e. 0.4%, and that corresponds to 24 out of 5405 cases that were misreported (Table S1). The symptomatic-to-confirmation period (i.e., confirmation or reporting period) following a log-normal distribution is more appropriate in consideration of the many factors affecting reporting as well as the event that reporting may not even occur. Thus, a distribution with a fatter tail is more appropriate to capture all these elements. Conversely, the incubation period distribution is much more “deterministic” and related to the biology of *Coronavirus* that is invariant across geographies (at least for the period we considered, i.e. the first wave). Thus, the symptomatic-to-confirmation time was fitted by a log-normal distribution using 8917 individuals from Japan^32^, yielding the estimates of the mean and standard deviation were 7.4 (95%CI 7.2–7.5) and 5.4 (95%CI 5.2–5.6) days. The time delay distribution of infection-to-confirmation is a convolution of gamma and log-normal distributions (incubation period and symptomatic-to-confirmation, respectively). The mean and standard deviation values were 11.4 and 4.7 days, respectively. All three distributions are shown in Fig. 1.

### Japan intervention effectiveness

**Figure 2:**
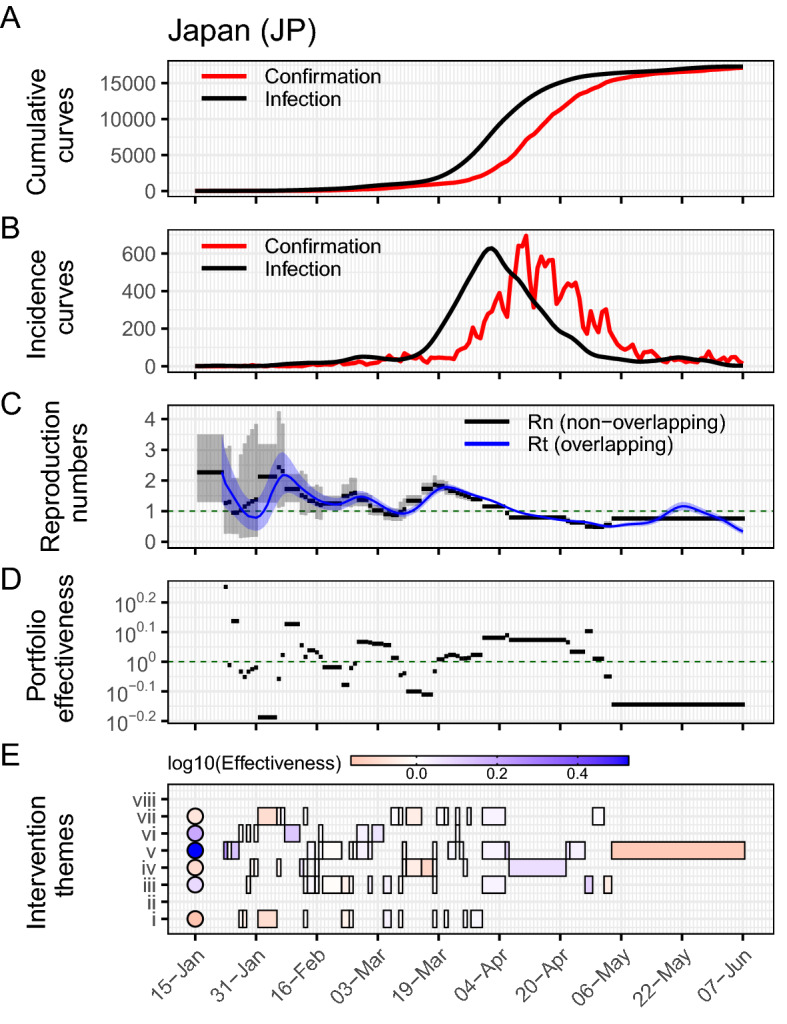
Reconstructed epidemic curves and evaluation of interventions implemented in Japan. (**A**) The cumulative curves of confirmation (red) and infection (black). (**B**) The incidence curves of confirmation (red) and infection (black). (**C**) The estimated instantaneous reproduction number, using non-overlapping windows $R_n$ (black) and using weekly overlapping windows $R_t$ (blue). The lines and shaded areas represent the means and confidence intervals, respectively. (**D**) The portfolio effectiveness of each implementation $\eta _n$, which is defined as the ratio of mean ${\bar{R}}_{n-1}$ to ${\bar{R}}_n$. Note that y-axis is shown in log10-scale. (**E**) The intervention $\eta _n^{(m)}$ (in rectangles) and aggregated $\eta ^{(m)}$ (in circles) effectiveness of each intervention theme. The eight themes are *(i) Case identification, contact tracing and related measures*, *(ii) Environmental measures*, *(iii) Healthcare and public health capacity*, *(iv) Resource allocation*, *(v) Risk communication*, *(vi) Social distancing*, *(vii) Travel restriction*, and *(viii) Returning to normal life*. The blue (or red) color refers to the interventions that successfully reduced infections (or increment of infections). The log10-values of aggregated effectiveness of the six implemented themes were (i) − 0.16, (iii) 0.09, (iv) − 0.12, (v) 0.53, (vi) 0.18, and (vii) − 0.06, respectively.

The evaluation of transmission and intervention dynamics was first applied to Japan as the time delay is estimated using the data from Japan. Japan is the only country with reported reliable data of time delays from infection to symptoms and from symptoms to confirmation. Figure 2A,B show the cumulative and incidence curves of both infection and confirmation. The infection curve was obtained by iterating $r_c = 4$ times in the deconvolution process (see SI Fig. 24). We found that the first case was infected on 14-Jan-2020 (Day 0). The infection curve reached the peak on 2-Apr-2020 (Day 80) with 633 infected cases. The difference between two cumulative curves illustrates the time delay of about 12 days.

We initially considered 124 interventions implemented on $N = 49$ days from 21-Jan-2020 to 4-May-2020 in Japan. They were then categorized into 8 major themes and they became 79 intervention implementations represented by the black bars in Fig. 2E. Further, the first intervention on 21-Jan-2020 was discarded due to a low number of cumulative cases to estimate the reproduction number. As a result, 78 interventions implemented on $N = 48$ days were included in this study (see SI CSV file and the original data^20,21^ for further details).

Both non-overlapping $R_n$ and overlapping instantaneous reproduction numbers $R_t$ were estimated using the reconstructed infection curve (Fig. 2C). The mean of initial reproduction number $R_{n=0}$ was 2.26 from 14-Jan-2020 to 22-Jan-2020. The reproduction number was high at the beginning, and reached the maximum $R_{n=10} = 2.58$ on 6-Feb-2020. The reproduction number mostly kept above one for two months (Feb-2020 and Mar-2020) and dropped below one, $R_{n=41} = 0.93$ from 6-Apr-2020. After the last implementation on 4-May-2020, $R_{n=48} = 0.76$ stayed constant for a month until 7-Jun-2020. Additionally, we found that the overlapping $R_t$ grew above one and reached $R_t = 1.16$ on 22-May-2020, which $R_{n=48}$ could not capture.

As the non-overlapping reproduction numbers $R_n$ varied over time, the portfolio effectiveness was evaluated with a large variation shown in Fig. 2D. Fluctuations were large at the beginning and relatively stable in terms of magnitude at a later stage. Of 48 implementations, 30 were effective with $\eta _n > 10^0 = 1$. All intervention implementations from 19-Mar-2020 to 1-May-2020 were effective as the corresponding reproduction numbers were decreasing. However, after the reproduction number crossed the critical value of one, the last two implementations did not effectively suppress infections from 2-May-2020 with $\eta _{47} = 10^{-0.06}$ and $\eta _{48} = 10^{-0.14}$.

The intervention and aggregated portfolio effectiveness of each intervention theme was evaluated for each portfolio implementation and for the whole time period (Fig. 2E, circle and squares, respectively). The theme *(v) Risk communication* was implemented 19 times, and it was the most implemented intervention; while *(ii) Environmental measures* and *(viii) Returning to normal life* were not implemented in Japan. In general, there were more interventions implemented at earlier periods, in Feb-2020 and Mar-2020. In particular, there were 4 intervention themes at most implemented on the same day, the 13-Feb-2020 and 18-Mar-2020, and those were assumed to contribute equally to the calculation of their intervention effectiveness. Of 78 interventions, 44 were effective with $\eta _n^{(m)} > 10^0 = 1$, where the vast majority of them belonged to the theme *(v) Risk communication*. The corresponding aggregated effectiveness of risk communication was the highest with $\eta ^{(m=v)} = 10^{0.53}$. In contrast, the theme *(i) Case identification, contact tracing and related measures* was the worst performing with $\eta ^{(m=i)} = 10^{-0.16}$.

### Other country’s intervention effectiveness

**Figure 3:**
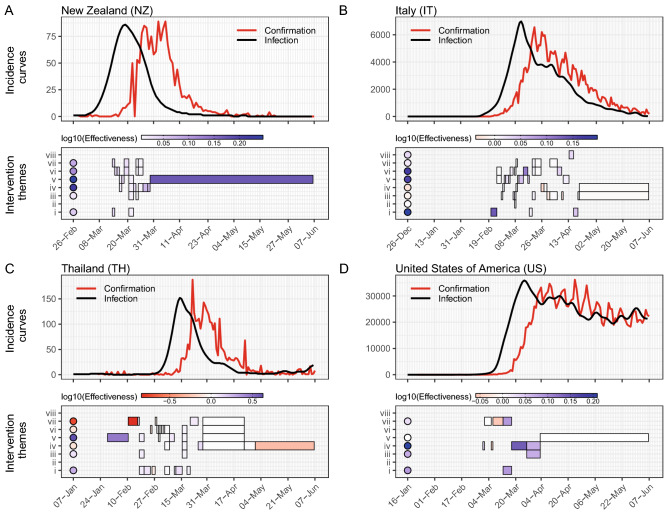
Intervention effectiveness for four prototypical countries. The upper panels of subfigures show the incidence curves of confirmation (red) and infection (black) in (**A**) New Zealand, (**B**) Italy, (**C**) Thailand and (**D**) United States of America. The lower panel of each subfigure shows the intervention $\eta _n^{(m)}$ (in rectangles) and aggregated $\eta ^{(m)}$ (in circles) effectiveness of each intervention theme. The eight themes are *(i) Case identification, contact tracing and related measures*, *(ii) Environmental measures*, *(iii) Healthcare and public health capacity*, *(iv) Resource allocation*, *(v) Risk communication*, *(vi) Social distancing*, *(vii) Travel restriction*, and *(viii) Returning to normal life*. The blue (or red) color refers to the interventions that successfully reduced infections (or increment of infections).

**Figure 4:**
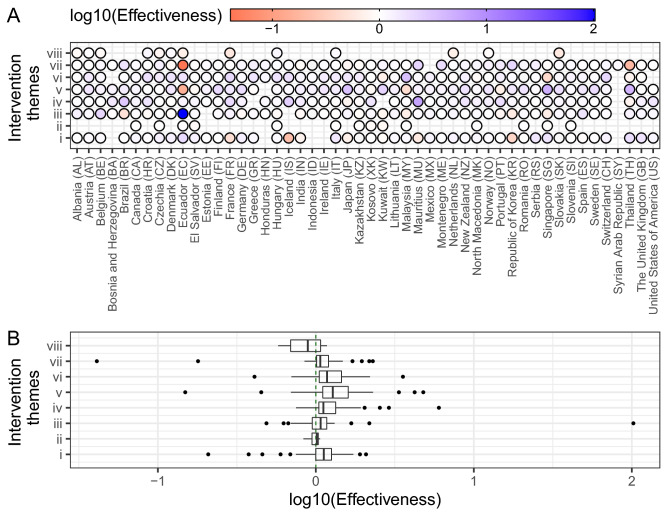
Total confirmed cases as a function of the fraction of effective portfolios. The fraction of effective portfolios is defined as the number of positive intervention sets considering the total. 50 countries are shown in red circles with their ISO codes. The black line and gray area represent the regression line and corresponding confidence interval, respectively. Note that the total confirmed cases on y-axis is shown in log10-scale.

We selected four prototypical countries: New Zealand, Italy, Thailand and United States of America (Fig. 3). We found that all intervention portfolio implemented in New Zealand were effective and thus incidence dropped gradually after reaching the peak at 18- and 19-Mar-2020. Italy is one of the four countries implemented all eight intervention themes. All except *(iv) Resource allocation* were effectively reduced transmission. We also found that Thailand effectively implemented *(v) Risk communication* interventions with $\eta ^{(m=v)} = 10^{0.64}$, which is the second highest ranked after Singapore (SI Fig. 52). However, the mean value of portfolio effectiveness is extremely low (second lowest, see SI Fig. 54) mainly due to a significantly increment of infections during the earlier stage with inefficient control of *(vii) Travel restriction*. The US as the country with the highest case number was not able to control transmission or suppress incidence as other three countries. There were only half of the implemented portfolios being effective, which was the third lowest among all 50 countries. It demonstrated a negative relationship between the total confirmed cases and the fraction of effective portfolios (Fig. 4). The fraction of effective portfolios is the number of positive intervention sets divided by the total number of implemented portfolios.

**Figure 5:**
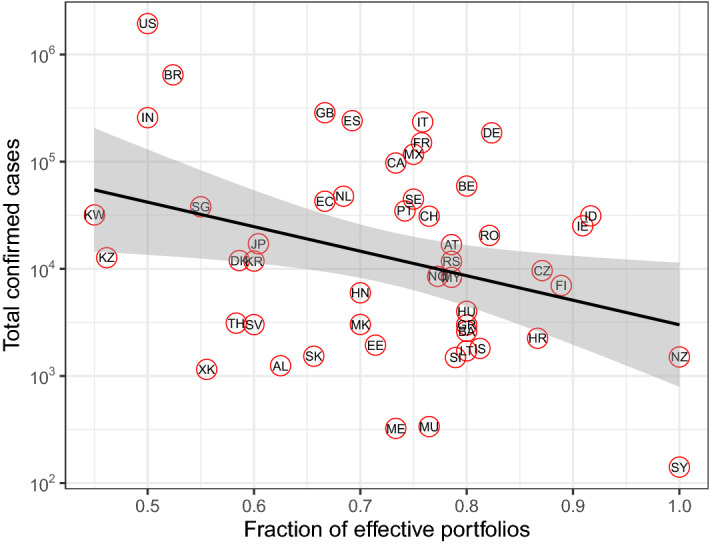
Aggregated portfolio effectiveness of intervention themes implemented in 50 selected countries. The eight themes are *(i) Case identification, contact tracing and related measures*, *(ii) Environmental measures*, *(iii) Healthcare and public health capacity*, *(iv) Resource allocation*, *(v) Risk communication*, *(vi) Social distancing*, *(vii) Travel restriction*, and *(viii) Returning to normal life*. (**A**) The blue (or red) color represents the interventions that successfully reduced infections (or increment of infections). (**B**) The boxes shows the distributions of the aggregated effectiveness in log10-scale. The median values of the eight themes were (i) 0.05, (ii) 0.01, (iii) 0.03, (iv) 0.05, (v) 0.10, (vi) 0.06, (vii) 0.03, and (viii) − 0.05, respectively.

The aggregated effectiveness of each intervention theme in 50 countries were estimated and shown in Fig. 5. Similar to Japan, most countries implemented all intervention themes but *(ii) Environmental measures* and *(viii) Returning to normal life* were the least implemented. Only Czechia, Ecuador, Hungary and Italy were the countries that explicitly implemented all 8 themes. Note that these findings are just based on officially reported interventions and do not consider interventions already realized independently of COVID. We discarded the earlier implementations in the Syrian Arab Republic and considered only one due to the uncertainty in estimating reproduction numbers. Figure 5A shows that Ecuador implemented the most and least effective interventions but this was caused by the sudden and suspicious incidence rise that is believed to be a surveillance malfunction. All themes but *(viii) Returning to normal life* were effective interventions as manifested by the positive median values (Fig. 5B). Specifically, the theme *(v) Risk communication* was the best intervention with the median of $\eta ^{(m=v)} = 10^{0.10}$, while *(vi) Social distancing* and *(iv) Resource allocation* were the second and third best interventions with the median at $10^{0.06}$ and $10^{0.05}$. In contrast, the theme *(viii) Returning to normal life* increased infection with a median value of effectiveness at $10^{-0.05}$. Among all interventions, *(ii) Environmental measures* was the one with the smallest variance. The geographic distribution of aggregated effectiveness of each intervention theme can be found in SI Fig. 55.

### Uncertainty and sensitivity

#### Surveillance uncertainties

In order to quantify potential surveillance fallacies related to systematic and suspicious fluctuations (treated as noise), we performed a sensitivity analysis and reconstructed the infection curves using an alternative setting, in which the uninformative initial condition (baseline setting) in RL iterative deconvolution process is replaced by back-shifted confirmation curves. The alternative infection curves are spiky with noise due to the direct usage of the surveillance data. SI Figs. 1–50 show full estimates obtained by two different settings. Taking Japan as an example, the estimates are similar to the baseline setting. The incidence reached the peak on 2-Apr-2020 with 811 cases and the reproduction number dropped below one from the early April 2020. Generally for all countries, the effectiveness is sensitive to the initial condition and this is an expected behavior of complex system processes that are highly dependent on the starting point of analyses. The magnitude of effectiveness is larger than the baseline setting due to higher fluctuations in incidence curves and reproduction numbers. As the baseline scenario for Japan, the themes *(v) Risk communication* and *(i) Case identification, contact tracing and related measures* were the best and worst interventions.

The aggregated effectiveness of each intervention theme given the alternative initial condition distributes slightly different than the baseline scenario (SI Fig. 51). Nevertheless, the first and second ranked intervention theme were *(v) Risk communication* and *(vi) Social distancing*. The theme *(viii) Returning to normal life* remained not effective. However, *(i) Case identification, contact tracing and related measures* and *(ii) Environmental measures* surprisingly showed a negative effectiveness in the alternative scenario; a result that underlines how these interventions may be erroneously evaluated in presence of strong surveillance uncertainties, also because their portfolio effectiveness is not very high.

## Discussion

In this study, we assessed the impact of various interventions implemented during the pandemic in 50 countries. Interventions are grouped into eight themes and evaluated in terms of their effect on daily incidence reduction. We employed the deconvolution method to reconstruct the incidence curve of infection, estimated the effective reproduction number of each implementation using the EpiEstim package, and evaluated the portfolio, intervention and aggregated effectiveness. In response of the destructive pandemic, all governments have taken as many interventions as possible to slow down the spread of the COVID-19 infections. However, it is important to design a set of interventions whose organization maximize their systemic effectiveness in reducing total cases. This is by far a non-trivial question considering the many interventions that are feasible and country specific underlying socio-environmental conditions. However, the important question is also about how effective each intervention is overall independently of fine scale details.

Back-calculation of infection curves is important as it reveals the “true” incidence curves on which effectiveness should be calculated. Deconvolution or back-calculation methods are extremely popular in many areas of science (e.g. in rainfall-runoff processes for instance) where the interest is to reconstruct the primary forcing of an effect and to attribute the variability of forcing to effects (i.e. interventions in our case). The calculation of effectiveness can be done on confirmation curves; however, conceptually, interventions aim to decrease infections and yet it is important to evaluate their impact on the primary population outcome without secondary effects related to incidence reporting that constitute confirmation curves. There are many other factors affecting confirmation and some of these factors have little to do with interventions. Additionally, confirmation curves contain a lot of spurious noise that is removed when back-calculating (inferring) infection curves for evaluating effectiveness. We stopped our back-calculation when we reached a feasible convergence defined by the simultaneous minimization and maximization of the root-mean-square difference (RMSD between incidence curves) and its curvature, respectively (see “Inference of incidence curves of infection with the Richardson-Lucy deconvolution” section). Then, the back-calculated incidence curve is the curve that allows one to predict the confirmation curve with the highest accuracy given the symptomatic-to-confirmation distribution as a transfer function. This infection curve is univocally defined among many other suboptimal infection curves. Ricci curvature is proportional to system?s entropy and this impacts predictability that is the highest when the curvature is at its minimum^33^. Precious studies showed that the maximum curvature corresponds to the highest information flow (in this case between curves) and this maximizes the portfolio effect for which the highest systemic risk is observed (in our case study it is about infection growth). Yet, effectiveness can also be interpreted as the relative ratio between curvatures to identify the most impacting interventions. The one with the highest curvature ratio is the one that is the most effective. Curvature, in the broad sense, is a measure by which a geometrical object deviates from being flat. In the context of networks, “flatness” reflects the connectivity and interdependence between distant nodes (in this case can be between curves or interventions, while in a spatially explicit context it can be between communities, populations or individuals).

### Non-pharmaceutical intervention effectiveness

Below we discuss about each intervention based on their median values of aggregated effectiveness irrespective of country’s details as synthesized by Fig. 5B. Some of these interventions target population or individual behavior, environmental exposure factors, health capacity and monitoring, or other non-health system resources. Further details about each intervention theme in each country can be found in^20,21^. Specifics of these interventions are provided in Supplementary Information with explicit descriptions of actions and links to each implemented action.

We validated the very high influence of risk communication for case reduction in all 50 countries considered. This is a quite striking result considering the wide differences in socio-environmental factors as well as intervention portfolios in each country. This result emphasizes that information in general, enhanced by any risk communication or other intervention implementation, is effectively linking all interventions directly or indirectly. For example, the announcement of travel alerts and warnings is a risk communication intervention that has an effect on human mobility which is categorized as “travel restriction”^34^. Risk communication, that can inform about any other intervention such as the use of face masks (conceivable as an “environmental intervention”) or provide broader COVID-19 information about potential transmission, is in general not enforcing any behavior on individuals but having a large impact on populations. Risk communication is particularly effective in Japan, Singapore and Thailand as empirically found from the media.

Social distancing is the second high scored intervention. Data show that mass gathering cancellation and closure of educational institutions have been efficient to curb the spread of infection. Other studies analyzed some specific effects of social distancing in reducing COVID-19 morbidity in the US and European setting^35,36^. These results confirm the high effectiveness of this intervention aimed to control population behavior in a more defined and strict sense than risk communication. Effectiveness of social distancing is positive across all countries, with the exception of Kuwait, with peaks for France, Malaysia and Switzerland.

The third ranked theme in terms of effectiveness is resource allocation to systems that are not related to the health system (considering both public health surveillance and healthcare). Interventions within this theme are in the form of individual aid and support to government organizations, such as: state aid, taxation and social security (e.g. the COVID-19 aid given to individuals and businesses in Japan and USA); support to police and army interventions to ensure the enforcement of individual restrictions such as mobility bans; and resources for crisis management plans and business continuity plans, including cost payments associated to emergency law formulation. Overall, resource allocation resulted quite effective for all countries. Brazil, Iceland and Mauritius seem to score high in this intervention category.

Case identification, contact tracing and related measures is the fourth ranked intervention theme and the first among specific public health intervention. Active contract tracing of infection clusters also contributed to case identification. As a result, more infections are identified. For example, Japan did not adopt the suggestion of massive individual tracing as suggested by WHO^37^, but performed targeted test to people with the most severe conditions^38,39^. The highest incidence in the early stage of the epidemic was considered and individuals traced after cluster identification. Likely, this lack of massive tracing implied that the effectiveness for this intervention in Japan is negative while for other prototypical countries, such as USA and Italy, is positive. Case identification and contact tracing effectiveness is also negative for European countries with elevated incidence such as France and Germany, as well as Asian countries such as India and South Korea. The last one was initially praised as a great example in contact tracing but a second wave hit the country and that reduced the effectiveness of tracing.

Travel restrictions show one of the highest fluctuations of effectiveness considering all interventions. In the case of Japan, travel restrictions in the form of entry ban for people with travel history to/from China, at the early stage of the pandemic, did not produce noticeable effect. One of the potential explanations could be about the implementation timing of the entry ban when China already started a strict mobility restriction within the country, including the complete lockdown of Wuhan, the epicenter of the pandemic. The later implementation of the entry ban in late March for people from Europe and Americas had a positive effect in mitigating the magnitude of the second infection wave (due to imported cases). Overall, for travel restriction in Japan the average effectiveness is very similar to zero due to the initial negative and later positive effects. A similar situation is experienced by many countries such as UK, USA, and Italy. Additionally, other countries show similar time-varying effectiveness of Japan where the reduction of incidence instantaneously occurred upon implementations but the aggregated effectiveness is not one of the highest. Thailand in contrast showed a negative effectiveness likely because of the very mild total incidence on which this intervention was supposed to have an effect. These results corroborate the idea that just travel restriction is not enough in abating drastically the transmission but many other interventions are needed.

Healthcare and public health capacity include a set of interventions strongly focused on the healthcare system and predominantly the following actions: increase in medical supplies and equipment; increase in healthcare workforce; increase of isolation and quarantine facilities; increase supply of personal protective equipment (including face masks); enhanced laboratory testing capacity; increase patient capacity; and healthcare research. Healthcare and public health capacity was expected to have played a vital role in reducing incidence, but our results did show this intervention was as effective as other interventions. This results can actually be well explained considering a triplet of factors. The first one is about the fact that healthcare is predominantly about post infection treatment and does not reduce infections in the population; thus, even in countries where healthcare capacity is extremely high, such as in USA, effectiveness of this theme is low. The second factor is related to the likely increase in risk of cross-infection in hospitals and other healthcare facilities, once suspected, asymptomatic and and infected individuals are present in great numbers and get in contact with other people. This may occur even when patient capacity increases. The last factor is about the portfolio effect for which the effectiveness of one intervention is also a function of all others. Due to the high interdependence of all other interventions, healthcare and public health capacity seems more independent and yet it exhibits a different trend considering its effect on case reduction. Based on our results, Brazil, India, Malaysia, Portugal, South Korea and Singapore are scoring the lowest for this theme effectiveness. Strangely, Ecuador has a very high effectiveness but this seems related to the large reduction in cases, after a peak of about 10,000 or higher, where resource allocation to healthcare and public health capacity was the last intervention implemented.

The last type of direct interventions are environmental measures in the form of actions aimed to reduce exposure to *Coronavirus*. These actions include: approval of new biocidal products; and environmental cleaning and disinfection occurred in public spaces such as public transports, public buildings (schools, hospitals, government offices, etc.), and grocery stores. We observe that only 13 out of 50 countries implemented environmental measures. This is because many environmental measures are already adopted by different countries independently of COVID, such as in Japan^40^, and because in reality many countries did not target exposure reduction as a valuable intervention for reducing COVID-19 transmission. Many studies appeared in the scientific community about *Coronavirus* lifetime on different surfaces^41–43^ but these results generated more risk communication^44,45^ than acting directly on spreading media. Yet, part of information and effectiveness related to environmental measures is contained in risk communication.

The last intervention theme considered is “returning to normal life”, that is relaxing or removing some of other intervention themes adopted previously. Thus, partial lift of the curfew, quarantine, and landing bans; conditional re-opening of activities (certain shops, cultural institutions, places of worship, touristic sites, and short-term accommodations), conditional lift of restriction on gathering, special relief for movements, and reopening land borders are are types of actions seen in the countries considered. Data related to returning to normal life actions are not available for many countries due to the fact many countries are still under rigid measures for containing COVID-19 spreading. In some countries, like Slovakia and the Netherlands, the epidemic was believed to be effectively suppressed because daily new incidence appeared in continuous declination. Therefore, local governments loosened restriction policies to allow people to return to normal life. However, a second wave of cases was recorded and this is the reason for which effectiveness of returning to normal life is negative for this countries. To a certain extent secondary new cases are expected after relaxation of constraining interventions, but any relaxation should be handled carefully due to the highly probability of resurgence of local outbreaks is as shown in Ecuador, Norway, and more recently in USA and Japan. There is certainly the need to loose some of intervention limitation but a complete return to normal life as it was without COVID-19 is almost impossible at least in short term. *Coronavirus* is not going to disappear and it is circulating in the environment where millions of people live. Yet, it is our belief that a portfolio approach is needed where COVID-19 becomes part of our life and interdependent interventions aim to educate us toward behaviors that reduce exposure and transmission.

By comparing all interventions it is worth noticing that the most loose intervention in terms of enforcing behavior on individuals, i.e. risk communication, is the most effective on populations. This is related to the fact that risk communication is addressing many other interventions and educating population across the spectrum of elements of risk, i.e. hazards, exposures, vulnerability and controls. This also supports the belief that stringent controls on populations have psychology-driven negative feedbacks of generating the opposite behavior of what is enforced^46^. In other words, coercion seems to increase the number of defectors^47^ and this may be one of the many interpretations for describing the lower effectiveness of behavior-constraining interventions. In a modern portfolio (Markowitz) perspective it is interesting to note that mean effectiveness of intervention scales with its variance (SI Fig. 53). Thus, the most effective interventions has also the largest variability. By analyzing portfolios for all countries it is possible to reconstruct an empirical and discrete Pareto frontier that maximizes and minimizes mean and variance of effectiveness (SI Fig. 54). Countries along the Pareto frontier are theoretically the ones with an optimal portfolio considering the occurred incidence; the further one country is from the Pareto frontier the worst the collective portfolio and yet all intervention effectiveness. As for COVID-19, New Zealand and Thailand are the most optimal countries, while USA and Brazil are the least optimal in terms of portfolio effectiveness. Japan and Italy are instead suboptimal countries with respect to their total incidence and show a vey diverse organization of interventions over time: Japan has one of the largest number of implemented intervention but restricted to 6 themes only, and Italy has a more regular but delayed set of interventions considering all feasible 8 themes. This emphasizes how organization of implemented interventions (i.e., portfolio over time) is primarily important vs. the diversity of the portfolio. Previous portfolio applications have considered these issues, also when facing multiple diseases simultaneously^8^, that can be considered more practically for COVID-19 emergency management.

It should be kept in mind that some of these interventions are not quite easily implementable rapidly during a pandemic like with COVID. In particular, environmental interventions more broadly defined are related to underlying socio-environmental features and that is establishing the vulnerability determining the initial or baseline risk of a population^40^. Nonetheless, environmental interventions are important because they reduce exposures dramatically but unfortunately not many countries implemented these interventions.

### Comparison with other studies

**Figure 6:**
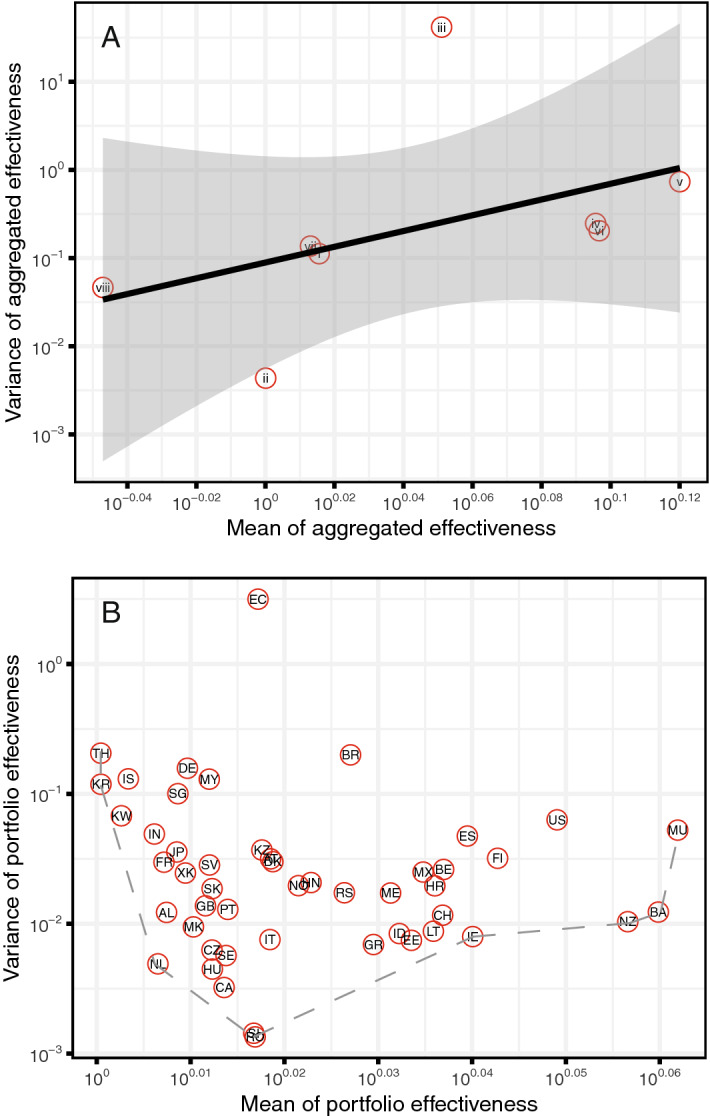
Mean and variance of portfolio effectiveness for each NPI and for countries. (**A**) Mean and variance of aggregated effectiveness across time and countries; 8 themes are shown in red circles. The black line and gray area represent the regression line and corresponding confidence interval, respectively. Note that both axes are shown in log10-scale. (**B**) Mean and variance of portfolio effectiveness. 50 countries are shown in red circles with their ISO codes. The gray dashed line represents an empirical Pareto frontier where variance and mean of portfolio effectiveness is the risk level and the expected return, respectively. Note that both axes are shown in log10-scale.

Our paper is the closest to the approach of^10^ since we used the same dataset and a conceptually similar model. In^10^ the authors interestingly tested four different statistical models on three datasets (among which one dataset is the one we used) and found a correlation between the entropy of the distribution of NPI occurrences (in the normalized ranking of countries) and site-specificity of interventions: high values of the normalized entropy signal that the performance of NPIs depends largely on the geographical region considered. The effectiveness varies considerably across countries, and yet the predictability of intervention effects is much smaller without knowledge of other specific factors, e.g. compliance, and other factors. These high entropy interventions have typically small effectiveness. Conversely, NPIs have a “universal performance” for entropy close to zero; this means that effectiveness is stable across countries independently of the specific details, and that increases the predictability of intervention effects at the macroscale. This is for instance the case of risk communication^10^ also implicitly recognized the idea of intervention portfolio by reconstructing a network of co-implementation but without quantitatively assess the joint effectiveness of NPIs as in our work. On the contrary of our findings^10^ showed that the most effective measures include closing and restricting most places where people gather in smaller or larger numbers for extended periods of time (businesses, bars, schools and so on). Note that, however, these interventions are the byproduct of risk communication strategies. This result (i.e., the most effective measures are closing and restricting access to gathering places) holds when NPIs are accounted in isolation but not in a portfolio perspective that is when all other NPIs are considered simultaneously. Haug et al.^10^ also find a number of highly effective NPIs that can be considered less costly. For instance, as much as in our study, they found that risk-communication strategies feature prominently amongst consensus NPIs. This includes government actions intended to educate and actively communicate with the public, including about social distancing interventions. Haug et al.^10^ considered a more specific analysis of interventions but found similar conclusions. “Level 2” risk communication in order of effectiveness are: educate and actively communicate with the public, travel alert and warning, and actively communicate with managers. Level 3 risk communication in order of effectiveness are: call for return of nationals living abroad, promote social distancing measures, promote workplace safety measures and self-initiated isolation of people with mild respiratory symptoms, encourage self-initiated quarantine, information about travels, and warning against travel to and return from high risk. The simultaneous consideration of many distinct NPI themes allows us to move beyond the simple evaluation of individual NPIs to assess; the collective choice of specific sequences of interventions impacts the systemic effectiveness of the whole intervention portfolio and of each NPI. Additionally we propose a method to rank countries based on the pair mean-variance effectiveness (Fig. 6)—to maximize and minimize, respectively—that is a measure of absolute performance independently of total cases experienced by a country, in relation to the distance from an optimal Pareto frontier. The (Pareto) optimal NPI frontier (i.e. the set of optimal intervention portfolios which can be constructed empirically as in our case in Fig. 6) can inform about interventions to undertake in other countries in order to maximize effectiveness locally and globally. This is also our novelty compared to Haug et al.^10^. Overall our estimated effectiveness of risk communication is $\sim$ 23% that seems much more realistic than the 48% predicted by Haug et al.^10^. We believe Haug et al.^10^ does not truly capture the portfolio effect because it estimates interventions in isolation (by removing them one by one, that creates time series of cases dependent on interactions of all other interventions only and that is dynamically dependent on time) leading to overestimation of intervention effectiveness. Other papers such as Wu et al.^48^ used a data-driven mechanistic Bayesian model to quantify the effect of NPIs based on observed US fatalities; stay-at-home orders and advisory were estimated as the only effective NPI in metropolitan and urban counties, confirming the importance of risk communication. Other studies were just focused on one NPI or country^49–53^, thus it is hard to draw comparisons.

### Assumptions and potential limitations

One caveat of the study is that effectiveness is strictly a predictable change in reproduction number over time than “true effectiveness” whether that is truly measurable. For instance, surveillance exhibits a negative effectiveness (that is, an increase) on $R_t$, presumably related to the fact that more testing allows for more cases to be identified. Obviously this is a good thing because more knowledge of events leads to better decisions but numerically this corresponds to a positive change in $R_t$, yet to a negative effectiveness. This also happens in^10^, thus a lot of care is needed when interpreting results and as with any model true causality and predictability do not necessarily coincide^54^. Further sensitivity analysis proved that results (patterns of effectiveness) are minimally sensitive to distributions of symptomatic-to-confirmation periods. This low sensitivity to epidemiological infection-to-confirmation distribution also underlines the dominance of few strong NPIs in the intervention portfolio and the second-order importance of all other NPIs. The atemporal *null hypothesis* would be that no heterogeneous patterns of effectiveness emerge (for any country and among countries) without the diverse effect of interventions. Another source of uncertainty coming from data and the model is related to the simultaneous co-occurrence of interventions, for which nobody can precisely attribute the relative effectiveness of each NPI theme for the same time *t*. This is also considering that NPI portfolio effectiveness at time *t* is dependent on time $t-\delta$ where $\delta$ is temporal gap into the past; in other words, contingency matters but we verified how close the overlapping $R_t$ (when $\delta$ is considered) is to the non-overlapping $R_t$. For this complexity inexplicability we decided to adopt a minimalist pattern-oriented model that divides equally the effectiveness of interventions for those co-occurring at the same time step. The temporal *null hypothesis* in this case is that if all interventions are equal then no heterogeneous patterns of effectiveness would arise at any time step and for the whole period. Any assumption (extra factor, e.g. compliance that is already embedded into the signature of infections and confirmations) on the attribution of effectiveness would just bias the inferred effectiveness because: (i) it is extremely hard to measure specific factors especially at the beginning of the epidemic; and (ii) these factors are highly country dependent and would involve the use of other more uncertain datasets. All extra factors affecting effectiveness as the predicted macroscopic variable are already embedded into the data from which we want to derive effectiveness patterns. These null-hypotheses are equivalent to the “what if?? scenarios?? of^10^ where the authors, rather than attributing effectiveness equally to co-occurring interventions, performed predictions by creating artificial sequences of NPIs (by selectively deleting one NPI at a time from all sequences, whose effectiveness to be estimated) in each country and comparing them to the actual sequence. However, the removal of one intervention does not remove the effects (due to interactions) that intervention has on others. Systemic effectiveness must consider the contingency determined by time-dependent co-occurrence of events (infections) and interventions. After removal of one intervention any future prediction is just speculative because that removal affects all others? effectiveness; yet, we believe portfolio approaches are better to capture estimates of non-linear effects.

## Conclusions

The proposed probabilistic model demonstrates the systemic impact of intervention portfolios from which distinct NPI effectiveness is inferred without a-priori assumption on NPI interdependencies. The model is uniquely applied to 50 countries to test intervention effectiveness defined as the impact on total case growth. The following critical findings are worth mentioning:

- A deconvolution model is proposed to reconstruct expected cases of infection from raw observations of confirmation considering surveillance fallacies (delays and potential under-reporting). The initial condition of the iterative procedure is established by a uniform infection curve that is flat and unbiased (i.e., a Maximum Entropy distribution). The expected infection curve, inferred by the iterative back-calculation procedure, is the one that maximizes the curvature of RMSD, or equivalently minimizes overfitting and the noise around the reconstructed infections. The maximization of the curvature does not correspond with the minimization of RMSD that is the expectation-maximization solution. The latter is focused on maximizing accuracy in reconstructing the observed infection curve while the former is concentrated in minimizing the noise of the estimated curve. The smooth infection curve (baseline setting) is the theoretical expectation about epidemic dynamics without extra stochastic forcing (such as surveillance fallacies and environmental fluctuations beyond time delays already included into the model). The alternative setting employing the back-shifted curve as the initial condition considers the delays directly and contains more pronounced spikes occasionally. Thus, the difference between the smooth curve (baseline setting) and noisy curve (alternative setting) gives an assessment of the effect of surveillance fallacies or other unexplained stochastic forcing. Therefore, the deconvolution model is also very useful for providing a macro-assessment of surveillance system efficiency beyond evaluating likely case magnitude and timing. This highlights the fact of considering systematic and specific uncertainties in data rather than forcing model to replicate data with the highest accuracy.
- According to our assessment in 50 countries, the overall performance of risk communication is the most effective to reduce case spreading, while returning to normal life would in contrast increase spreading of cases in the population. These results are concordant with current empirical evidence from many countries. Until the time when available pharmaceutical interventions would be available, countries should keep implementing the effective NPIs portfolios. Lifting all intervention measures at the same time should be avoided to prevent second epidemic waves and that is, unfortunately, what is happening in many countries worldwide at the moment.
- Total confirmed cases are inversely proportional to the fraction of effective portfolios and the variance of effectiveness is related to its mean in a Pareto optimality framework. This allows one to rank countries based on the pair mean-variance effectiveness (to maximize and minimize, respectively) that is a measure of absolute performance independently of total cases. We observe that the diversity of interventions is the highest for intermediate mean portfolio effectiveness with large variance. New Zealand and Italy are the countries with the highest and intermediate effectiveness fraction despite scoring low for risk communication; New Zealand because with comparatively low cases it reduced the epidemic with few well planned interventions while Italy with very high cases controlled the epidemic with many interventions (all eight themes). The fraction of effective portfolios in Thailand is similar to Italy but it shows many ineffective interventions compared to the total case magnitude. Nonetheless, Thailand and Japan show the highest risk communication effectiveness. USA shows the worst performance in terms of total cases and portfolio effectiveness. These results manifest the idea that multiple optimal portfolio exist and their implementation is highly dependent on the country specifics (embedded into the infection dynamics) which also determines the systemic effectiveness based on the total cases despite each specific intervention effectiveness is more robust. This is the reason for which we find less variability of intervention effectiveness than when comparing countries. In general, portfolio effectiveness is highly dependent on timing, sequence, diversity and spatial distribution of interventions in order of importance. A non-linear relationship between risk communication effectiveness and total cases is observed and this is a sign that the portfolio is crucial in determining cases rather than one single intervention despite on average risk communication has the highest performance.

In conclusion, like Haug et al.^10^ concludes, we highlight the suggestion to countries to really tailor risk communication strategies that educate people and adopt them since the very early stages of the epidemic. These interventions seem to have a much higher general validity independently of country specifics and likely foster better compliance. When infection start to surge country specific more restrictive interventions should be implemented.

## Materials and methods

### Incidence curve of case confirmation

The incidence value $I_t$ on Day *t* is the difference between cumulative confirmed cases of Days *t* and $t-1$, i.e.,

1 $$\begin{aligned} I_t = C_t - C_{t-1}. \end{aligned}$$

In surveillance data negative incidence may exist due to misreported cumulative numbers with a decreasing trend. A cumulative curve must be a monotonically increasing function. Suppose there exists $t_0$ such that $I_{t_0} = C_{t_0} - C_{t_0-1} < 0$. We assume the cumulative case number on Day ($t_0-1$) was misreported and led to the negative incidence on Day $t_0$. In this case, $C_{t_0-1}$ is discarded and $I_{t_0} + I_{t_0-1} = C_{t_0} - C_{t_0-2}$, where $I_{t_0}$ and $I_{t_0-1}$ are further assumed to be the same. Note that incidence is an integer and the reminder is distributed on Day ($t_0-1$). In other words, the reconstructed part is monotonically decreasing. Further if $C_{t_0} - C_{t_0-2} < 0$, we assume that $C_{t_0-2}$ is misreported and discarded. The process is repeated backward in time until the cumulative curve is a monotonically increasing function, i.e. all incidence is non-negative. The proportion of misreported data in all countries are summarized in Table S1.

### Time delay from infection to confirmation

The time delay of infection-to-confirmation was split into two: infection-to-symptomatic (i.e. incubation period) and symptomatic-to-confirmation delays. First, the incubation period follows the gamma distribution with the shape and scale parameters being 5.807 and 0.948, respectively (the corresponding mean and SD were 5.5 and 2.3 days)^28^. Second, the time delay distribution of symptomatic-to-confirmation was estimated using maximum likelihood method (MLE). Precisely we used optim in R. We assume the time of symptomatic-to-confirmation follows a log-normal distribution and fitted by the dates of symptom onset and confirmation of individuals. Here, we used 8917 pairs of dates, from a local dataset consisting of 17,131 individuals from Japan^32^. Both distributions were further discretized by $f(\tau ) = F(\tau +1) - F(\tau )$, for $\tau = 0, 1, ..., \tau _m$, where *f* and *F* are probability mass function (PMF) and cumulative distribution function (CDF) of gamma and log-normal distributions, respectively. Here, we took $\tau _m=14$ for incubation period and $\tau _m=21$ for symptomatic-to-confirmation. The distributions were normalized afterwards. The distribution of infection-to-confirmation was obtained by the convolution equation, with the assumption of the two distributions being independent, i.e.

2 $$\begin{aligned} f^{ec}(t) = \sum _{\tau =0}^{t} f^{es}(t) f^{sc}(t-\tau ), \end{aligned}$$

for $t = 0, 1, ..., 35$ and where $f^{es}(t)$ and $f^{sc}(t)$ are normalized PMF of incubation period and symptomatic-to-confirmation, respectively. The superscripts *e*, *s* and *c* represent infection, symptomatic and confirmation.

### Inference of incidence curves of infection with the Richardson-Lucy deconvolution

The incidence of infection was back-calculated using Richardson-Lucy (RL) deconvolution method, which was previously used in other studies for reconstructing the incidence curve of influenza^23^. Let $I_t$ and ${\hat{I}}_t$ be the incidence of confirmation and infection on Day *t*, respectively. Suppose that the confirmation curve $I_t$ was observed from Day 1 to Day *T*. We estimated the infection curve from Day $-34$ to Day *T*. The RL iteration method updates a sequence from an initial curve ${\hat{I}}_t^{(0)}$, i.e.

3 $$\begin{aligned} {\hat{I}}_t^{(r+1)} = {\hat{I}}_t^{(r)} / q_t \sum _{{\tilde{t}}=t}^{T} \frac{p_{{\tilde{t}}-t} I_{{\tilde{t}}}}{\sum _{\tau =0}^{{\tilde{t}}} p_{{\tilde{t}}-\tau } {\hat{I}}_\tau ^{(r)} / {\hat{q}}_\tau }, \end{aligned}$$

for $t = -34, ..., T$ and $r = 0, 1, ...$. The time delay $p_t$ was the function of infection-to-confirmation $f^{ec}(t)$. Following the original approach, the probability of a case of infection on Day *t* and being reported after or on Day 1 is

4 $$\begin{aligned} q_t&= \left\{ \begin{array}{ll} \sum _{\tau =-t+1}^{35 } p_\tau &{}< 1 \text{ for } t = -34, ..., 0 \\ \sum _{\tau =0 }^{35 } p_\tau &{}= 1 \text{ for } t = 1, ..., T-35 \\ \sum _{\tau =0 }^{T-t} p_\tau &{}< 1 \text{ for } t = T-34, ..., T \\ \end{array}\right. . \end{aligned}$$

However, we note that the first case was confirmed on Day 1. We assumed that there were no unobserved confirmed cases before Day 1 and thus we modified the original approach by adding ${\hat{q}}_t$ in the convolution process. The observation probability is

5 $$\begin{aligned} {\hat{q}}_t&= \left\{ \begin{array}{ll} \sum _{\tau =-t+1}^{35 } p_\tau &{}< 1 \text{ for } t = -34, ..., 0 \\ \sum _{\tau =0 }^{35 } p_\tau &{}= 1 \text{ for } t = 1, ..., T \\ \end{array}\right. . \end{aligned}$$

The initial condition ${\hat{I}}_t^{(0)}$ was chosen to be uniform with maximum entropy, that is the least biased distribution^27^. As an alternative, we performed a pseudo sensitivity analysis where the initial condition ${\hat{I}}_t^{(0)}$ was selected to be the back-shifted confirmation curve by 10 days as the mode of infection-to-confirmation distribution^23^. The earlier 25 days (Day $-34$ to Day 0) were assumed to be 1 and the latest 10 days (Day $T-9$ to Day *T*) were assumed to be same as the number of confirmed cases on Day *T*. The reconstructed infection curve is expected to be more informative (and more strongly related to surveillance systematic biases) while the curve using the uniform initial condition is smoother and corresponding to the theoretical noise-free dynamics. Another approach, called the smoothed EM algorithm and using weighted averages, can be available to obtain smooth incidence curves^22^. However, we did not used the approach in this work due to its lower stability.

The RL iterative process updates the infection curve from the initial curve ${\hat{I}}_t^{(0)}$ and is stopped after several iterations. We selected an optimal number of iterations $r_c$ via a composite stopping criterion, i.e. root-mean-square difference (RMSD) and its curvature^55^. The RMSD is defined as $\sqrt{\frac{1}{T+35} \sum _{t=-34}^{T} ({\hat{I}}_t^{(r+1)} - {\hat{I}}_t^{(r)})^2}$. The curvature of RMSD is defined as the anti-clockwise angle $\theta$ of RMSD at ($r-1$), *r*, ($r+1$). We stopped the iterative process at the maximum curvature to avoid overfitting while RMSD is minimized^55^. Note that we tested $\chi ^2$ statistic proposed in^23^; however, this test was not capable to terminate the iteration to the theoretically expected convergence due to data noise^27^.

### Effective reproduction number

The effective reproduction number, $R_t$, is the average number of secondary infections generated by one primary infection, is commonly used to measure disease transmissibility over time during an epidemic outbreak. Two estimators are commonly derived for the effective reproduction number. The instantaneous reproduction number ${\hat{R}}_t$ is defined as the ratio of the number of infections at time *t* to the expected number of infections generated by all infections prior to *t*; and, the cohort or case reproduction number ${\hat{R}}_t^C$ that is defined as the average number of secondary cases infected by the primary case who is infected at time *t*^56–58^. The former is more sensitive to the extrinsic interventions exhibiting a sudden rise or fall; in contrast, the latter varies smoothly and can be estimated retrospectively. Both estimators incorporate the probability distribution of generation time, which is the time interval between the primary and secondary infections. In practice, we estimated the instantaneous reproduction number because that is more related to interventions; ${\hat{R}}_t$ was calculated by using the EpiEstim package in R software^29,30^, which was used by some previous studies for the ongoing pandemic^24,31,59^. EpiEstim implements a Bayesian approach for quantifying transmissibility over time during an epidemic. More specifically, it allows estimating the instantaneous and case reproduction numbers during an epidemic for which a time series of incidence is available and the distribution of the serial interval (time between symptom onset in a primary and secondary case).

We assume that the reproduction number $R_n$ is a constant and non-overlapping over the period between implementations. Suppose there were *N* implementations and $N+1$ reproduction number $R_n$ for $n = 0, 1, ..., N$. The reproduction number $R_n$ represents the infection rate from Day $t_n$ to Day $(t_{n+1}-1)$, for $n = 1, ..., N-1$. The initial reproduction number $R_{n=0}$ and the final reproduction number $R_{n=N}$ represent transmission from first day to Day $(t_{n=1}-1)$ and from Day $t_N$ to last day, Day *T*, respectively. In practice, the reproduction number was estimated by the incidence of infection ${\hat{I}}_t$ using the R package EpiEstim^29,30^. We assume that the generation time was identical to the serial interval, which follows the gamma distribution with mean 4.5 days and SD 4.5 days^31^.

In addition to the non-overlapping $R_n$, we also estimated the daily updated reproduction number $R_t$ with an overlapping time window of one week^29^. As the estimates of reproduction number at the beginning of an epidemic is full of uncertainty due to low number of cases, the starting time of the estimated reproduction number $R_t$ was selected by three criteria^29^. The overlapping $R_t$ is estimated after at least one time window (7 days), one serial interval (4.5 days) and containing 12 cumulative cases. In our estimation of the non-overlapping reproduction number $R_n$, we discarded implementations earlier than the starting time of $R_t$ to make sure that the initial reproduction number $R_{n=0}$ with the least uncertainty.

### Effectiveness of interventions

In this work, we employed the data of intervention implementations from an open dataset where interventions adopted for COVID-19 by countries worldwide are listed^20,21^. We followed the reported categorization scheme to group interventions into 8 major themes:

(i): Case identification, contact tracing and related measures
(ii): Environmental measures
(iii): Healthcare and public health capacity
(iv): Resource allocation
(v): Risk communication
(vi): Social distancing
(vii): Travel restriction
(viii): Returning to normal life

Beyond these broad country-independent themes, three layers of details are available about country specific actions within each time as well a link reporting to further details (see SI CSV files for the selected interventions).

Note that interventions under the same themes were considered as identical, and hereafter, we refer the interventions as those that belong to the same themes. Further, we selected only national scale interventions. Country specific interventions within the same theme may differ in implementation from country to country but it is quite hard to define differences and consider them computationally since a long qualitative description is provided. However, the purpose of the study is to detect macroepidemiological signatures of effectiveness across broad country-independent interventions themes and leave to country specific decision making and policy their characterization and implementation. The latter must certainly take into account specific socio-environmental and economic factors while themes are important categories at the macroscale that can be used to compare countries and define strong intervention that are effective beyond country details.

In our evaluation, there are three measures of estimated effectiveness: (i) the temporal effectiveness for all interventions belonging to different themes implemented on the same day (*portfolio effectiveness*, hereafter, where a portfolio is a set of implemented interventions); (ii) the temporal effectiveness of each intervention belonging to one theme (*intervention effectiveness*, hereafter); and (iii) the aggregated effectiveness of each intervention theme across the whole time period (*aggregated effectiveness* or *aggregated portfolio effectiveness*, hereafter). This is done for each country separately considering the sequence of implemented interventions with a day-scale resolution. From one intervention portfolio implementation to another (on any subsequent day) we keep the previously calculated portfolio effectiveness as constant (also in consideration of the absence of information about the ending of interventions after implementation) and duration implementation is not taken into account. Note that we prefer the word ”effectiveness” rather than efficiency (that is typically used for one intervention only) because each intervention effect is truly non-separable from others and we do not take into account their cross-correlation explicitly; the latter is already embedded into the data, yet, any further assumption-based calculation would likely introduce estimation biases.

First, the *portfolio effectiveness* of implementation on Day $t_n$ is defined as the ratio of mean reproduction numbers:

6 $$\begin{aligned} \eta _n = \frac{{\bar{R}}_{n-1}}{{\bar{R}}_n}, \end{aligned}$$

for $n = 1, 2, ..., N$, where *n* is the index identifying the portfolio over time (*N* being the total number of portfolios), and ${\bar{R}}_n$ is the mean value of the reproduction number $R_n$. If the effectiveness $\eta _n > 10^0 = 1$, the portfolio on Day $t_n$ was effective to reduce transmission. Thus, the temporary reduction in reproduction numbers reflects the transient impact of the portfolio until the next portfolio implementation with one or more interventions.

Second, a portfolio is a combination of interventions, either for one time step or the whole period. We assume that the effectiveness of a portfolio is a product (as the multiplication rule in probability) of intervention effectiveness of all interventions implemented on the same day, i.e. $\eta _n = \prod _{m \in M_n} \eta _n^{(m)}$ or $\log _{10}\eta _n = \sum _{m \in M_n} \log _{10}\eta _n^{(m)}$, where $M_n$ is the set containing the themes *m* implemented on Day $t_n$. Without any prior information, we further assume that the interventions contributed equally to the portfolio. The corresponding *intervention effectiveness* for a theme *m* implemented on Day $t_n$ is then:

7 $$\begin{aligned} \log _{10}\eta _n^{(m)} = \frac{\log _{10}\eta _n}{\Vert M_n \Vert }, \end{aligned}$$

where $\Vert M_n \Vert$ denotes the number of interventions implemented on Day $t_n$. Thus, the *intervention effectiveness* is shared equally among all interventions for the day and subsequent time period until the next portfolio implementation.

Third, we assume that the *aggregated effectiveness* of an intervention theme *m* is the product (due to the log consideration of $\eta$) of *intervention effectiveness* of each theme across the whole time period, i.e.:

8 $$\begin{aligned} \log _{10}\eta ^{(m)} = \sum _{n} \log _{10}\eta _n^{(m)}. \end{aligned}$$

The % change in infections of each intervention is given by $(1-10^{-\log _{10}\eta ^{(m)}})*100$.

### Data and application to 50 selected countries

We applied the evaluation framework first to Japan (due to limited available data) using a local dataset that contains individual information^32^ and then to other 49 countries using two datasets from WHO^60^ and John Hopkins^61^. The 50 countries include five continents, Asia, Oceania, Europe, North and South America (see the maps in SI Fig. 55). Each country may have a different epidemic period depending on the first identified patient. All data were collected as of 7-Jun-2020 (Day *T*). The use of two dataset is important for checking potential mistakes due to inconsistencies. However, both datasets from WHO and John Hopkins contain a reporting delay from time of confirmation due to processes such as reporting from local to global agencies. We compared the both global datasets with local dataset from Japan and found that the reporting delay is about one and two days in the datasets from WHO and John Hopkins, respectively. The back-calculated process employed the time delay of infection-to-confirmation estimated using the local data from Japan and the reporting delay of either one or two days, depending on the selection of data sources. The incidence data of each country (except Japan) are taken from the one (either WHO or John Hopkins) with a smoother curve i.e. fewer potential mistakes. The entire modeling implementation including datasets of 50 countries can be found at https://github.com/imlouischan/corona-jp.

## Supplementary Information

Supplementary Information 1.

Supplementary Information 2.
