# Supplementary material for: COVID-19 non-pharmaceutical intervention portfolio effectiveness and risk communication predominance
Source: Sci Rep. 2021 May 19;11:10605. doi: 10.1038/s41598-021-88309-1 (PMC8134637; doi:10.1038/s41598-021-88309-1)
Supplement: Supplementary file 1 — Supplementary Information 1. [file 41598_2021_88309_MOESM1_ESM.pdf]

COVID-19 NON-PHARMACEUTICAL INTERVENTION PORTFOLIO EFFECTIVENESS AND RISK COMMUNICATION PREDOMINANCE (SUPPLEMENTARY INFORMATION)

LOUIS YAT HIN CHAN[1,2,3,*], BAOYIN YUAN[1,4], MATTEO CONVERTINO[2,5]

[1] GRADUATE SCHOOL OF MEDICINE, HOKKAIDO UNIVERSITY, SAPPORO, JAPAN [2] NEXUS GROUP, GRADUATE SCHOOL OF INFORMATION SCIENCE AND TECHNOLOGY, HOKKAIDO UNIVERSITY, SAPPORO, JAPAN [3] DEPARTMENT OF INFECTIOUS DISEASE EPIDEMIOLOGY AND MODELLING, NORWEGIAN INSTITUTE OF PUBLIC HEALTH, OSLO, NORWAY [4] SCHOOL OF MATHEMATICS, SOUTH CHINA UNIVERSITY OF TECHNOLOGY, GUANGZHOU, CHINA [5] INSTITUTE OF ENVIRONMENT AND ECOLOGY, TSINGHUA SHENZHEN INTERNATIONAL GRADUATE SCHOOL, TSINGHUA UNIVERSITY, SHENZHEN, CHINA
[*] IMLOUISCHAN@GMAIL.COM

Supplementary text

Considering data, we would like to underline the fact that our estimates of effectiveness are data-driven at the country population scales, yet, they are not deterministic estimates valid at the individual scale or any other scale of analysis in principle. Certainly some of the estimates are more affected by the poor surveillance system of some countries; this is another major reason, beyond disease tracking, for which surveillance is extremely important for evaluating interventions during and a posteriori an epidemic across different geographies.

Infection curves were reconstructed by the confirmation curves using the deconvolution method. The back-calculated incidence retrieves the timing of infection to match with the implementations in order to evaluate the corresponding impact on reducing cases. The reconstructed curve is sensitive to the initial condition that is one of the features of complex systems. Countries with persistent cyclical fluctuations (e.g., Albania, Bosnia and Herzegovina, Greece, Kazakhstan, Kosovo, Lithuania, Malaysia, Mauritius, Montenegro, and Romania) in reported data may manifest a systematic surveillance malfunction. Then we employed smooth infection curves that is the theoretical expectation about epidemic dynamics without extra stochastic forcing, including surveillance issues. The difference between the noisy curve (alternative setting) and the theoretical curve (baseline setting) gives an idea of the effect stochastic forcing and data-related issues.

Other features may cause variability in our estimation, however, these features mentioned below were not measurable because their information is not available. The chosen time delays were assessed from data of Japan and applied to other 49 countries. Realistically, the symptomatic-to-confirmation time delay (and not the incubation period) may differ among countries depending on their surveillance system but individual data are only

Date: April 5, 2021.

available for Japan, thus, this country is the only one for which the estimation can be carried out. A homogeneous symptomatic-to-confirmation time delay, however, allows one to test the difference among countries for the same average delay. As for the transmission dynamics parameters, we considered the reproduction number to be constant between implementations due to the lack of information about the duration of and time requested for implementation. Then, the effect of implementations was easily evaluated by comparing the daily updated estimates of R_t without the change in cases between implementations. Additionally, reproduction numbers (estimated using the EpiEsim package [1, 2]) are not taking into account asymptomatic cases which are unobserved. Additionally, all cases reported locally may have been imported cases (that is unavailable information) and yet, theoretically reproduction numbers can be overestimated. However, the purpose of this study is to assess at the country scale the effectiveness of interventions independently of fine details that actually may just rescale all inferred reproduction numbers, and yet being not influential for effectiveness. In a broader view, many fine scale details of the processes investigated are present but a reductionist speculation about what potentially is important cannot carried out. Rather, a holistic macroscopic analysis of the case patterns, such as the one performed, already quantifies the effect of all fine scale variables without attributing their individual importance also because these variables are highly interdependent and yet they act in concert.

Further work can pinpoint optimal portfolios in terms of sequence of interventions, inter-timing and duration of implementation that can be related to total case magnitude or more importantly to some specific socio-environmental conditions of the countries.

References

[1] Anne Cori, Neil M Ferguson, Christophe Fraser, and Simon Cauchemez. A new framework and software to estimate time-varying reproduction numbers during epidemics. *American journal of epidemiology*, 178(9):1505–1512, 2013.

[2] RN Thompson, JE Stockwin, RD van Gaalen, JA Polonsky, ZN Kamvar, PA Demarsh, E Dahlqwist, S Li, Eve Miguel, T Jombart, et al. Improved inference of time-varying reproduction numbers during infectious disease outbreaks. *Epidemics*, 29:100356, 2019.

Index	Country	ISO code	Data source	Total days	Misreported	Proportion (%)
1	Albania	AL	John Hopkins	91	0	0
2	Austria	AT	WHO	103	2	1.9
3	Belgium	BE	John Hopkins	125	0	0
4	Bosnia and Herzegovina	BA	John Hopkins	95	0	0
5	Brazil	BR	WHO	102	1	1
6	Canada	CA	John Hopkins	134	0	0
7	Croatia	HR	John Hopkins	104	0	0
8	Czechia	CZ	John Hopkins	99	0	0
9	Denmark	DK	John Hopkins	102	0	0
10	Ecuador	EC	WHO	99	4	4
11	El Salvador	SV	John Hopkins	81	0	0
12	Estonia	EE	John Hopkins	102	0	0
13	Finland	FI	John Hopkins	131	0	0
14	France	FR	WHO	134	3	2.2
15	Germany	DE	John Hopkins	133	0	0
16	Greece	GR	John Hopkins	103	0	0
17	Honduras	HN	WHO	88	2	2.3
18	Hungary	HU	John Hopkins	96	0	0
19	Iceland	IS	John Hopkins	101	0	0
20	India	IN	John Hopkins	130	0	0
21	Indonesia	ID	John Hopkins	98	0	0
22	Ireland	IE	John Hopkins	100	0	0
23	Italy	IT	John Hopkins	129	0	0
24	Japan	JP	Japan	145	0	0
25	Kazakhstan	KZ	WHO	85	1	1.2
26	Kosovo	XK	WHO	77	1	1.3
27	Kuwait	KW	John Hopkins	105	0	0
28	Lithuania	LT	John Hopkins	101	1	1
29	Malaysia	MY	John Hopkins	135	0	0
30	Mauritius	MU	John Hopkins	82	1	1.2
31	Mexico	MX	John Hopkins	101	0	0
32	Montenegro	ME	John Hopkins	83	0	0
33	Netherlands	NL	John Hopkins	102	0	0
34	New Zealand	NZ	John Hopkins	101	2	2
35	North Macedonia	MK	John Hopkins	103	0	0
36	Norway	NO	WHO	102	1	1
37	Portugal	PT	John Hopkins	98	1	1
38	Republic of Korea	KR	John Hopkins	138	0	0
39	Romania	RO	John Hopkins	103	0	0
40	Serbia	RS	WHO	94	1	1.1
41	Singapore	SG	John Hopkins	137	0	0
42	Slovakia	SK	John Hopkins	94	0	0
43	Slovenia	SI	John Hopkins	95	0	0
44	Spain	ES	John Hopkins	128	2	1.6
45	Sweden	SE	John Hopkins	129	0	0
46	Switzerland	CH	John Hopkins	104	0	0
47	Syrian Arab Republic	SY	John Hopkins	78	0	0
48	Thailand	TH	John Hopkins	138	0	0
49	The United Kingdom	GB	John Hopkins	129	1	0.8
50	United States of America	US	John Hopkins	138	0	0

TABLE S1. The misreported incidence data of 50 countries. The data were taken from a local dataset from Japan; or from WHO for 9 countries; and John Hopkins for 40 countries. The proportion of the total misreported time points was 0.4%.

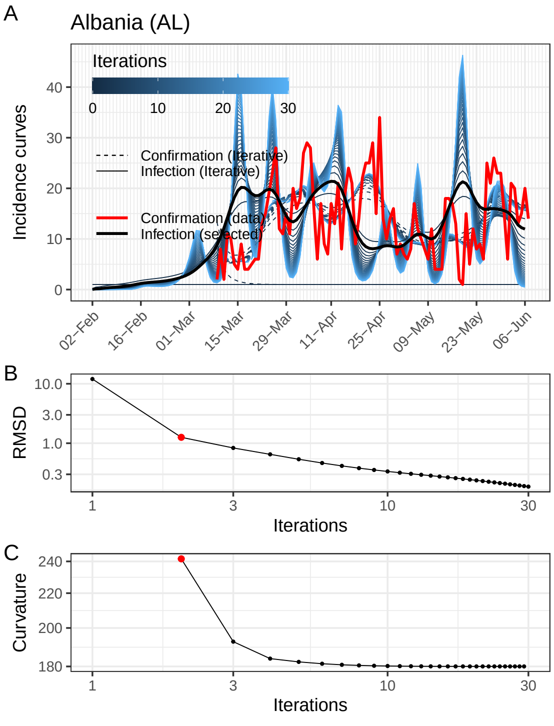

(A) **Deconvolution iterations using uninformative initial condition.** A. The RL deconvolution iterative curves and observed confirmation curve. B: The root-mean-square difference (RMSD) of all iterations. C: The curvature of RMSD. The red dot represents the selected iteration with maximum curvature.

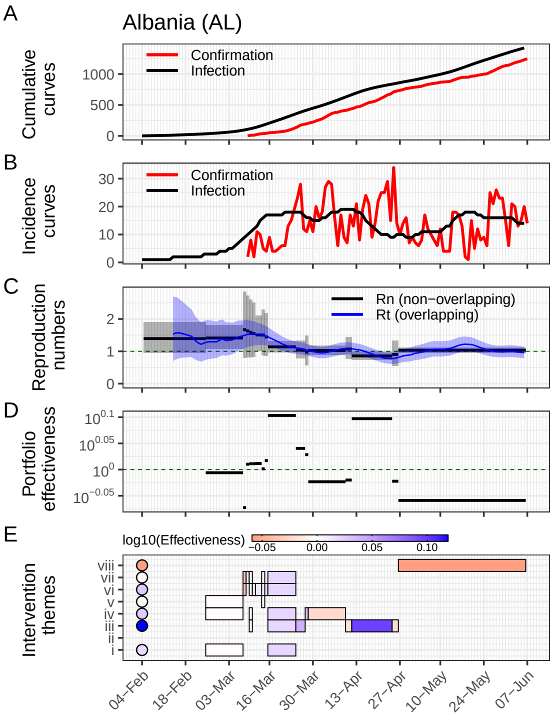

(B) **Estimates using uninformative initial condition.** A. The cumulative curves. B. The incidence curves. C. The instantaneous reproduction numbers R_n and R_t. D. The portfolio effectiveness η_n. E. The intervention $\eta_n^{(m)}$ (in rectangles) and aggregated $\eta^{(m)}$ (in circles) effectiveness.

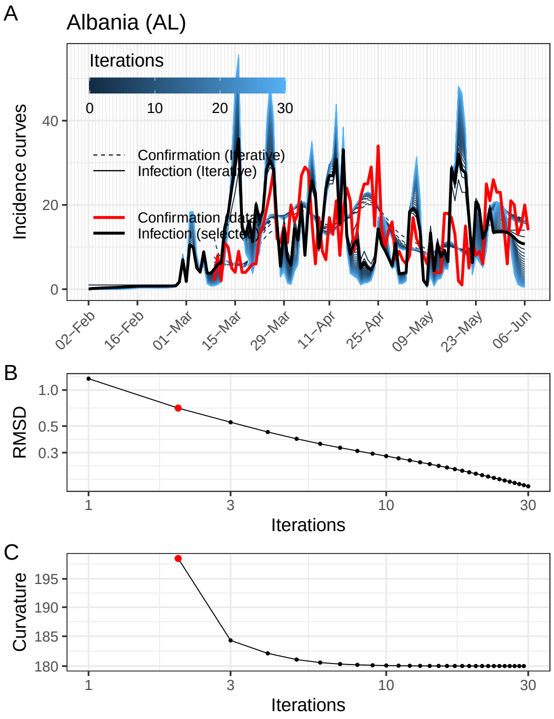

(C) **Deconvolution iterations using informative initial condition.** A. The RL deconvolution iterative curves and observed confirmation curve. B: The root-mean-square difference (RMSD) of all iterations. C: The curvature of RMSD. The red dot represents the selected iteration with maximum curvature.

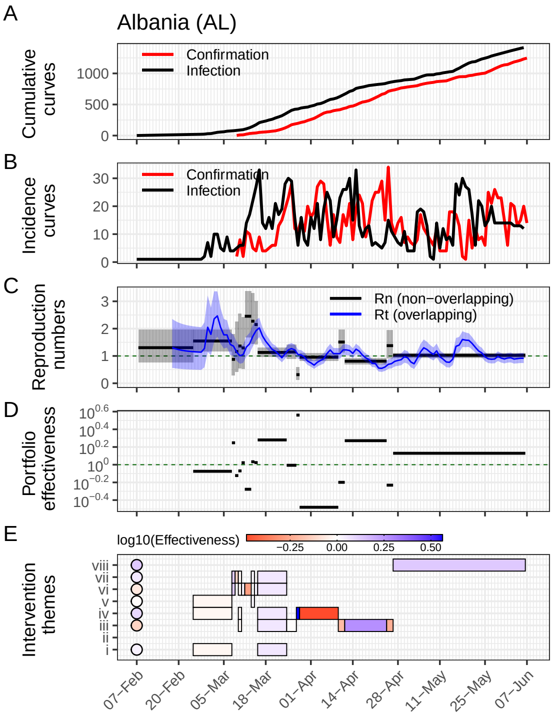

(D) **Estimates using informative initial condition.** A. The cumulative curves. B. The incidence curves. C. The instantaneous reproduction numbers R_n and R_t. D. The portfolio effectiveness η_n. E. The intervention $\eta_n^{(m)}$ (in rectangles) and aggregated $\eta^{(m)}$ (in circles) effectiveness.

Figure S1. **The reconstructed epidemic curves and subsequent evaluation of interventions implemented in Albania.** Estimation using uninformative (baseline setting, A and B) and informative (alternative setting, C and D) initial condition.

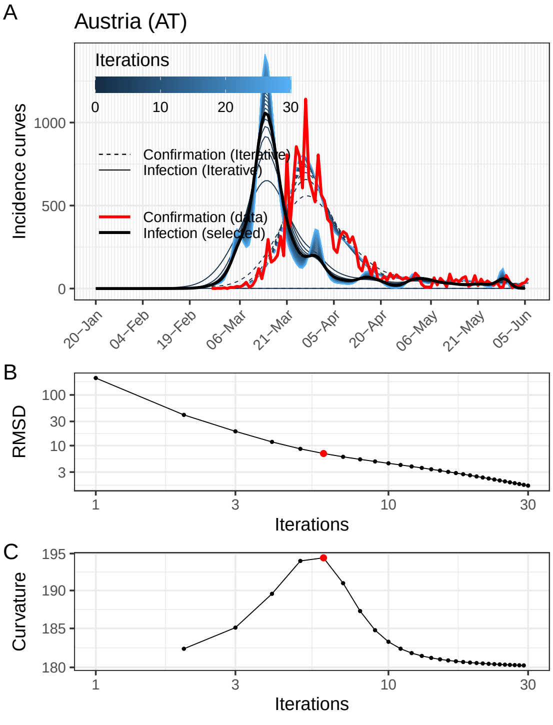

(A) **Deconvolution iterations using uninformative initial condition.** A. The RL deconvolution iterative curves and observed confirmation curve. B: The root-mean-square difference (RMSD) of all iterations. C: The curvature of RMSD. The red dot represents the selected iteration with maximum curvature.

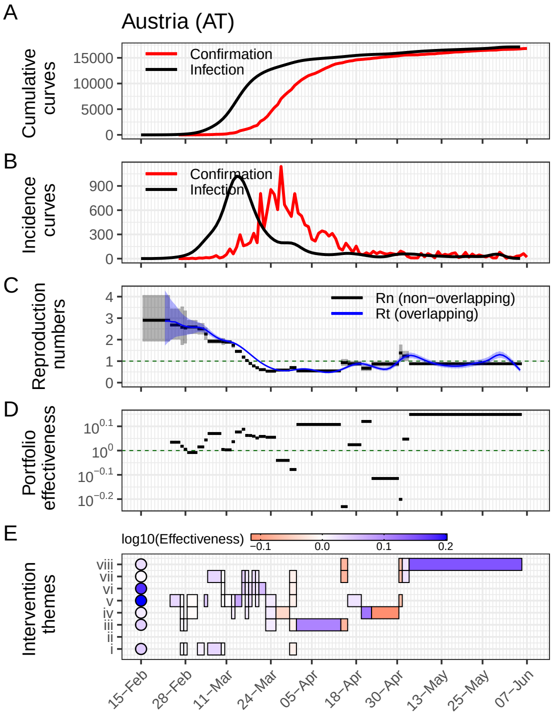

(B) **Estimates using uninformative initial condition.** A. The cumulative curves. B. The incidence curves. C. The instantaneous reproduction numbers R_n and R_t. D. The portfolio effectiveness η_n. E. The intervention $\eta_n^{(m)}$ (in rectangles) and aggregated $\eta^{(m)}$ (in circles) effectiveness.

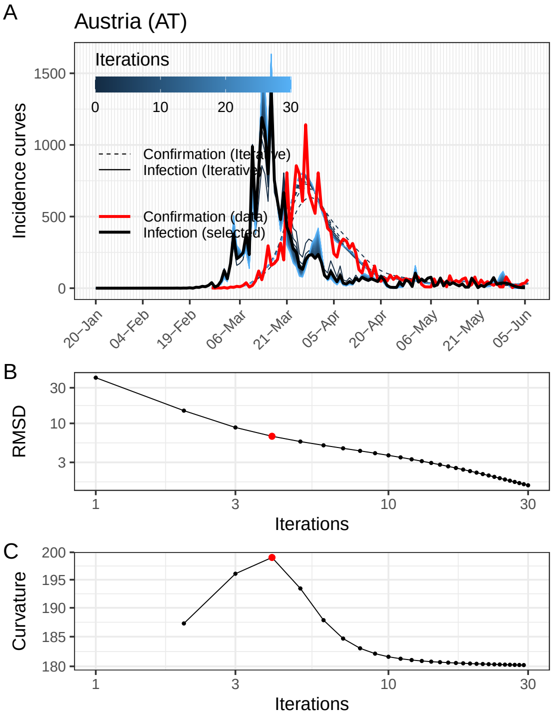

(C) **Deconvolution iterations using informative initial condition.** A. The RL deconvolution iterative curves and observed confirmation curve. B: The root-mean-square difference (RMSD) of all iterations. C: The curvature of RMSD. The red dot represents the selected iteration with maximum curvature.

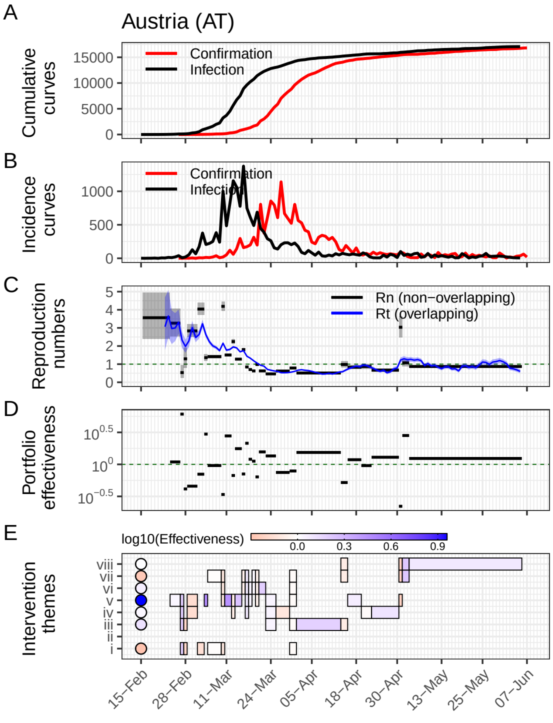

(D) **Estimates using informative initial condition.** A. The cumulative curves. B. The incidence curves. C. The instantaneous reproduction numbers R_n and R_t. D. The portfolio effectiveness η_n. E. The intervention $\eta_n^{(m)}$ (in rectangles) and aggregated $\eta^{(m)}$ (in circles) effectiveness.

FIGURE S2. **The reconstructed epidemic curves and subsequent evaluation of interventions implemented in Austria.** Estimation using uninformative (baseline setting, A and B) and informative (alternative setting, C and D) initial condition.

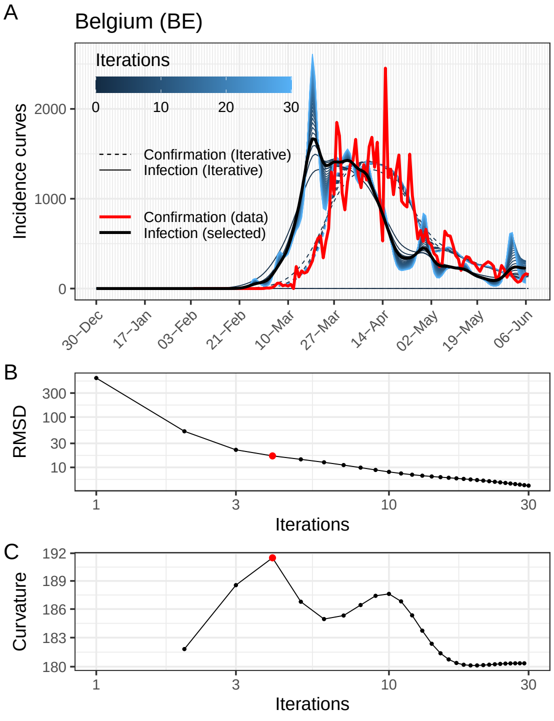

(A) **Deconvolution iterations using uninformative initial condition.** A. The RL deconvolution iterative curves and observed confirmation curve. B: The root-mean-square difference (RMSD) of all iterations. C: The curvature of RMSD. The red dot represents the selected iteration with maximum curvature.

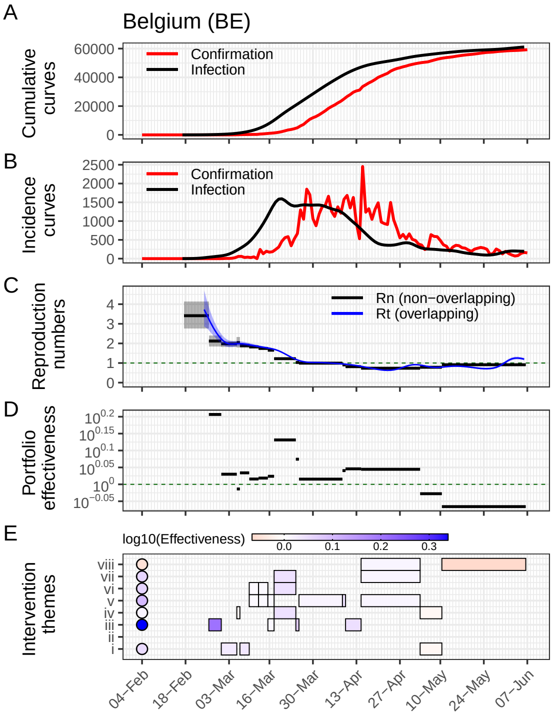

(B) **Estimates using uninformative initial condition.** A. The cumulative curves. B. The incidence curves. C. The instantaneous reproduction numbers R_n and R_t. D. The portfolio effectiveness η_n. E. The intervention $\eta_n^{(m)}$ (in rectangles) and aggregated $\eta^{(m)}$ (in circles) effectiveness.

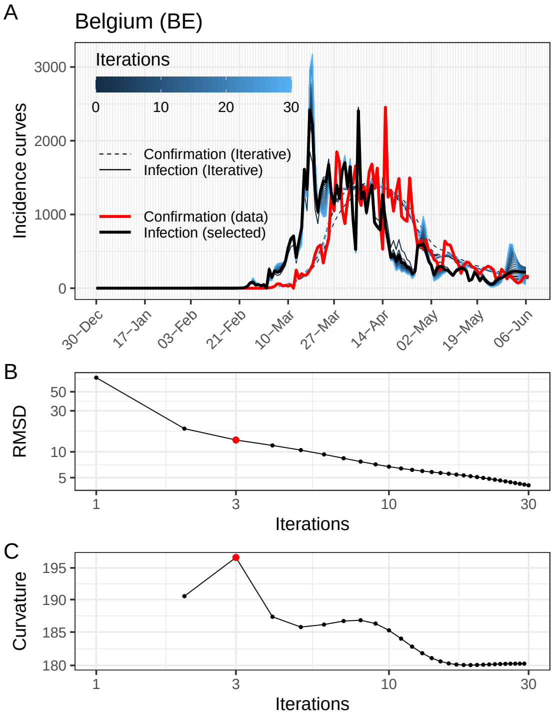

(C) **Deconvolution iterations using informative initial condition.** A. The RL deconvolution iterative curves and observed confirmation curve. B: The root-mean-square difference (RMSD) of all iterations. C: The curvature of RMSD. The red dot represents the selected iteration with maximum curvature.

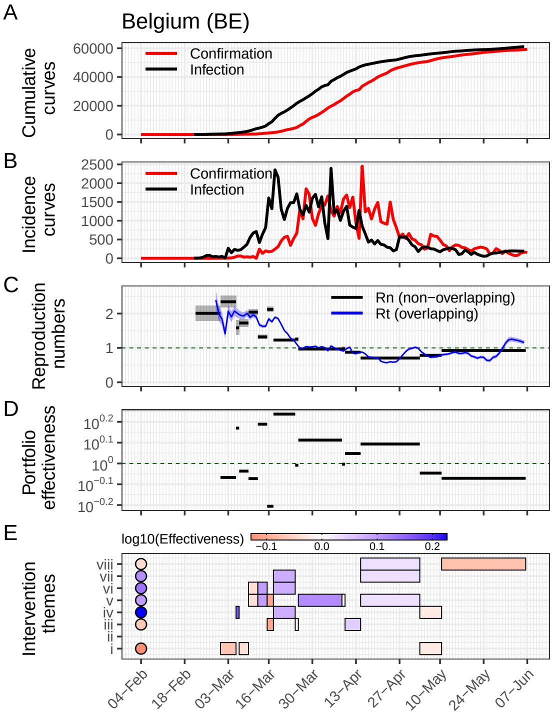

(D) **Estimates using informative initial condition.** A. The cumulative curves. B. The incidence curves. C. The instantaneous reproduction numbers R_n and R_t. D. The portfolio effectiveness η_n. E. The intervention $\eta_n^{(m)}$ (in rectangles) and aggregated $\eta^{(m)}$ (in circles) effectiveness.

FIGURE S3. **The reconstructed epidemic curves and subsequent evaluation of interventions implemented in Belgium.** Estimation using uninformative (baseline setting, A and B) and informative (alternative setting, C and D) initial condition.

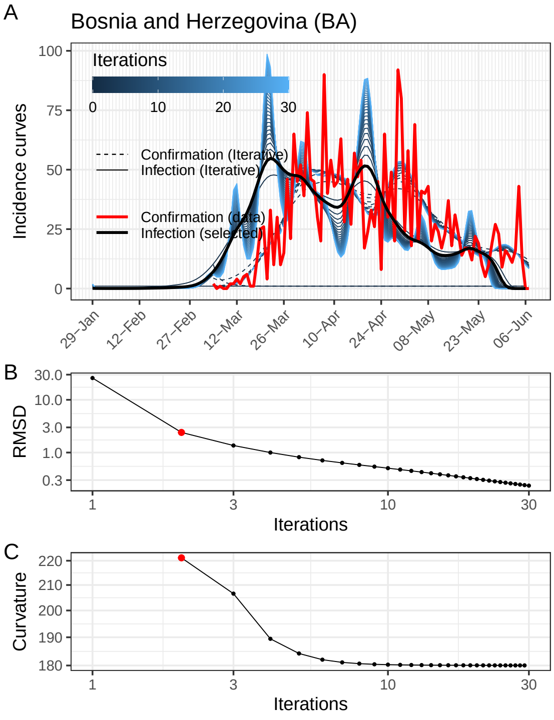

(A) **Deconvolution iterations using uninformative initial condition.** A. The RL deconvolution iterative curves and observed confirmation curve. B: The root-mean-square difference (RMSD) of all iterations. C: The curvature of RMSD. The red dot represents the selected iteration with maximum curvature.

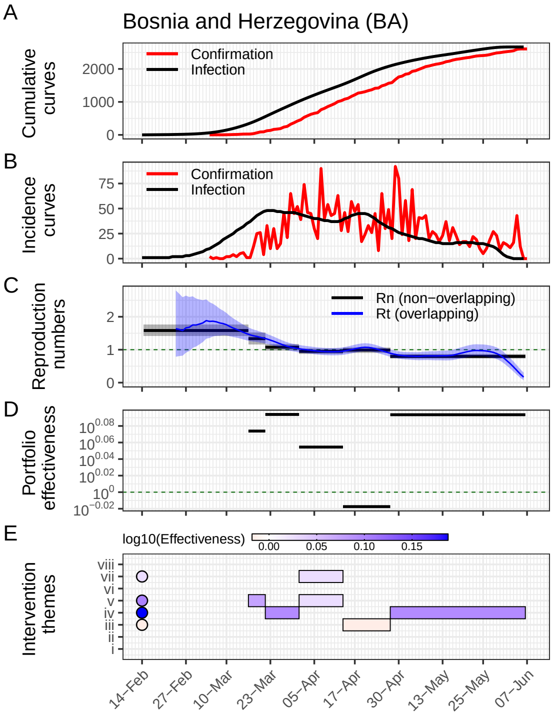

(B) **Estimates using uninformative initial condition.** A. The cumulative curves. B. The incidence curves. C. The instantaneous reproduction numbers R_n and R_t. D. The portfolio effectiveness η_n. E. The intervention $\eta_n^{(m)}$ (in rectangles) and aggregated $\eta^{(m)}$ (in circles) effectiveness.

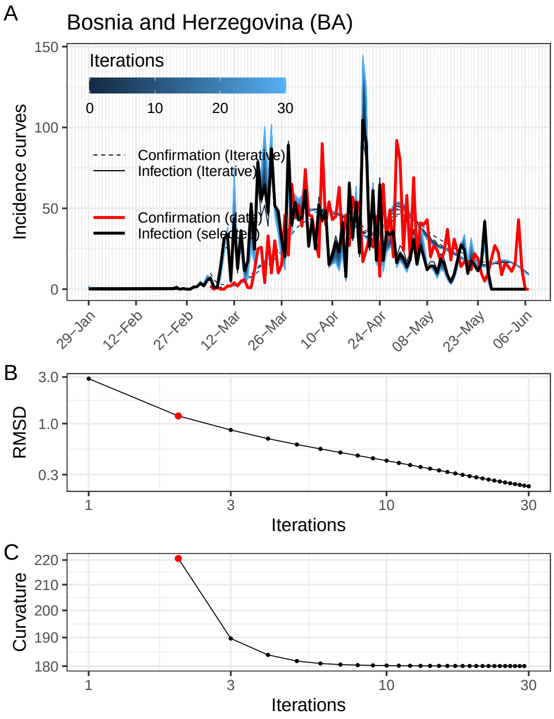

(C) **Deconvolution iterations using informative initial condition.** A. The RL deconvolution iterative curves and observed confirmation curve. B: The root-mean-square difference (RMSD) of all iterations. C: The curvature of RMSD. The red dot represents the selected iteration with maximum curvature.

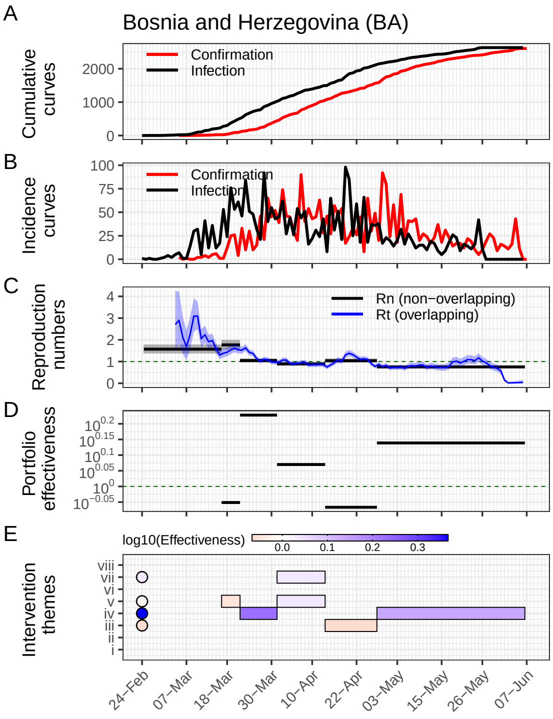

(D) **Estimates using informative initial condition.** A. The cumulative curves. B. The incidence curves. C. The instantaneous reproduction numbers R_n and R_t. D. The portfolio effectiveness η_n. E. The intervention $\eta_n^{(m)}$ (in rectangles) and aggregated $\eta^{(m)}$ (in circles) effectiveness.

FIGURE S4. **The reconstructed epidemic curves and subsequent evaluation of interventions implemented in Bosnia and Herzegovina.** Estimation using uninformative (baseline setting, A and B) and informative (alternative setting, C and D) initial condition.

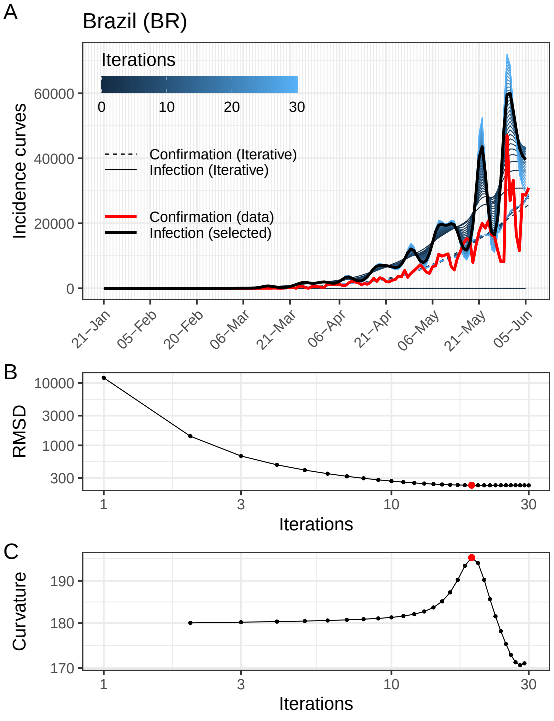

(A) **Deconvolution iterations using uninformative initial condition.** A. The RL deconvolution iterative curves and observed confirmation curve. B: The root-mean-square difference (RMSD) of all iterations. C: The curvature of RMSD. The red dot represents the selected iteration with maximum curvature.

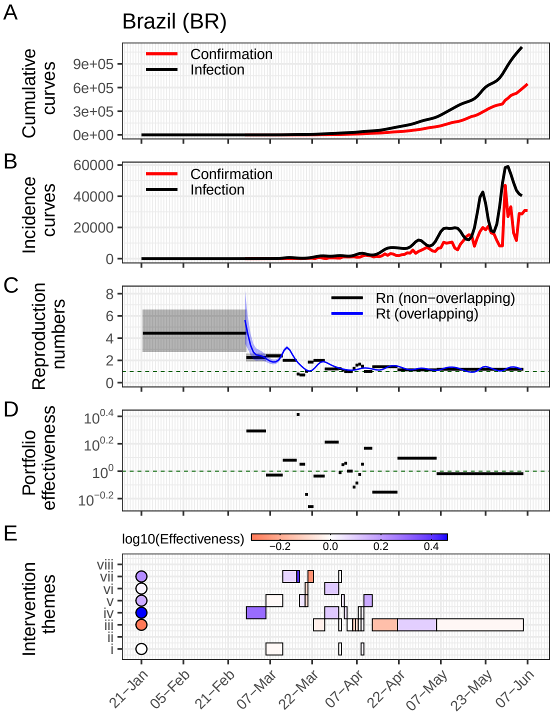

(B) **Estimates using uninformative initial condition.** A. The cumulative curves. B. The incidence curves. C. The instantaneous reproduction numbers R_n and R_t. D. The portfolio effectiveness η_n. E. The intervention $\eta_n^{(m)}$ (in rectangles) and aggregated $\eta^{(m)}$ (in circles) effectiveness.

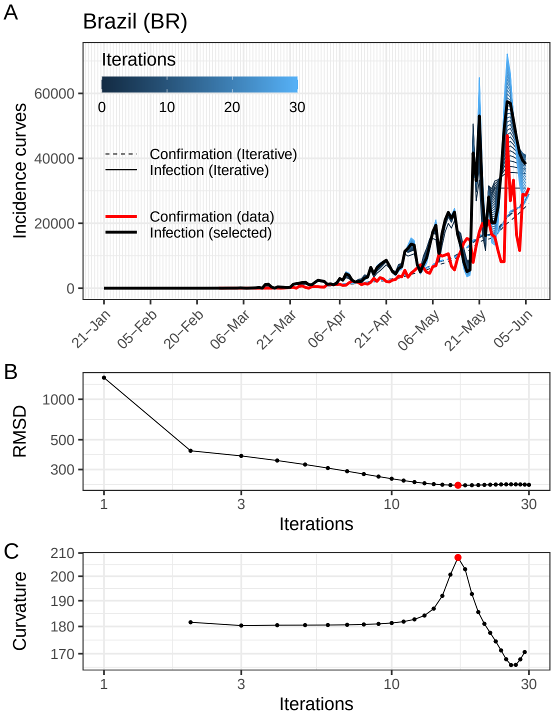

(C) **Deconvolution iterations using informative initial condition.** A. The RL deconvolution iterative curves and observed confirmation curve. B: The root-mean-square difference (RMSD) of all iterations. C: The curvature of RMSD. The red dot represents the selected iteration with maximum curvature.

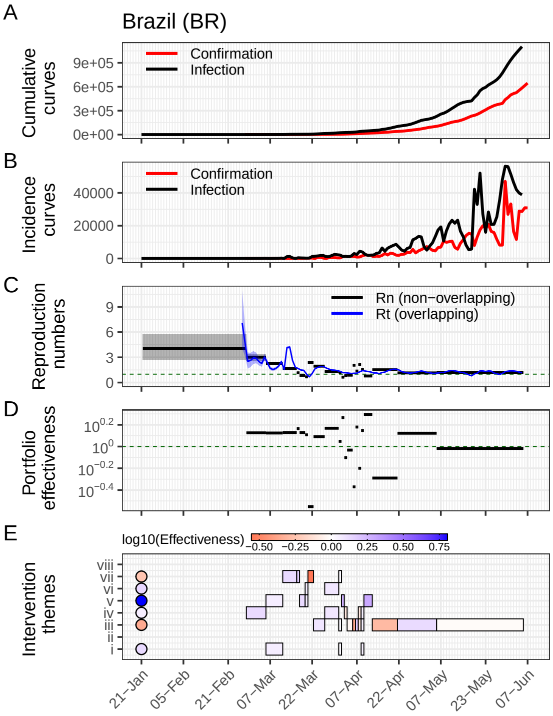

(D) **Estimates using informative initial condition.** A. The cumulative curves. B. The incidence curves. C. The instantaneous reproduction numbers R_n and R_t. D. The portfolio effectiveness η_n. E. The intervention $\eta_n^{(m)}$ (in rectangles) and aggregated $\eta^{(m)}$ (in circles) effectiveness.

FIGURE S5. **The reconstructed epidemic curves and subsequent evaluation of interventions implemented in Brazil.** Estimation using uninformative (baseline setting, A and B) and informative (alternative setting, C and D) initial condition.

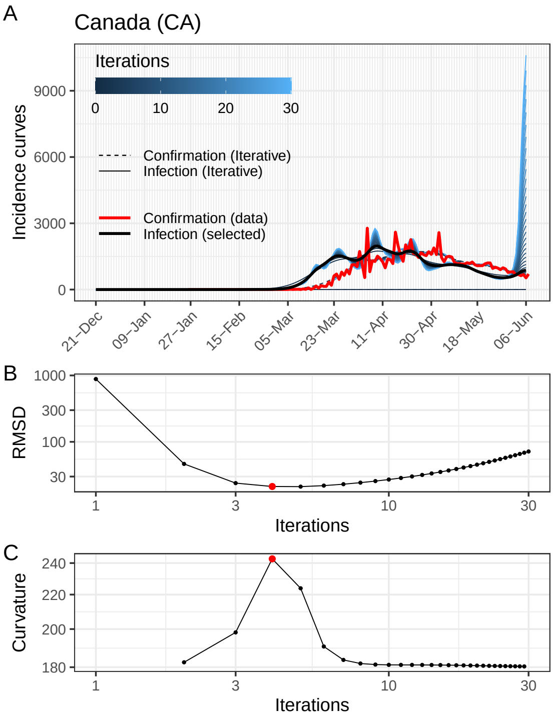

(A) **Deconvolution iterations using uninformative initial condition.** A. The RL deconvolution iterative curves and observed confirmation curve. B: The root-mean-square difference (RMSD) of all iterations. C: The curvature of RMSD. The red dot represents the selected iteration with maximum curvature.

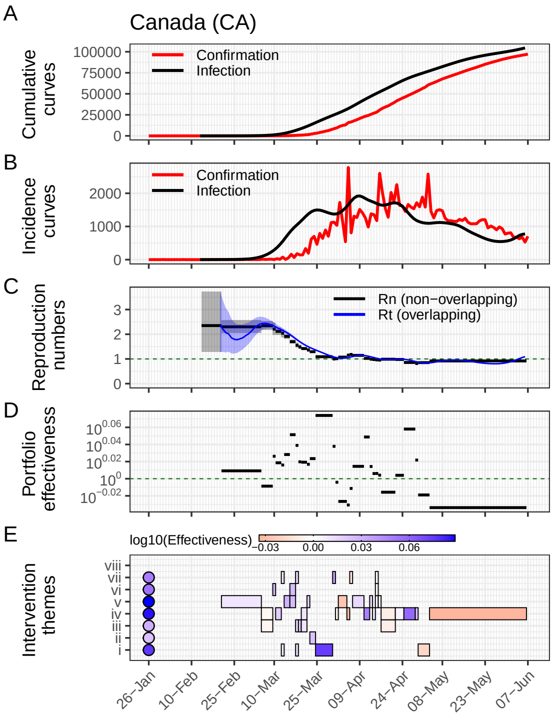

(B) **Estimates using uninformative initial condition.** A. The cumulative curves. B. The incidence curves. C. The instantaneous reproduction numbers R_n and R_t. D. The portfolio effectiveness η_n. E. The intervention $\eta_n^{(m)}$ (in rectangles) and aggregated $\eta^{(m)}$ (in circles) effectiveness.

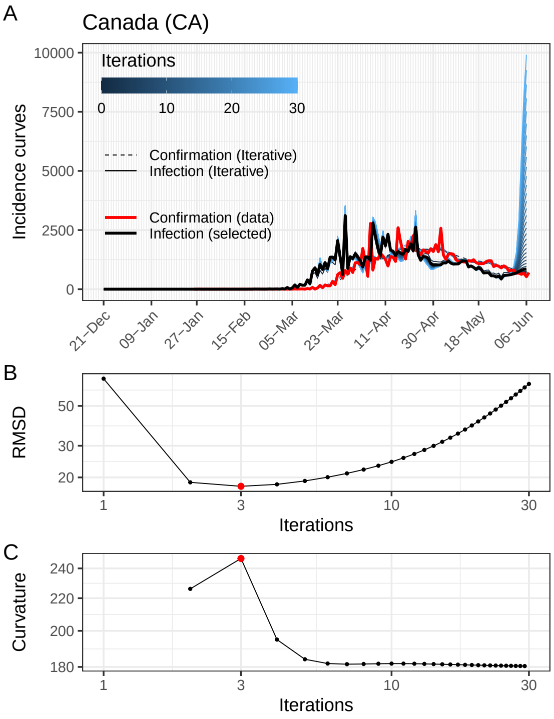

(C) **Deconvolution iterations using informative initial condition.** A. The RL deconvolution iterative curves and observed confirmation curve. B: The root-mean-square difference (RMSD) of all iterations. C: The curvature of RMSD. The red dot represents the selected iteration with maximum curvature.

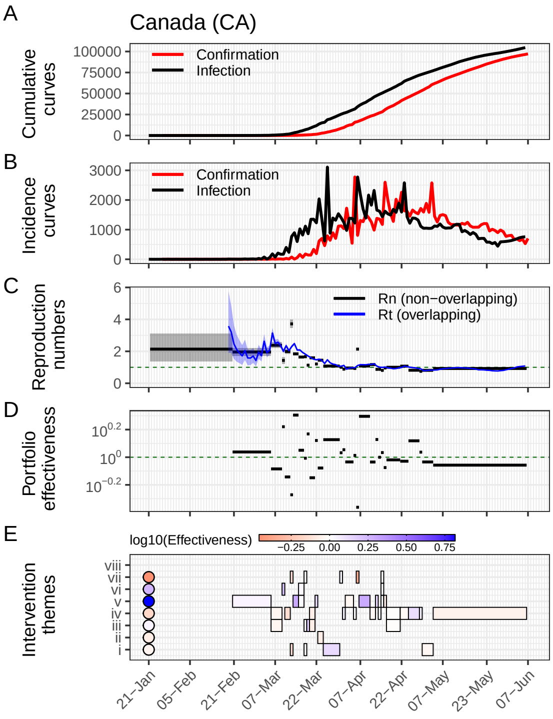

(D) **Estimates using informative initial condition.** A. The cumulative curves. B. The incidence curves. C. The instantaneous reproduction numbers R_n and R_t. D. The portfolio effectiveness η_n. E. The intervention $\eta_n^{(m)}$ (in rectangles) and aggregated $\eta^{(m)}$ (in circles) effectiveness.

Figure S6. **The reconstructed epidemic curves and subsequent evaluation of interventions implemented in Canada.** Estimation using uninformative (baseline setting, A and B) and informative (alternative setting, C and D) initial condition.

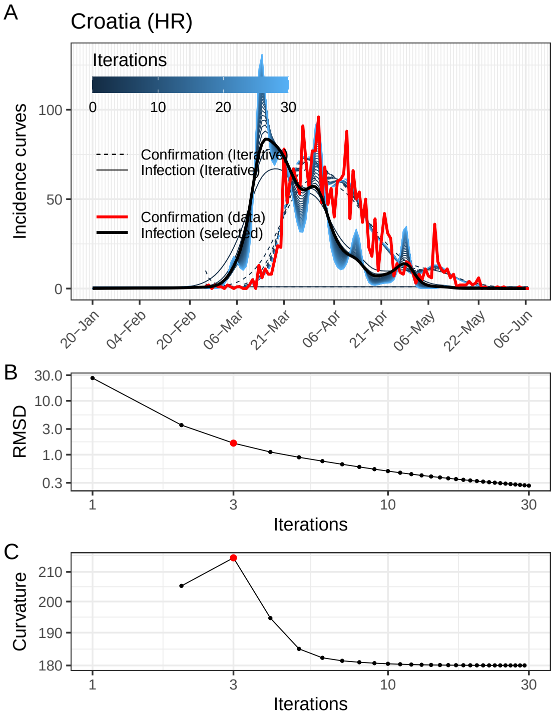

(A) **Deconvolution iterations using uninformative initial condition.** A. The RL deconvolution iterative curves and observed confirmation curve. B: The root-mean-square difference (RMSD) of all iterations. C: The curvature of RMSD. The red dot represents the selected iteration with maximum curvature.

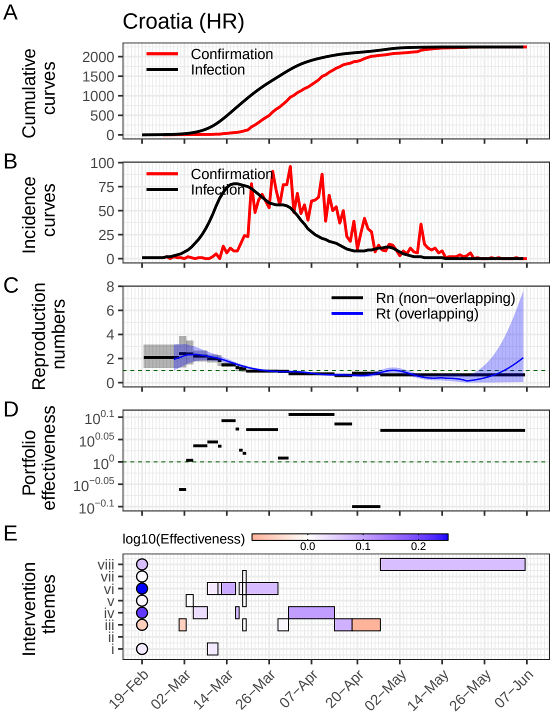

(B) **Estimates using uninformative initial condition.** A. The cumulative curves. B. The incidence curves. C. The instantaneous reproduction numbers R_n and R_t. D. The portfolio effectiveness η_n. E. The intervention $\eta_n^{(m)}$ (in rectangles) and aggregated $\eta^{(m)}$ (in circles) effectiveness.

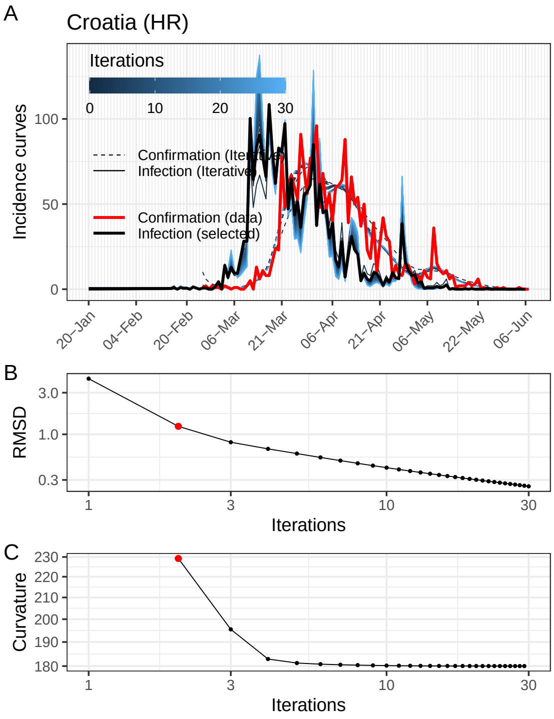

(C) **Deconvolution iterations using informative initial condition.** A. The RL deconvolution iterative curves and observed confirmation curve. B: The root-mean-square difference (RMSD) of all iterations. C: The curvature of RMSD. The red dot represents the selected iteration with maximum curvature.

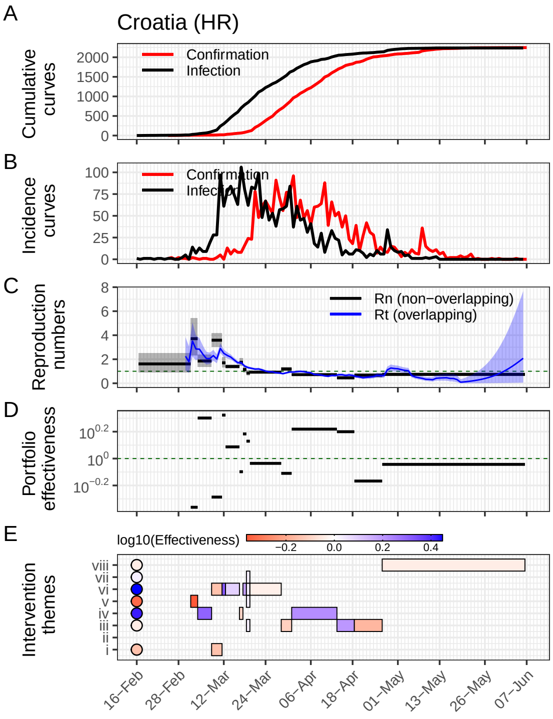

(D) **Estimates using informative initial condition.** A. The cumulative curves. B. The incidence curves. C. The instantaneous reproduction numbers R_n and R_t. D. The portfolio effectiveness η_n. E. The intervention $\eta_n^{(m)}$ (in rectangles) and aggregated $\eta^{(m)}$ (in circles) effectiveness.

FIGURE S7. **The reconstructed epidemic curves and subsequent evaluation of interventions implemented in Croatia.** Estimation using uninformative (baseline setting, A and B) and informative (alternative setting, C and D) initial condition.

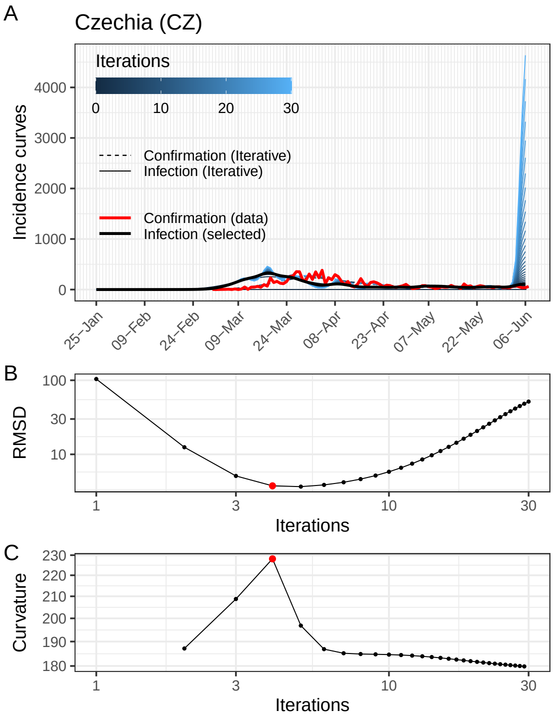

(A) **Deconvolution iterations using uninformative initial condition.** A. The RL deconvolution iterative curves and observed confirmation curve. B: The root-mean-square difference (RMSD) of all iterations. C: The curvature of RMSD. The red dot represents the selected iteration with maximum curvature.

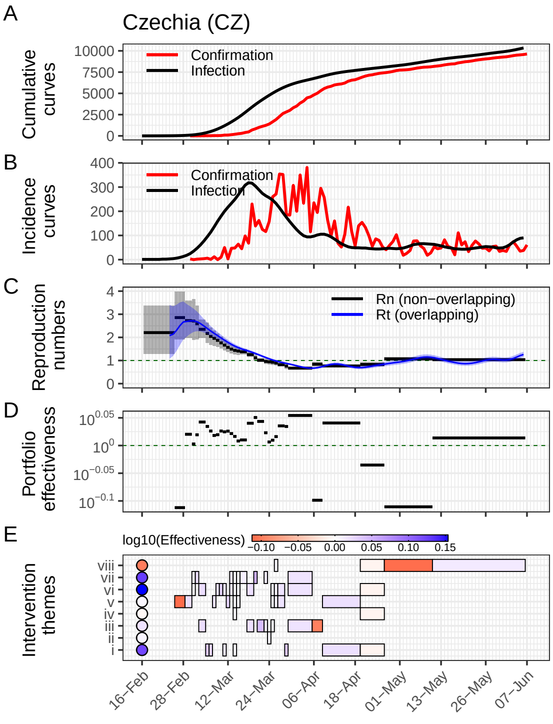

(B) **Estimates using uninformative initial condition.** A. The cumulative curves. B. The incidence curves. C. The instantaneous reproduction numbers R_n and R_t. D. The portfolio effectiveness η_n. E. The intervention $\eta_n^{(m)}$ (in rectangles) and aggregated $\eta^{(m)}$ (in circles) effectiveness.

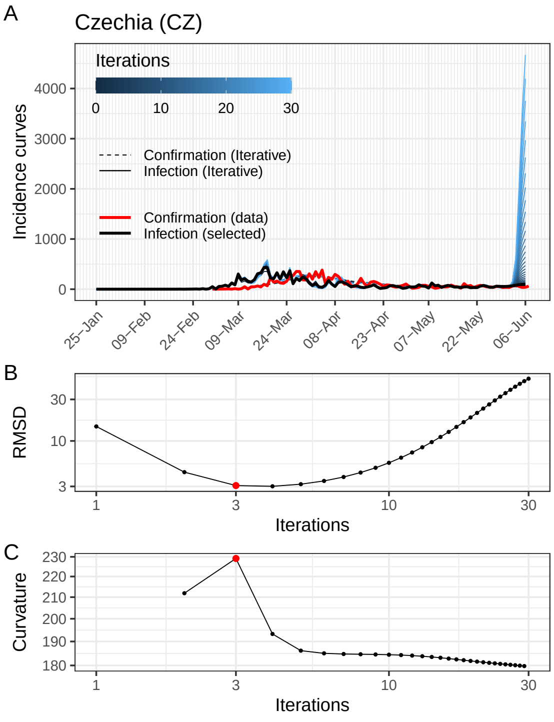

(C) **Deconvolution iterations using informative initial condition.** A. The RL deconvolution iterative curves and observed confirmation curve. B: The root-mean-square difference (RMSD) of all iterations. C: The curvature of RMSD. The red dot represents the selected iteration with maximum curvature.

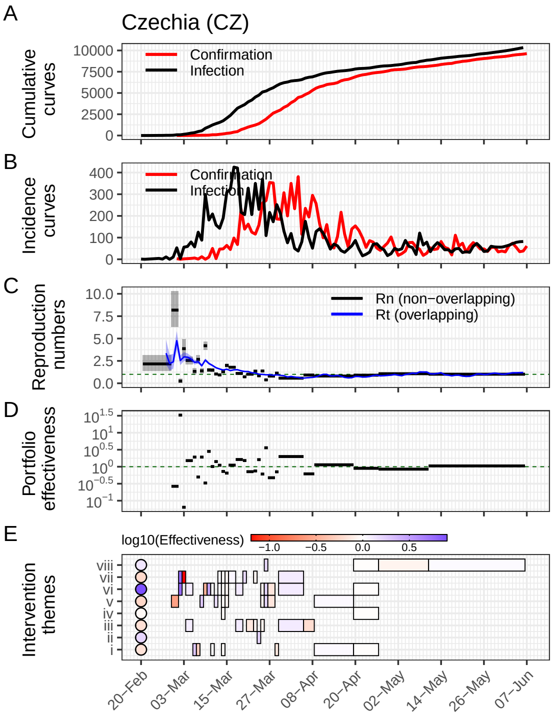

(D) **Estimates using informative initial condition.** A. The cumulative curves. B. The incidence curves. C. The instantaneous reproduction numbers R_n and R_t. D. The portfolio effectiveness η_n. E. The intervention $\eta_n^{(m)}$ (in rectangles) and aggregated $\eta^{(m)}$ (in circles) effectiveness.

FIGURE S8. **The reconstructed epidemic curves and subsequent evaluation of interventions implemented in Czechia.** Estimation using uninformative (baseline setting, A and B) and informative (alternative setting, C and D) initial condition.

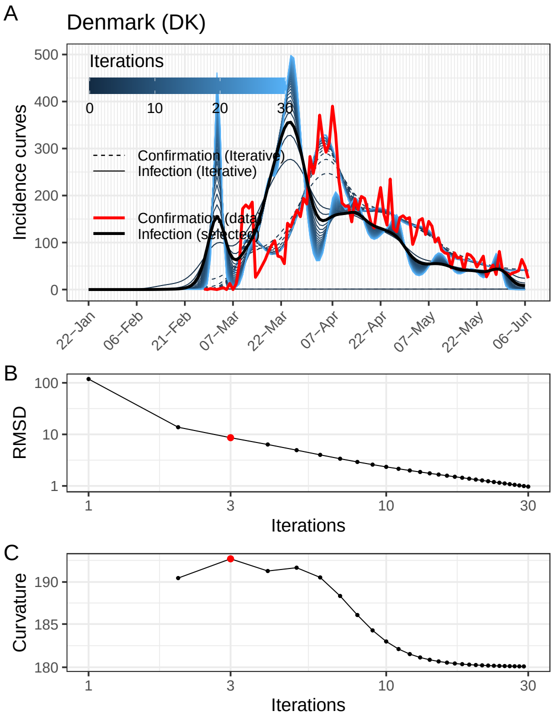

(A) **Deconvolution iterations using uninformative initial condition.** A. The RL deconvolution iterative curves and observed confirmation curve. B: The root-mean-square difference (RMSD) of all iterations. C: The curvature of RMSD. The red dot represents the selected iteration with maximum curvature.

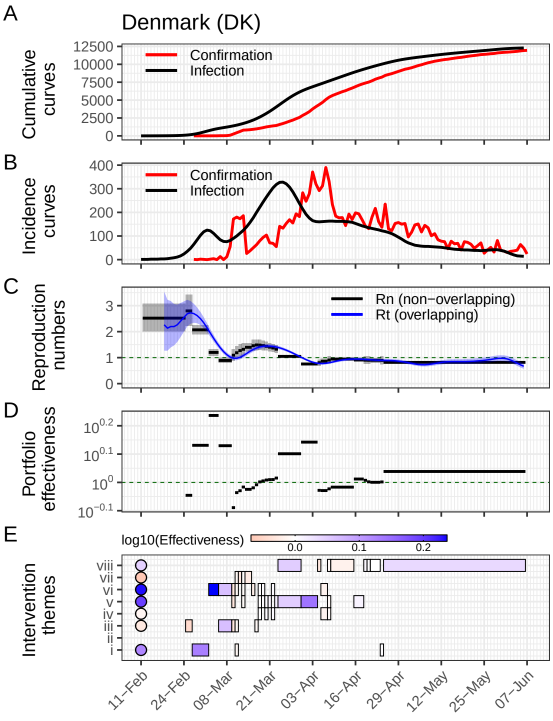

(B) **Estimates using uninformative initial condition.** A. The cumulative curves. B. The incidence curves. C. The instantaneous reproduction numbers R_n and R_t. D. The portfolio effectiveness η_n. E. The intervention $\eta_n^{(m)}$ (in rectangles) and aggregated $\eta^{(m)}$ (in circles) effectiveness.

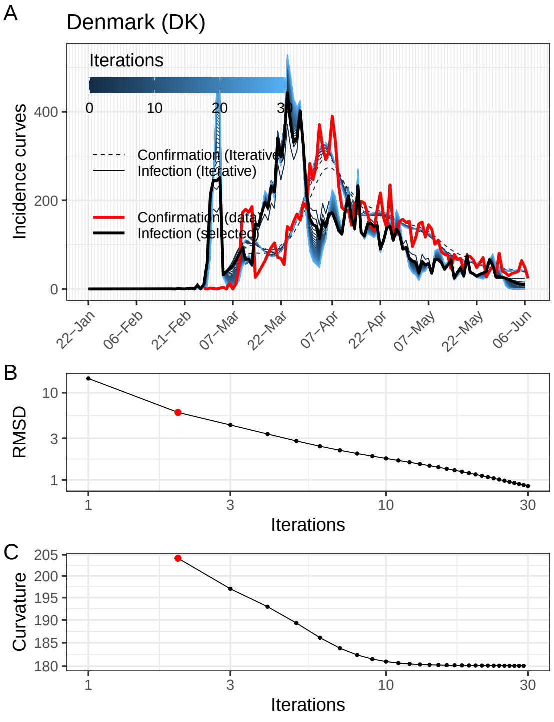

(C) **Deconvolution iterations using informative initial condition.** A. The RL deconvolution iterative curves and observed confirmation curve. B: The root-mean-square difference (RMSD) of all iterations. C: The curvature of RMSD. The red dot represents the selected iteration with maximum curvature.

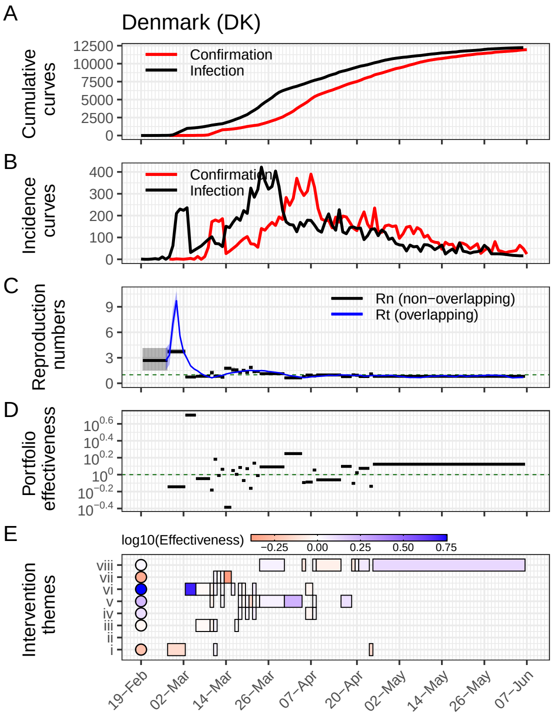

(D) **Estimates using informative initial condition.** A. The cumulative curves. B. The incidence curves. C. The instantaneous reproduction numbers R_n and R_t. D. The portfolio effectiveness η_n. E. The intervention $\eta_n^{(m)}$ (in rectangles) and aggregated $\eta^{(m)}$ (in circles) effectiveness.

Figure S9. **The reconstructed epidemic curves and subsequent evaluation of interventions implemented in Denmark.** Estimation using uninformative (baseline setting, A and B) and informative (alternative setting, C and D) initial condition.

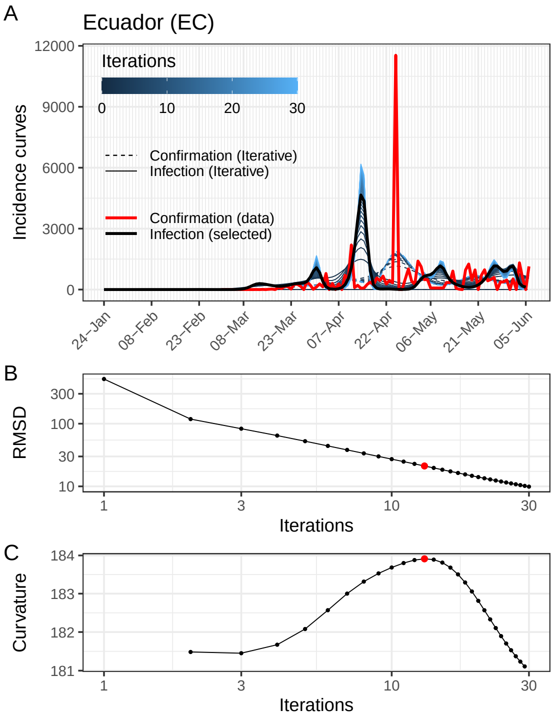

(A) **Deconvolution iterations using uninformative initial condition.** A. The RL deconvolution iterative curves and observed confirmation curve. B: The root-mean-square difference (RMSD) of all iterations. C: The curvature of RMSD. The red dot represents the selected iteration with maximum curvature.

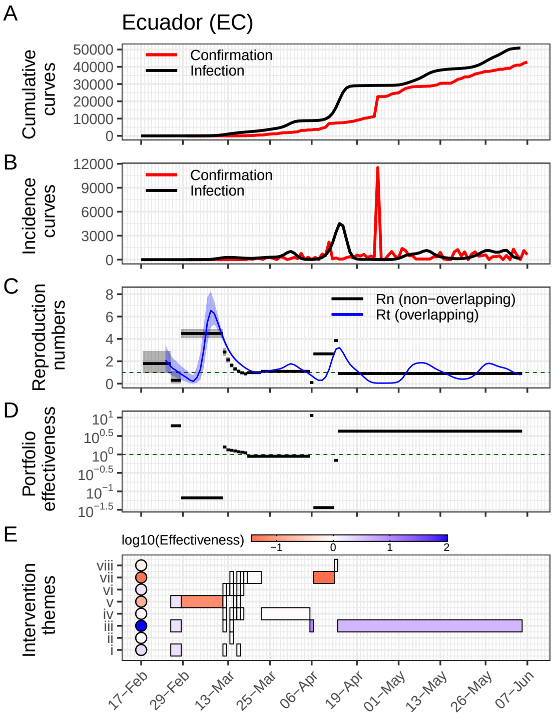

(B) **Estimates using uninformative initial condition.** A. The cumulative curves. B. The incidence curves. C. The instantaneous reproduction numbers R_n and R_t. D. The portfolio effectiveness η_n. E. The intervention $\eta_n^{(m)}$ (in rectangles) and aggregated $\eta^{(m)}$ (in circles) effectiveness.

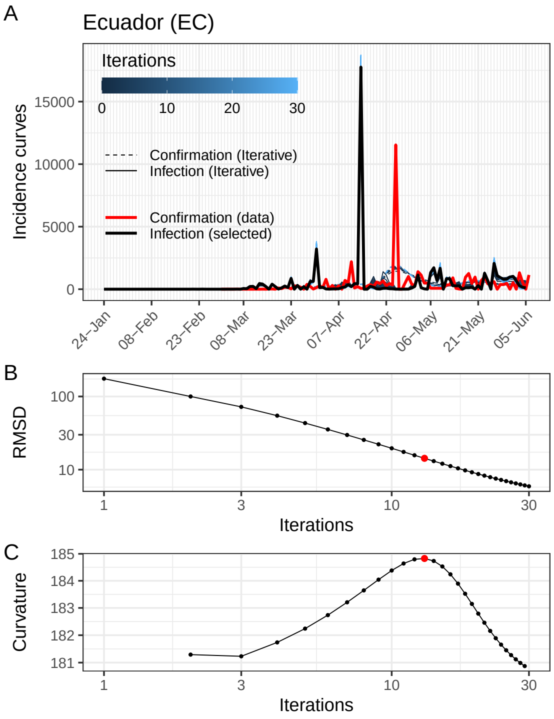

(C) **Deconvolution iterations using informative initial condition.** A. The RL deconvolution iterative curves and observed confirmation curve. B: The root-mean-square difference (RMSD) of all iterations. C: The curvature of RMSD. The red dot represents the selected iteration with maximum curvature.

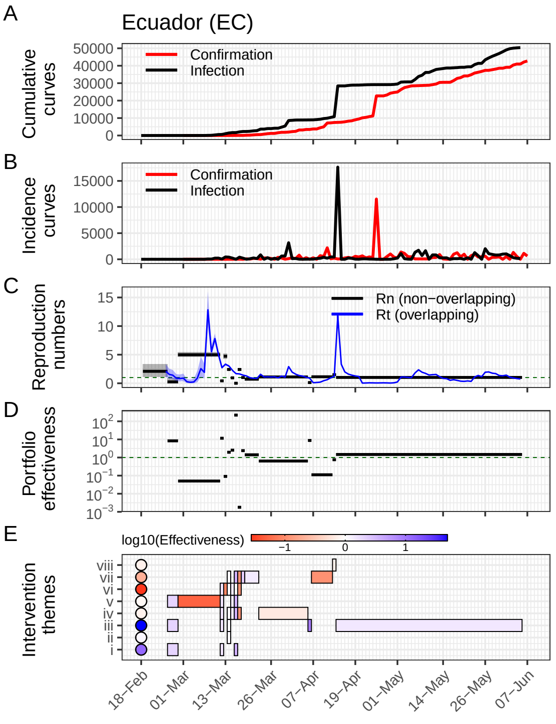

(D) **Estimates using informative initial condition.** A. The cumulative curves. B. The incidence curves. C. The instantaneous reproduction numbers R_n and R_t. D. The portfolio effectiveness η_n. E. The intervention $\eta_n^{(m)}$ (in rectangles) and aggregated $\eta^{(m)}$ (in circles) effectiveness.

FIGURE S10. **The reconstructed epidemic curves and subsequent evaluation of interventions implemented in Ecuador.** Estimation using uninformative (baseline setting, A and B) and informative (alternative setting, C and D) initial condition.

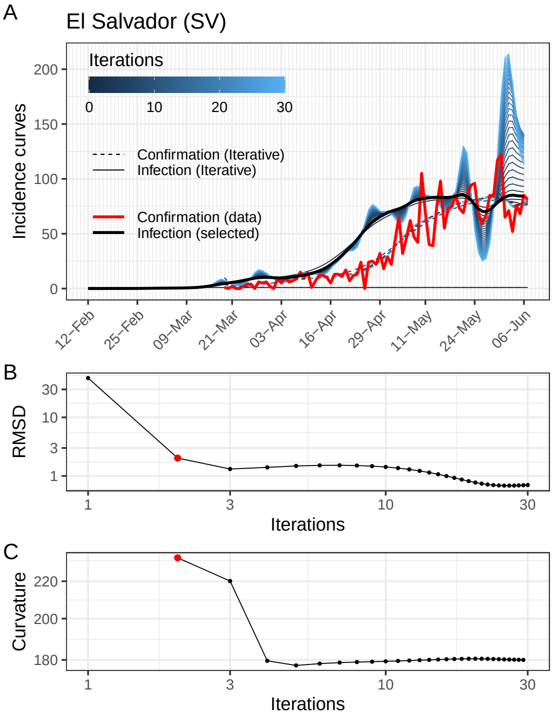

(A) **Deconvolution iterations using uninformative initial condition.** A. The RL deconvolution iterative curves and observed confirmation curve. B: The root-mean-square difference (RMSD) of all iterations. C: The curvature of RMSD. The red dot represents the selected iteration with maximum curvature.

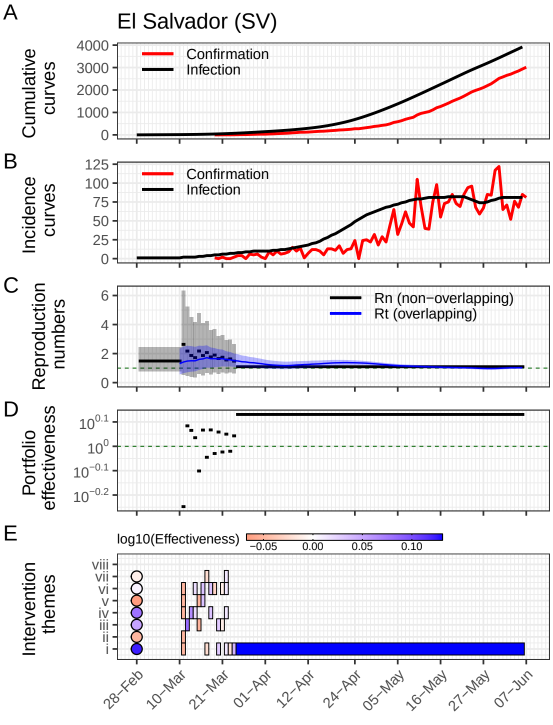

(B) **Estimates using uninformative initial condition.** A. The cumulative curves. B. The incidence curves. C. The instantaneous reproduction numbers R_n and R_t. D. The portfolio effectiveness η_n. E. The intervention $\eta_n^{(m)}$ (in rectangles) and aggregated $\eta^{(m)}$ (in circles) effectiveness.

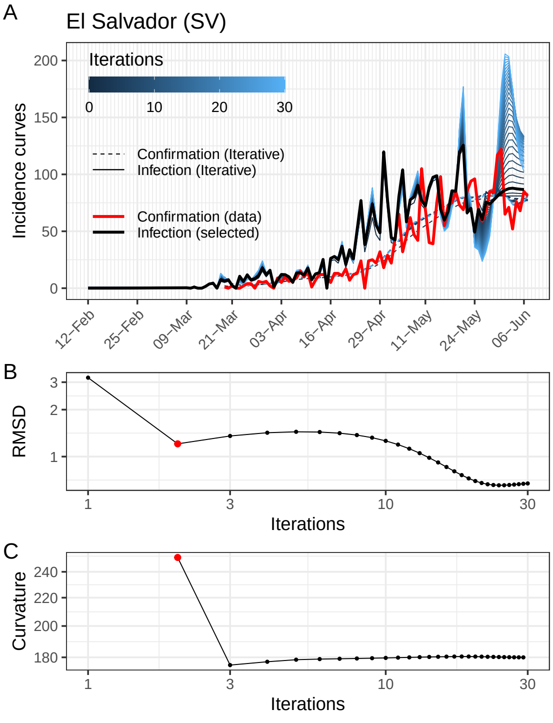

(C) **Deconvolution iterations using informative initial condition.** A. The RL deconvolution iterative curves and observed confirmation curve. B: The root-mean-square difference (RMSD) of all iterations. C: The curvature of RMSD. The red dot represents the selected iteration with maximum curvature.

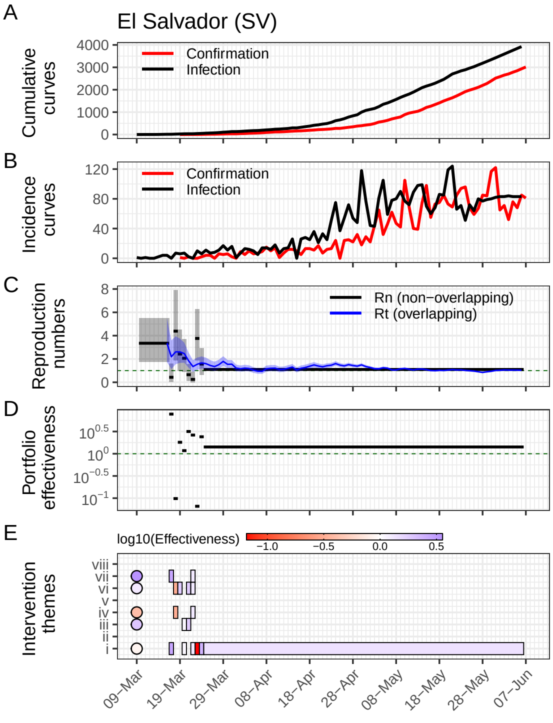

(D) **Estimates using informative initial condition.** A. The cumulative curves. B. The incidence curves. C. The instantaneous reproduction numbers R_n and R_t. D. The portfolio effectiveness η_n. E. The intervention $\eta_n^{(m)}$ (in rectangles) and aggregated $\eta^{(m)}$ (in circles) effectiveness.

FIGURE S11. **The reconstructed epidemic curves and subsequent evaluation of interventions implemented in El Salvador.** Estimation using uninformative (baseline setting, A and B) and informative (alternative setting, C and D) initial condition.

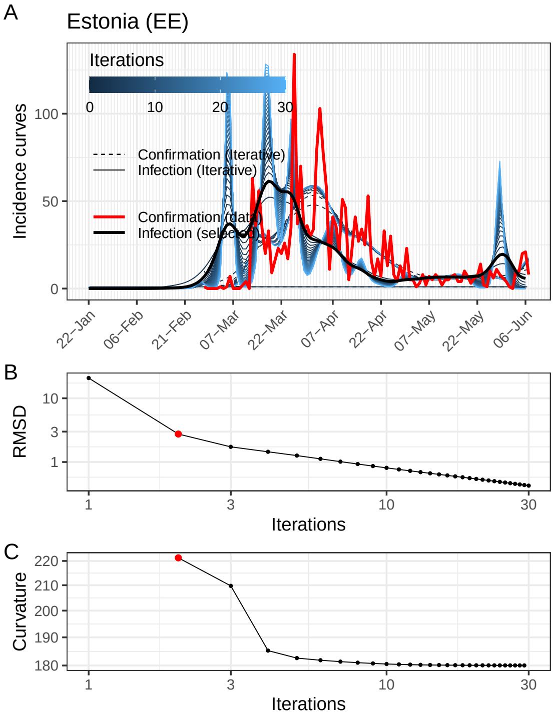

(A) **Deconvolution iterations using uninformative initial condition.** A. The RL deconvolution iterative curves and observed confirmation curve. B: The root-mean-square difference (RMSD) of all iterations. C: The curvature of RMSD. The red dot represents the selected iteration with maximum curvature.

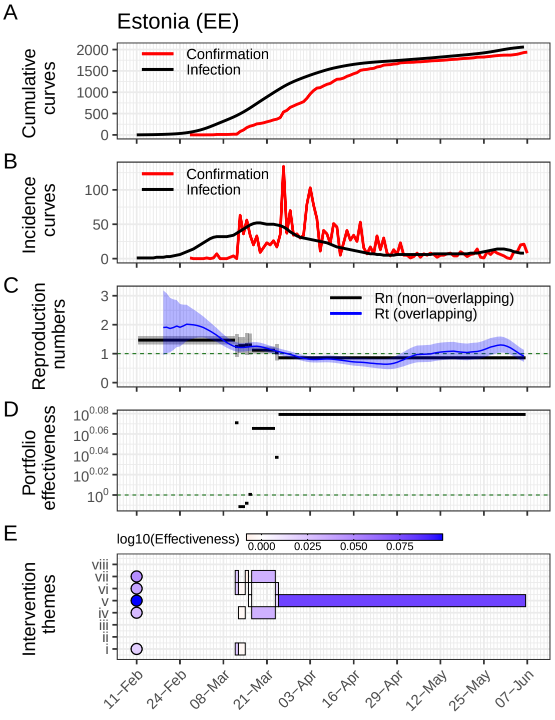

(B) **Estimates using uninformative initial condition.** A. The cumulative curves. B. The incidence curves. C. The instantaneous reproduction numbers R_n and R_t. D. The portfolio effectiveness η_n. E. The intervention $\eta_n^{(m)}$ (in rectangles) and aggregated $\eta^{(m)}$ (in circles) effectiveness.

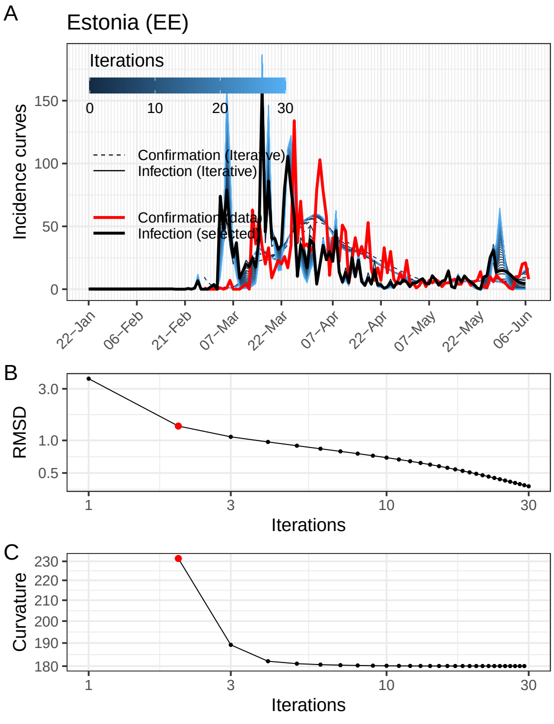

(C) **Deconvolution iterations using informative initial condition.** A. The RL deconvolution iterative curves and observed confirmation curve. B: The root-mean-square difference (RMSD) of all iterations. C: The curvature of RMSD. The red dot represents the selected iteration with maximum curvature.

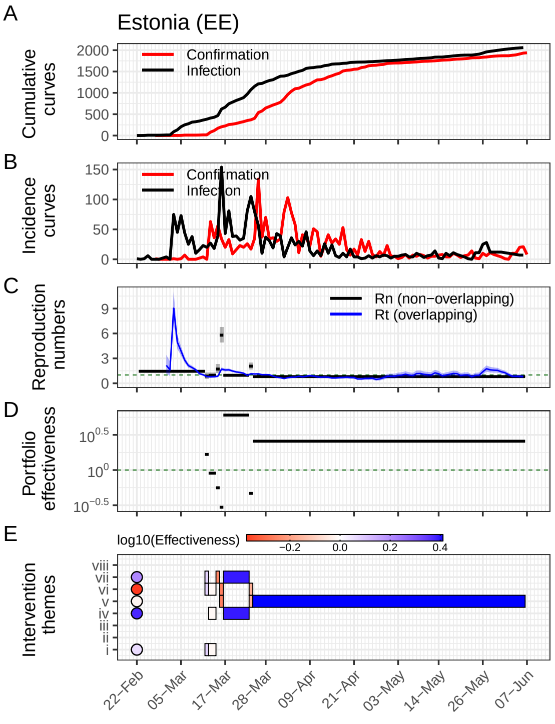

(D) **Estimates using informative initial condition.** A. The cumulative curves. B. The incidence curves. C. The instantaneous reproduction numbers R_n and R_t. D. The portfolio effectiveness η_n. E. The intervention $\eta_n^{(m)}$ (in rectangles) and aggregated $\eta^{(m)}$ (in circles) effectiveness.

FIGURE S12. **The reconstructed epidemic curves and subsequent evaluation of interventions implemented in Estonia.** Estimation using uninformative (baseline setting, A and B) and informative (alternative setting, C and D) initial condition.

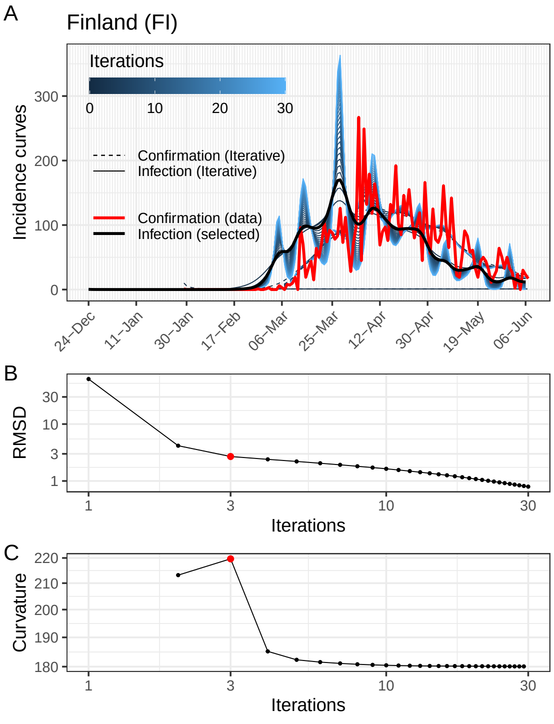

(A) **Deconvolution iterations using uninformative initial condition.** A. The RL deconvolution iterative curves and observed confirmation curve. B: The root-mean-square difference (RMSD) of all iterations. C: The curvature of RMSD. The red dot represents the selected iteration with maximum curvature.

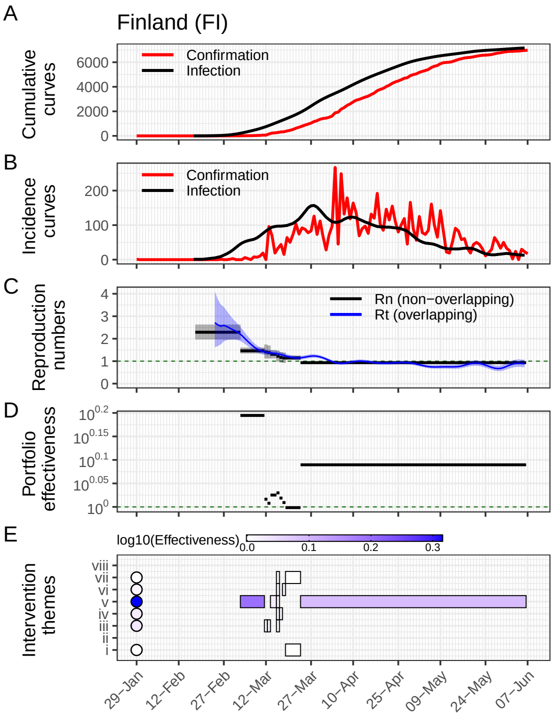

(B) **Estimates using uninformative initial condition.** A. The cumulative curves. B. The incidence curves. C. The instantaneous reproduction numbers R_n and R_t. D. The portfolio effectiveness η_n. E. The intervention $\eta_n^{(m)}$ (in rectangles) and aggregated $\eta^{(m)}$ (in circles) effectiveness.

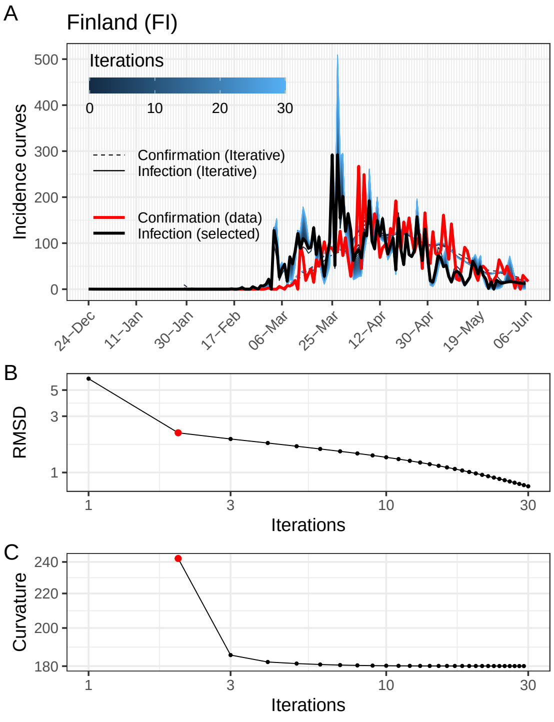

(C) **Deconvolution iterations using informative initial condition.** A. The RL deconvolution iterative curves and observed confirmation curve. B: The root-mean-square difference (RMSD) of all iterations. C: The curvature of RMSD. The red dot represents the selected iteration with maximum curvature.

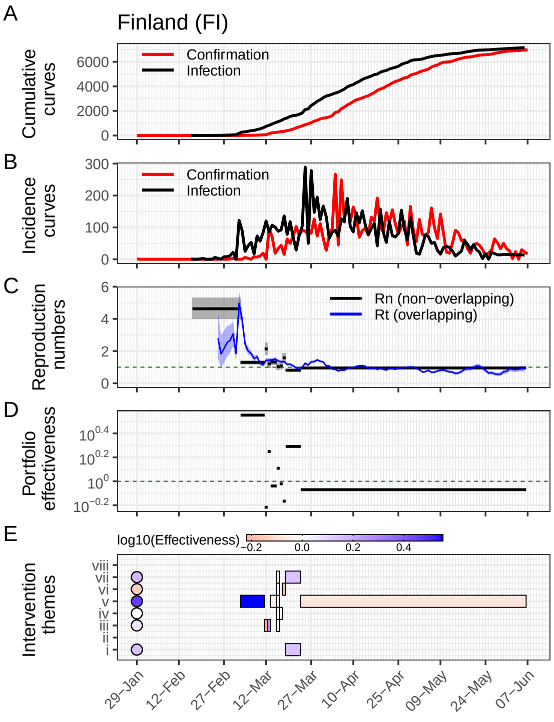

(D) **Estimates using informative initial condition.** A. The cumulative curves. B. The incidence curves. C. The instantaneous reproduction numbers R_n and R_t. D. The portfolio effectiveness η_n. E. The intervention $\eta_n^{(m)}$ (in rectangles) and aggregated $\eta^{(m)}$ (in circles) effectiveness.

FIGURE S13. **The reconstructed epidemic curves and subsequent evaluation of interventions implemented in Finland.** Estimation using uninformative (baseline setting, A and B) and informative (alternative setting, C and D) initial condition.

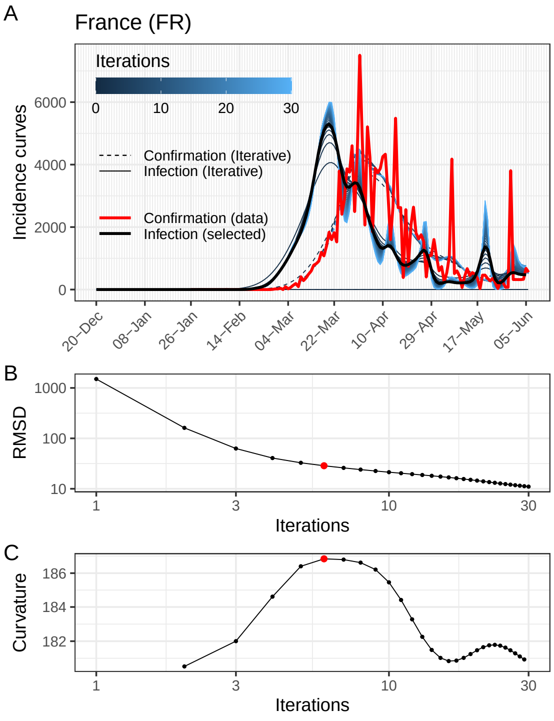

(A) **Deconvolution iterations using uninformative initial condition.** A. The RL deconvolution iterative curves and observed confirmation curve. B: The root-mean-square difference (RMSD) of all iterations. C: The curvature of RMSD. The red dot represents the selected iteration with maximum curvature.

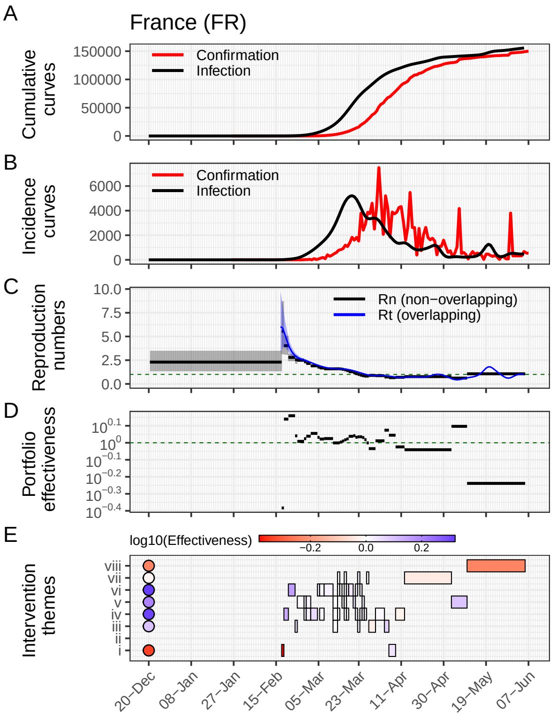

(B) **Estimates using uninformative initial condition.** A. The cumulative curves. B. The incidence curves. C. The instantaneous reproduction numbers R_n and R_t. D. The portfolio effectiveness η_n. E. The intervention $\eta_n^{(m)}$ (in rectangles) and aggregated $\eta^{(m)}$ (in circles) effectiveness.

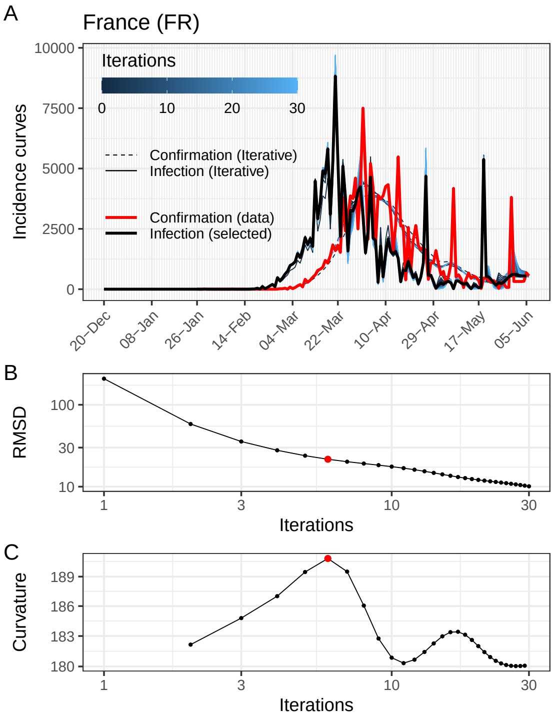

(C) **Deconvolution iterations using informative initial condition.** A. The RL deconvolution iterative curves and observed confirmation curve. B: The root-mean-square difference (RMSD) of all iterations. C: The curvature of RMSD. The red dot represents the selected iteration with maximum curvature.

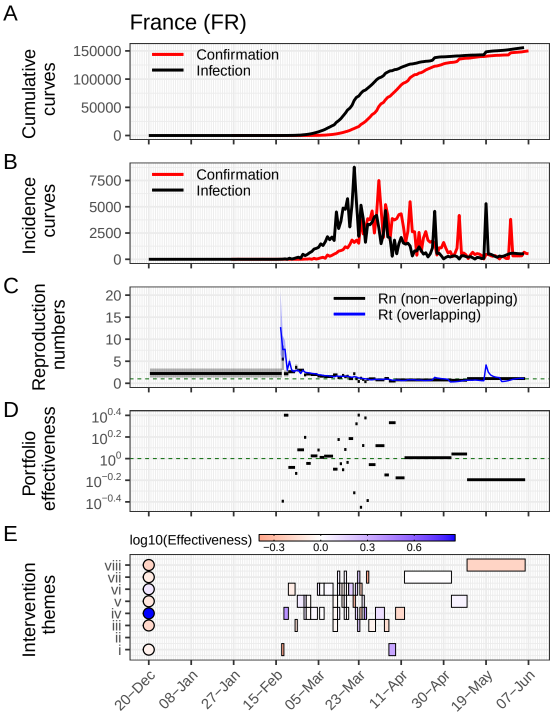

(D) **Estimates using informative initial condition.** A. The cumulative curves. B. The incidence curves. C. The instantaneous reproduction numbers R_n and R_t. D. The portfolio effectiveness η_n. E. The intervention $\eta_n^{(m)}$ (in rectangles) and aggregated $\eta^{(m)}$ (in circles) effectiveness.

FIGURE S14. **The reconstructed epidemic curves and subsequent evaluation of interventions implemented in France.** Estimation using uninformative (baseline setting, A and B) and informative (alternative setting, C and D) initial condition.

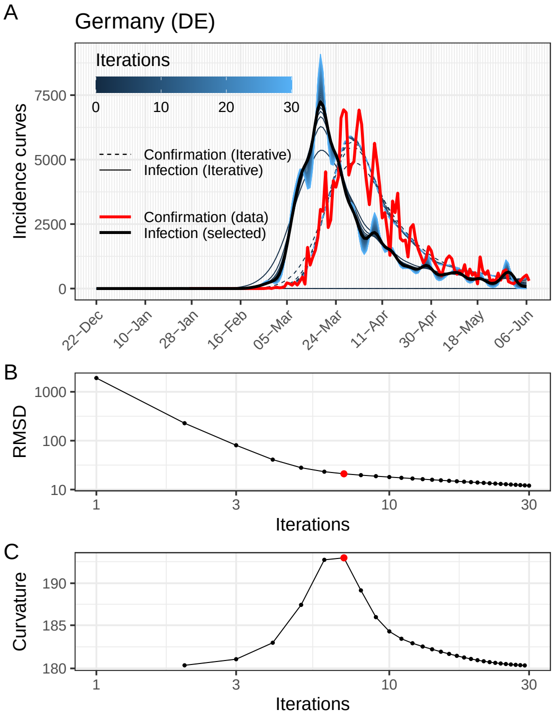

(A) **Deconvolution iterations using uninformative initial condition.** A. The RL deconvolution iterative curves and observed confirmation curve. B: The root-mean-square difference (RMSD) of all iterations. C: The curvature of RMSD. The red dot represents the selected iteration with maximum curvature.

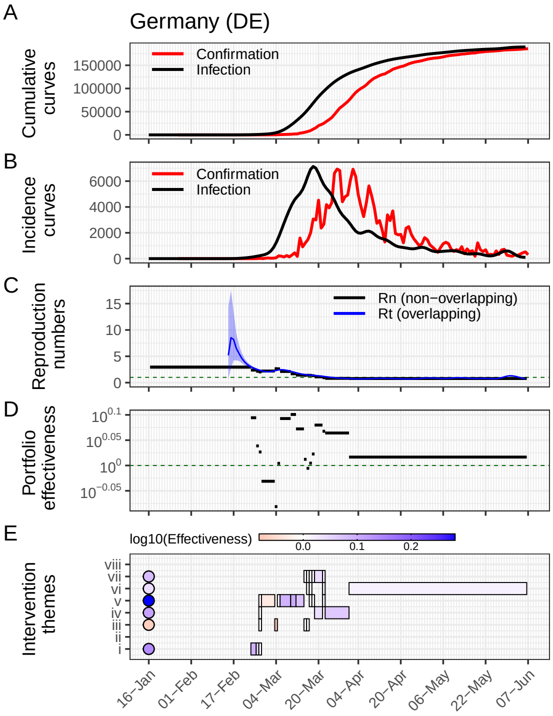

(B) **Estimates using uninformative initial condition.** A. The cumulative curves. B. The incidence curves. C. The instantaneous reproduction numbers R_n and R_t. D. The portfolio effectiveness η_n. E. The intervention $\eta_n^{(m)}$ (in rectangles) and aggregated $\eta^{(m)}$ (in circles) effectiveness.

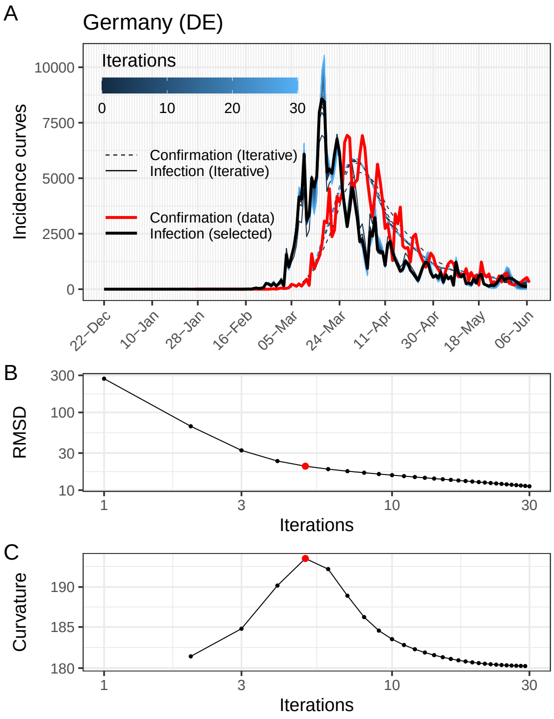

(C) **Deconvolution iterations using informative initial condition.** A. The RL deconvolution iterative curves and observed confirmation curve. B: The root-mean-square difference (RMSD) of all iterations. C: The curvature of RMSD. The red dot represents the selected iteration with maximum curvature.

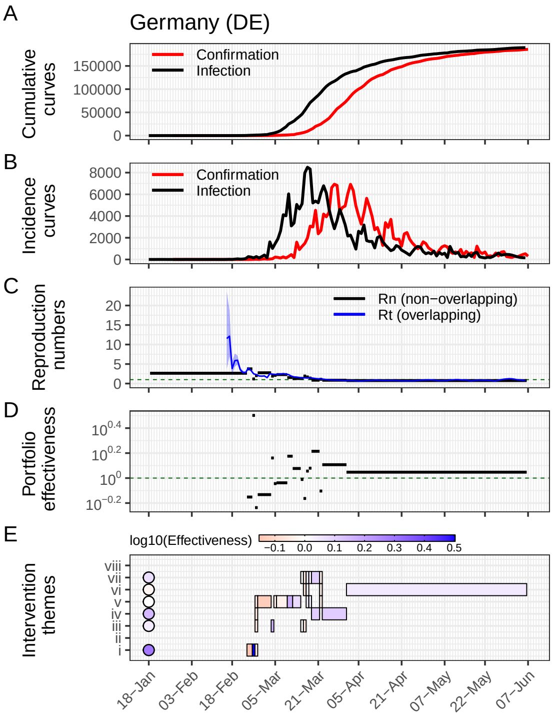

(D) **Estimates using informative initial condition.** A. The cumulative curves. B. The incidence curves. C. The instantaneous reproduction numbers R_n and R_t. D. The portfolio effectiveness η_n. E. The intervention $\eta_n^{(m)}$ (in rectangles) and aggregated $\eta^{(m)}$ (in circles) effectiveness.

FIGURE S15. **The reconstructed epidemic curves and subsequent evaluation of interventions implemented in Germany.** Estimation using uninformative (baseline setting, A and B) and informative (alternative setting, C and D) initial condition.

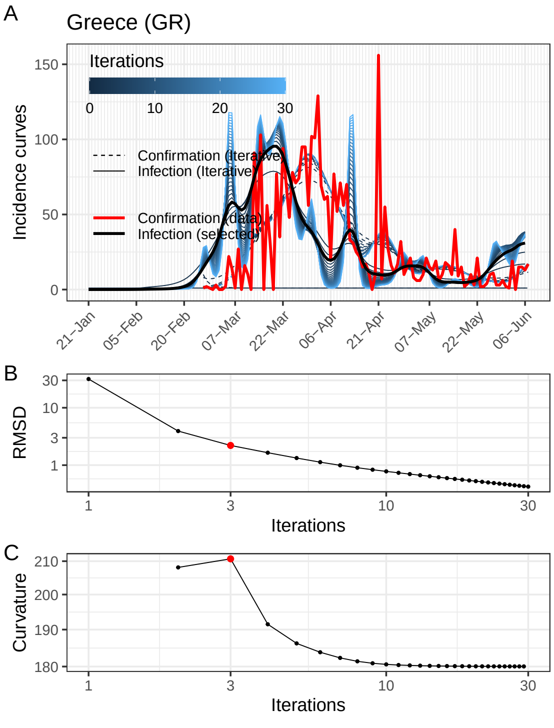

(A) **Deconvolution iterations using uninformative initial condition.** A. The RL deconvolution iterative curves and observed confirmation curve. B: The root-mean-square difference (RMSD) of all iterations. C: The curvature of RMSD. The red dot represents the selected iteration with maximum curvature.

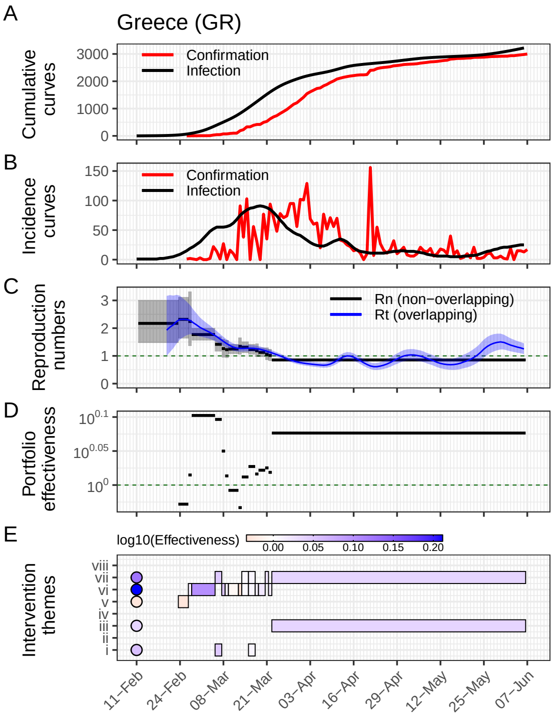

(B) **Estimates using uninformative initial condition.** A. The cumulative curves. B. The incidence curves. C. The instantaneous reproduction numbers R_n and R_t. D. The portfolio effectiveness η_n. E. The intervention $\eta_n^{(m)}$ (in rectangles) and aggregated $\eta^{(m)}$ (in circles) effectiveness.

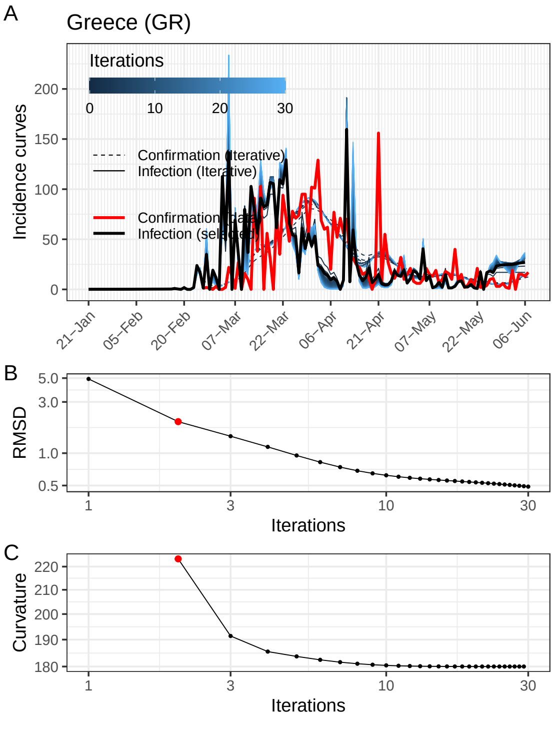

(C) **Deconvolution iterations using informative initial condition.** A. The RL deconvolution iterative curves and observed confirmation curve. B: The root-mean-square difference (RMSD) of all iterations. C: The curvature of RMSD. The red dot represents the selected iteration with maximum curvature.

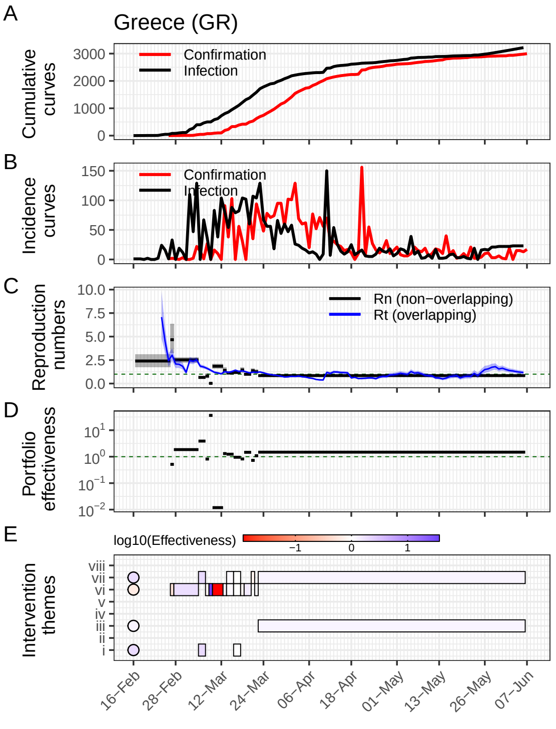

(D) **Estimates using informative initial condition.** A. The cumulative curves. B. The incidence curves. C. The instantaneous reproduction numbers R_n and R_t. D. The portfolio effectiveness η_n. E. The intervention $\eta_n^{(m)}$ (in rectangles) and aggregated $\eta^{(m)}$ (in circles) effectiveness.

Figure S16. **The reconstructed epidemic curves and subsequent evaluation of interventions implemented in Greece.** Estimation using uninformative (baseline setting, A and B) and informative (alternative setting, C and D) initial condition.

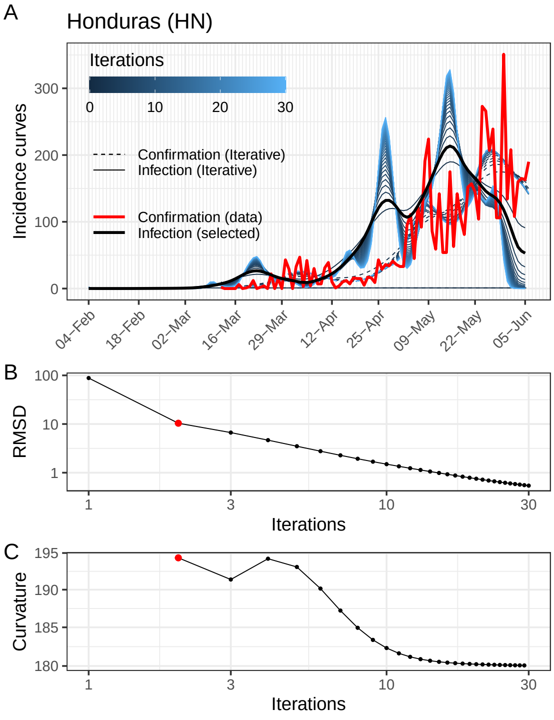

(A) **Deconvolution iterations using uninformative initial condition.** A. The RL deconvolution iterative curves and observed confirmation curve. B: The root-mean-square difference (RMSD) of all iterations. C: The curvature of RMSD. The red dot represents the selected iteration with maximum curvature.

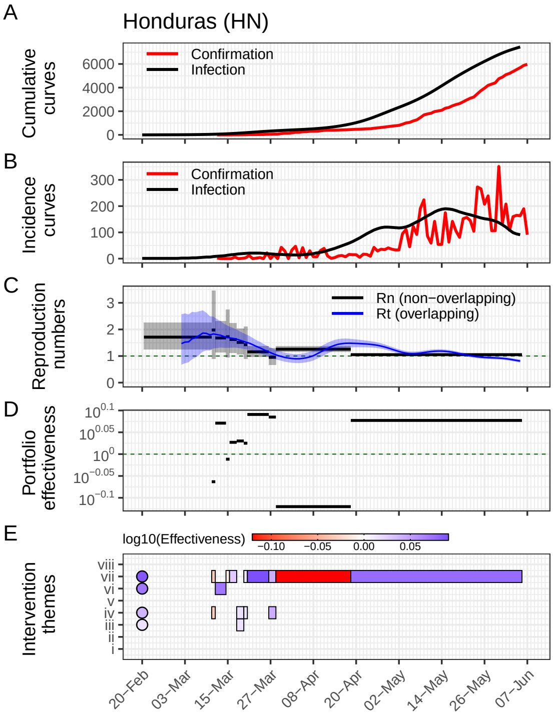

(B) **Estimates using uninformative initial condition.** A. The cumulative curves. B. The incidence curves. C. The instantaneous reproduction numbers R_n and R_t. D. The portfolio effectiveness η_n. E. The intervention $\eta_n^{(m)}$ (in rectangles) and aggregated $\eta^{(m)}$ (in circles) effectiveness.

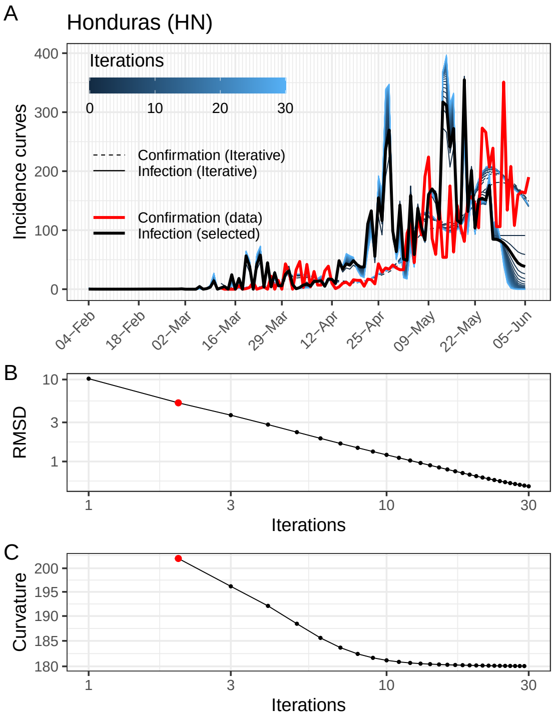

(C) **Deconvolution iterations using informative initial condition.** A. The RL deconvolution iterative curves and observed confirmation curve. B: The root-mean-square difference (RMSD) of all iterations. C: The curvature of RMSD. The red dot represents the selected iteration with maximum curvature.

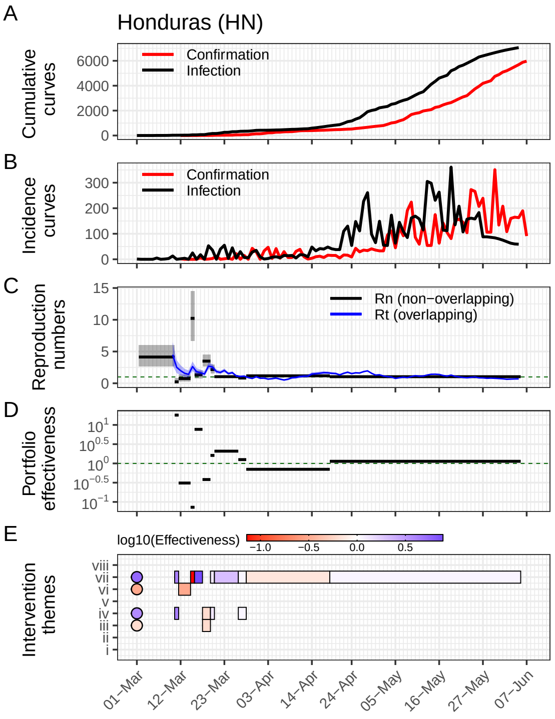

(D) **Estimates using informative initial condition.** A. The cumulative curves. B. The incidence curves. C. The instantaneous reproduction numbers R_n and R_t. D. The portfolio effectiveness η_n. E. The intervention $\eta_n^{(m)}$ (in rectangles) and aggregated $\eta^{(m)}$ (in circles) effectiveness.

FIGURE S17. **The reconstructed epidemic curves and subsequent evaluation of interventions implemented in Honduras.** Estimation using uninformative (baseline setting, A and B) and informative (alternative setting, C and D) initial condition.

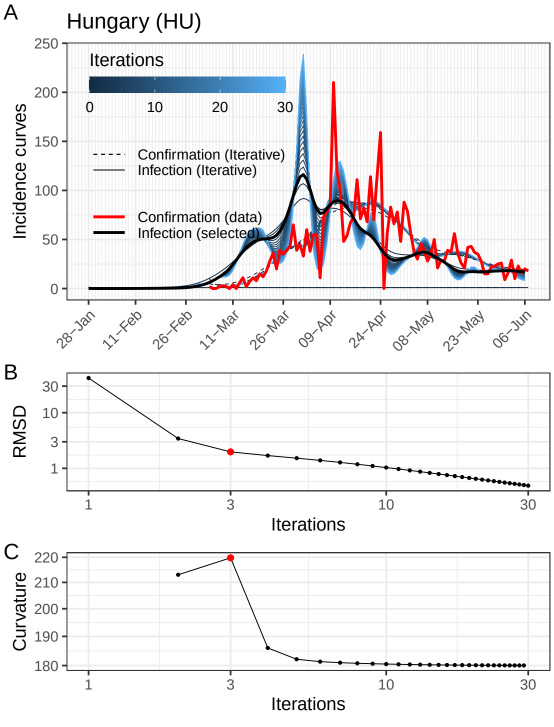

(A) **Deconvolution iterations using uninformative initial condition.** A. The RL deconvolution iterative curves and observed confirmation curve. B: The root-mean-square difference (RMSD) of all iterations. C: The curvature of RMSD. The red dot represents the selected iteration with maximum curvature.

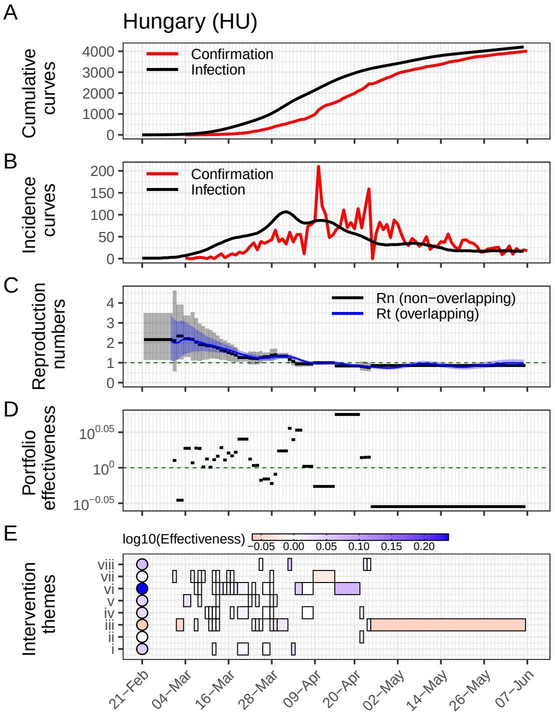

(B) **Estimates using uninformative initial condition.** A. The cumulative curves. B. The incidence curves. C. The instantaneous reproduction numbers R_n and R_t. D. The portfolio effectiveness η_n. E. The intervention $\eta_n^{(m)}$ (in rectangles) and aggregated $\eta^{(m)}$ (in circles) effectiveness.

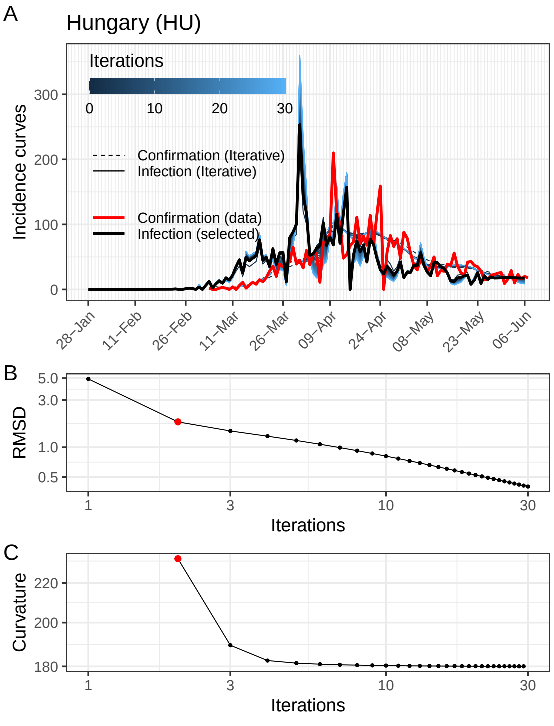

(C) **Deconvolution iterations using informative initial condition.** A. The RL deconvolution iterative curves and observed confirmation curve. B: The root-mean-square difference (RMSD) of all iterations. C: The curvature of RMSD. The red dot represents the selected iteration with maximum curvature.

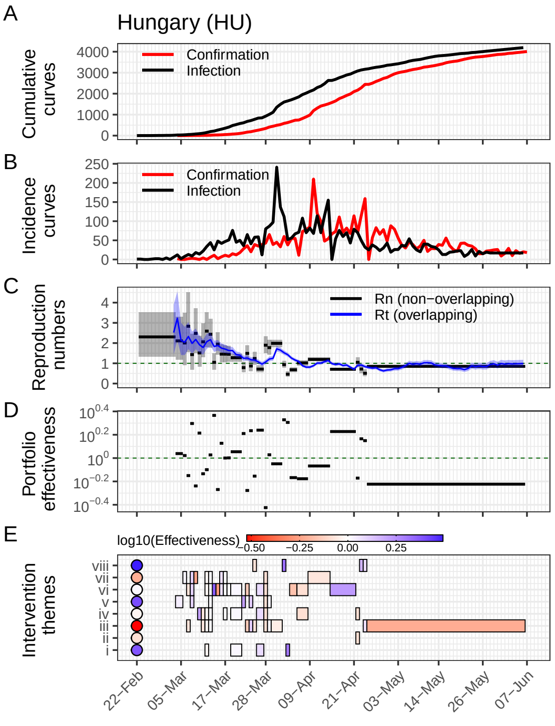

(D) **Estimates using informative initial condition.** A. The cumulative curves. B. The incidence curves. C. The instantaneous reproduction numbers R_n and R_t. D. The portfolio effectiveness η_n. E. The intervention $\eta_n^{(m)}$ (in rectangles) and aggregated $\eta^{(m)}$ (in circles) effectiveness.

FIGURE S18. **The reconstructed epidemic curves and subsequent evaluation of interventions implemented in Hungary.** Estimation using uninformative (baseline setting, A and B) and informative (alternative setting, C and D) initial condition.

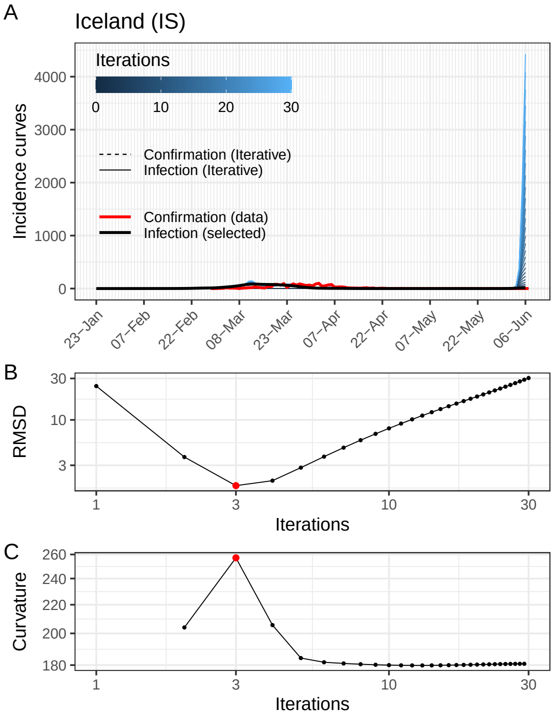

(A) **Deconvolution iterations using uninformative initial condition.** A. The RL deconvolution iterative curves and observed confirmation curve. B: The root-mean-square difference (RMSD) of all iterations. C: The curvature of RMSD. The red dot represents the selected iteration with maximum curvature.

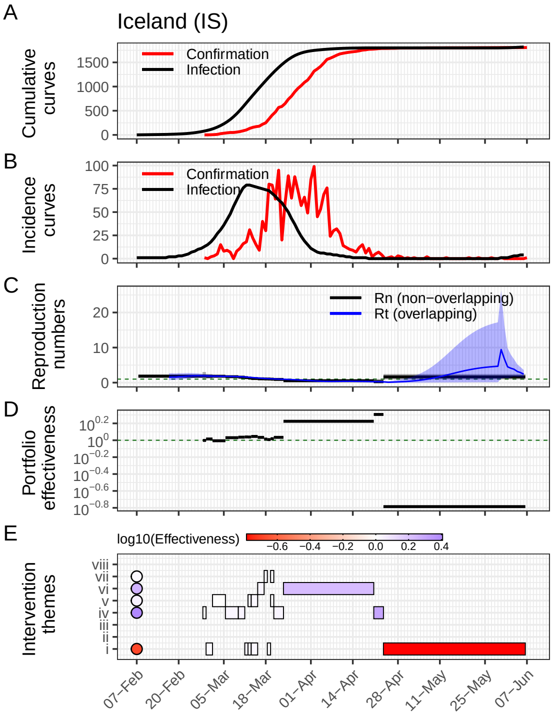

(B) **Estimates using uninformative initial condition.** A. The cumulative curves. B. The incidence curves. C. The instantaneous reproduction numbers R_n and R_t. D. The portfolio effectiveness η_n. E. The intervention $\eta_n^{(m)}$ (in rectangles) and aggregated $\eta^{(m)}$ (in circles) effectiveness.

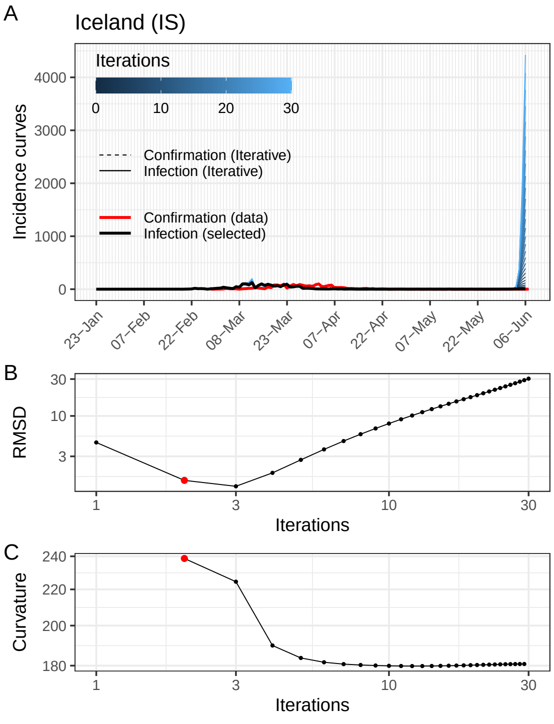

(C) **Deconvolution iterations using informative initial condition.** A. The RL deconvolution iterative curves and observed confirmation curve. B: The root-mean-square difference (RMSD) of all iterations. C: The curvature of RMSD. The red dot represents the selected iteration with maximum curvature.

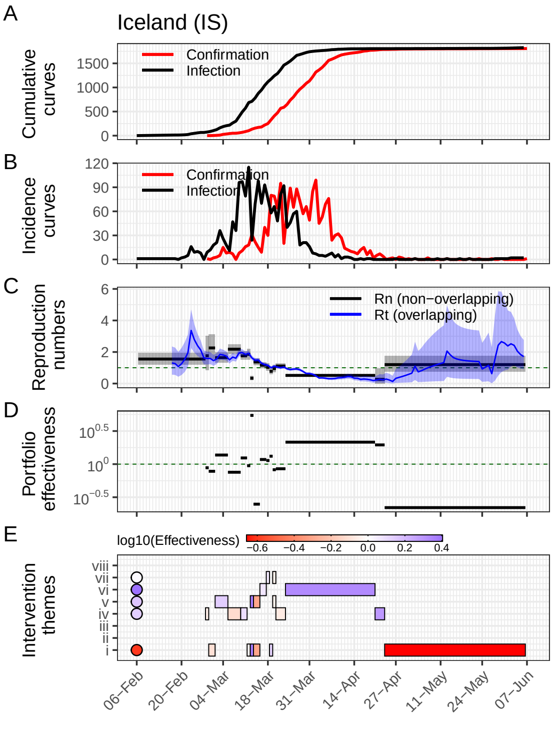

(D) **Estimates using informative initial condition.** A. The cumulative curves. B. The incidence curves. C. The instantaneous reproduction numbers R_n and R_t. D. The portfolio effectiveness η_n. E. The intervention $\eta_n^{(m)}$ (in rectangles) and aggregated $\eta^{(m)}$ (in circles) effectiveness.

FIGURE S19. **The reconstructed epidemic curves and subsequent evaluation of interventions implemented in Iceland.** Estimation using uninformative (baseline setting, A and B) and informative (alternative setting, C and D) initial condition.

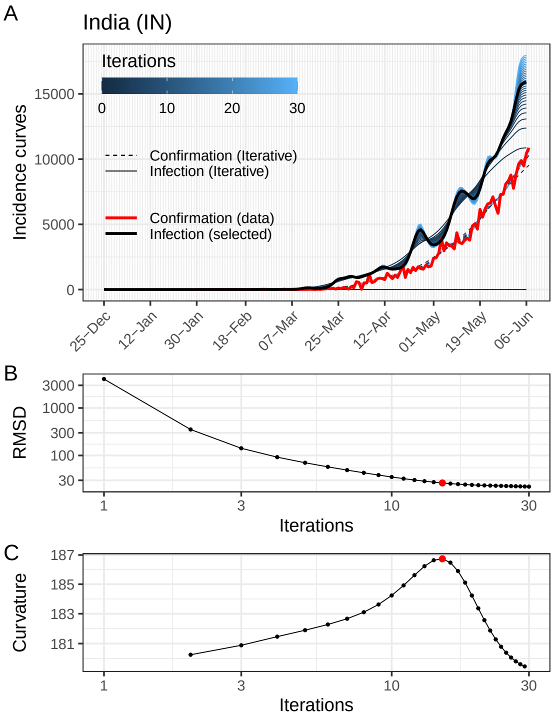

(A) **Deconvolution iterations using uninformative initial condition.** A. The RL deconvolution iterative curves and observed confirmation curve. B: The root-mean-square difference (RMSD) of all iterations. C: The curvature of RMSD. The red dot represents the selected iteration with maximum curvature.

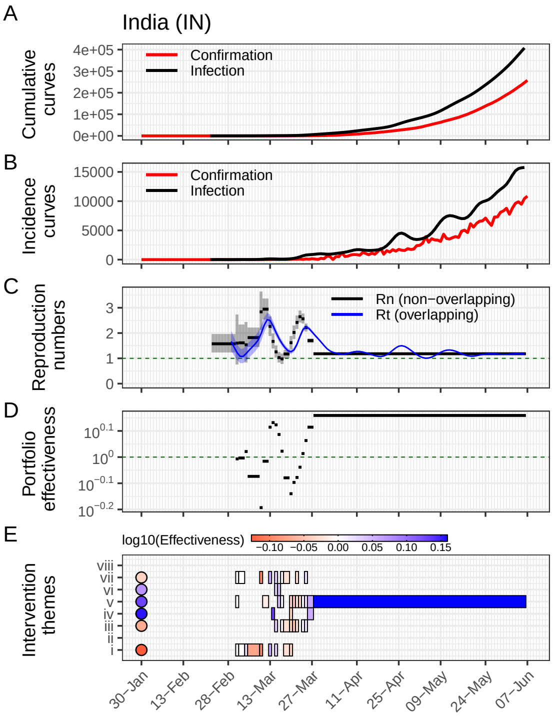

(B) **Estimates using uninformative initial condition.** A. The cumulative curves. B. The incidence curves. C. The instantaneous reproduction numbers R_n and R_t. D. The portfolio effectiveness η_n. E. The intervention $\eta_n^{(m)}$ (in rectangles) and aggregated $\eta^{(m)}$ (in circles) effectiveness.

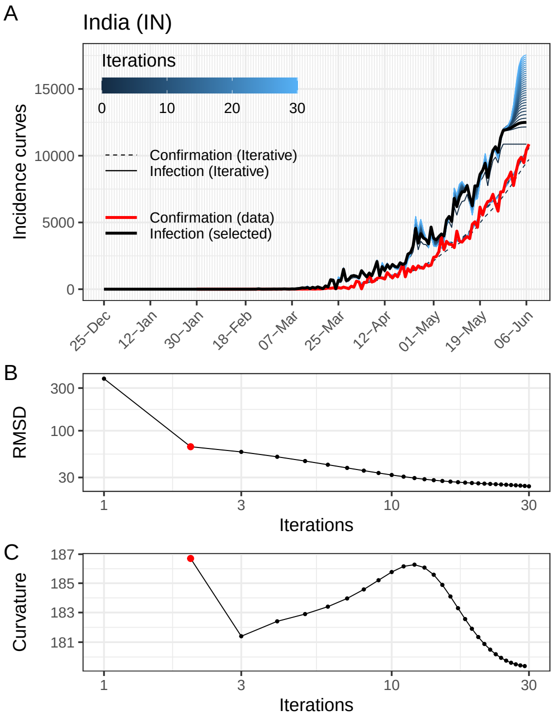

(C) **Deconvolution iterations using informative initial condition.** A. The RL deconvolution iterative curves and observed confirmation curve. B: The root-mean-square difference (RMSD) of all iterations. C: The curvature of RMSD. The red dot represents the selected iteration with maximum curvature.

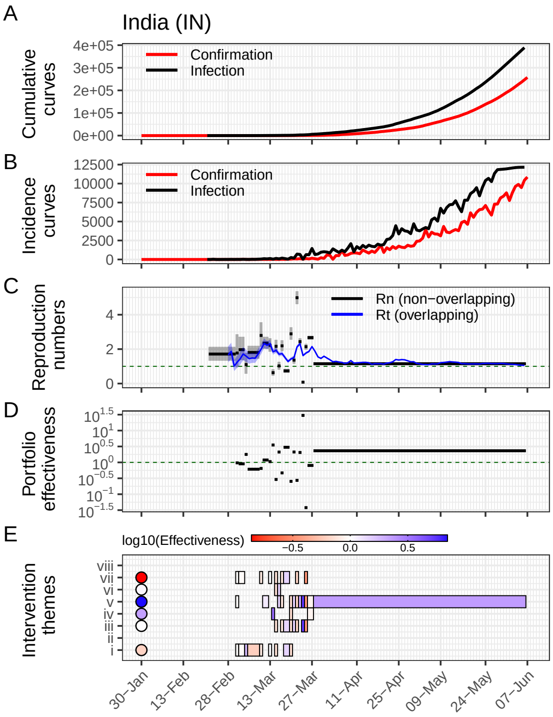

(D) **Estimates using informative initial condition.** A. The cumulative curves. B. The incidence curves. C. The instantaneous reproduction numbers R_n and R_t. D. The portfolio effectiveness η_n. E. The intervention $\eta_n^{(m)}$ (in rectangles) and aggregated $\eta^{(m)}$ (in circles) effectiveness.

FIGURE S20. **The reconstructed epidemic curves and subsequent evaluation of interventions implemented in India.** Estimation using uninformative (baseline setting, A and B) and informative (alternative setting, C and D) initial condition.

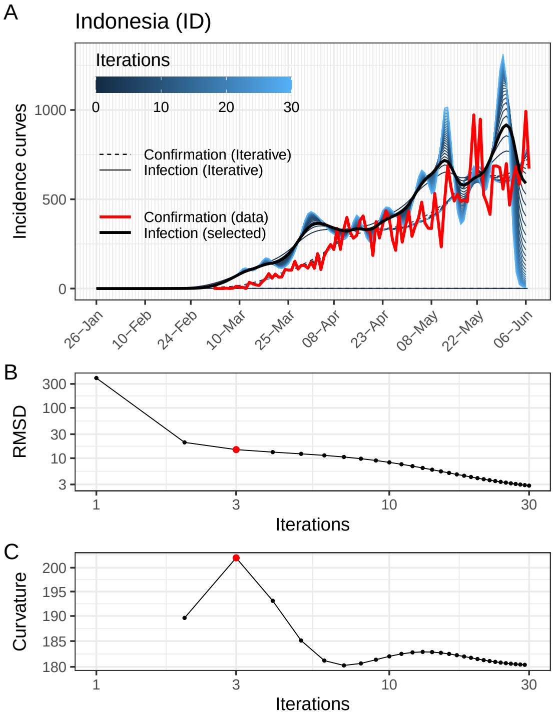

(A) **Deconvolution iterations using uninformative initial condition.** A. The RL deconvolution iterative curves and observed confirmation curve. B: The root-mean-square difference (RMSD) of all iterations. C: The curvature of RMSD. The red dot represents the selected iteration with maximum curvature.

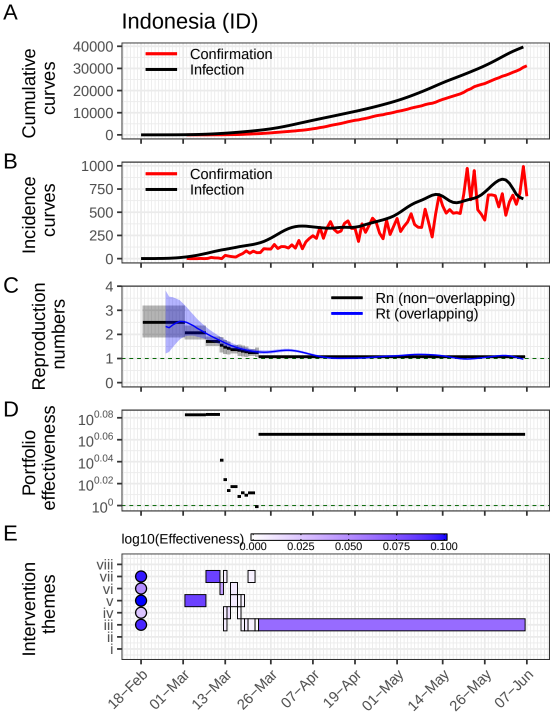

(B) **Estimates using uninformative initial condition.** A. The cumulative curves. B. The incidence curves. C. The instantaneous reproduction numbers R_n and R_t. D. The portfolio effectiveness η_n. E. The intervention $\eta_n^{(m)}$ (in rectangles) and aggregated $\eta^{(m)}$ (in circles) effectiveness.

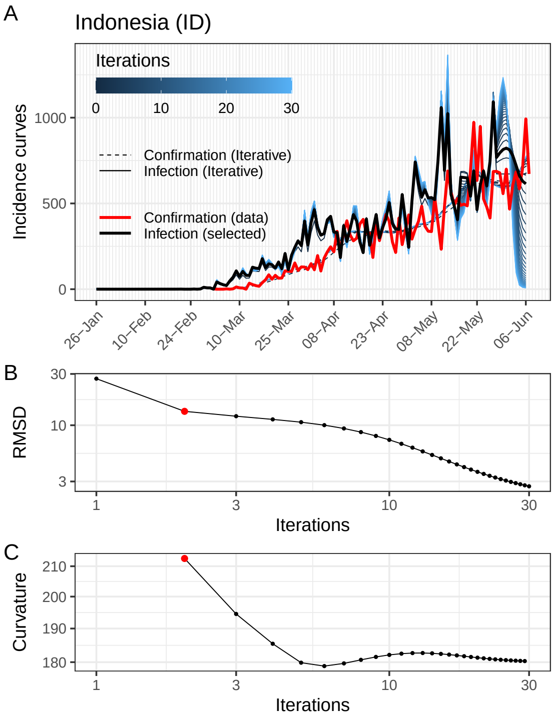

(C) **Deconvolution iterations using informative initial condition.** A. The RL deconvolution iterative curves and observed confirmation curve. B: The root-mean-square difference (RMSD) of all iterations. C: The curvature of RMSD. The red dot represents the selected iteration with maximum curvature.

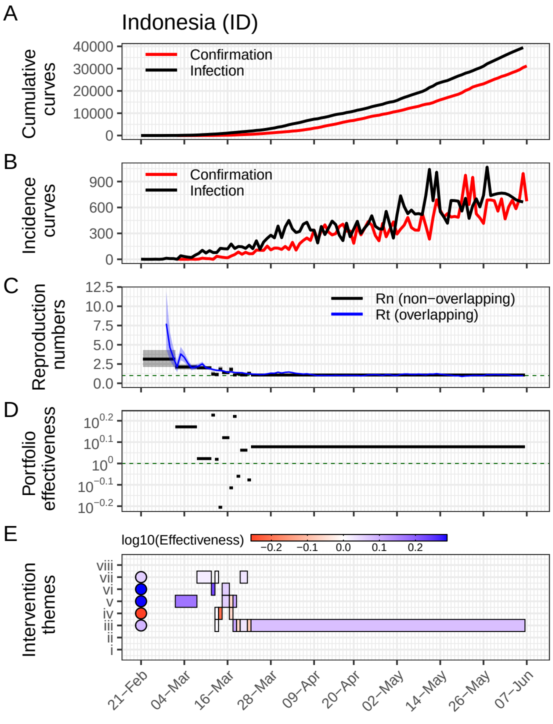

(D) **Estimates using informative initial condition.** A. The cumulative curves. B. The incidence curves. C. The instantaneous reproduction numbers R_n and R_t. D. The portfolio effectiveness η_n. E. The intervention $\eta_n^{(m)}$ (in rectangles) and aggregated $\eta^{(m)}$ (in circles) effectiveness.

FIGURE S21. **The reconstructed epidemic curves and subsequent evaluation of interventions implemented in Indonesia.** Estimation using uninformative (baseline setting, A and B) and informative (alternative setting, C and D) initial condition.

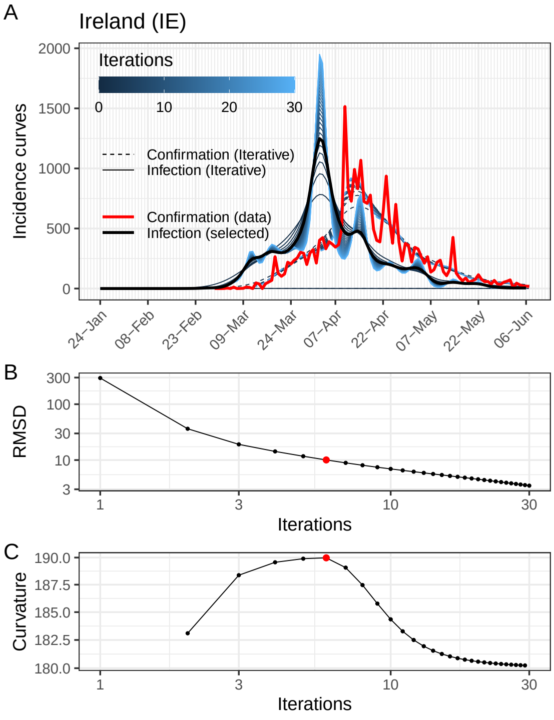

(A) **Deconvolution iterations using uninformative initial condition.** A. The RL deconvolution iterative curves and observed confirmation curve. B: The root-mean-square difference (RMSD) of all iterations. C: The curvature of RMSD. The red dot represents the selected iteration with maximum curvature.

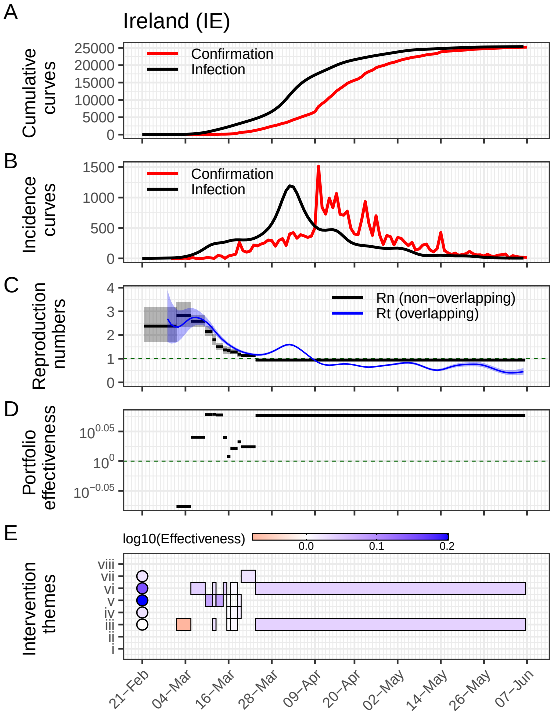

(B) **Estimates using uninformative initial condition.** A. The cumulative curves. B. The incidence curves. C. The instantaneous reproduction numbers R_n and R_t. D. The portfolio effectiveness η_n. E. The intervention $\eta_n^{(m)}$ (in rectangles) and aggregated $\eta^{(m)}$ (in circles) effectiveness.

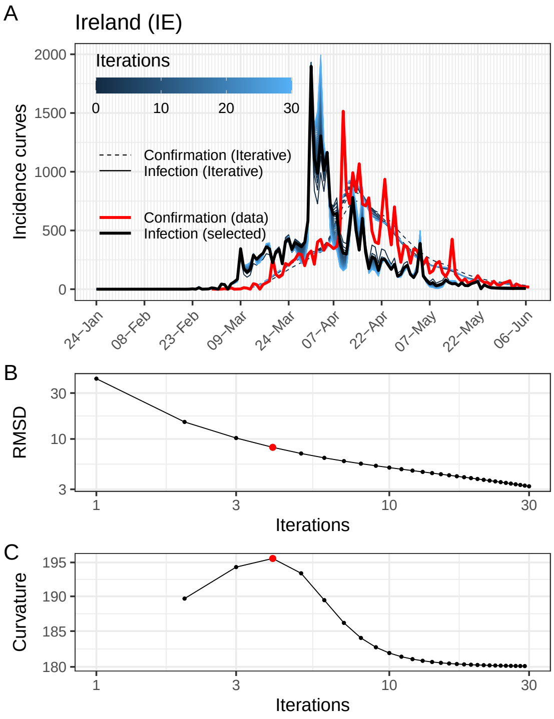

(C) **Deconvolution iterations using informative initial condition.** A. The RL deconvolution iterative curves and observed confirmation curve. B: The root-mean-square difference (RMSD) of all iterations. C: The curvature of RMSD. The red dot represents the selected iteration with maximum curvature.

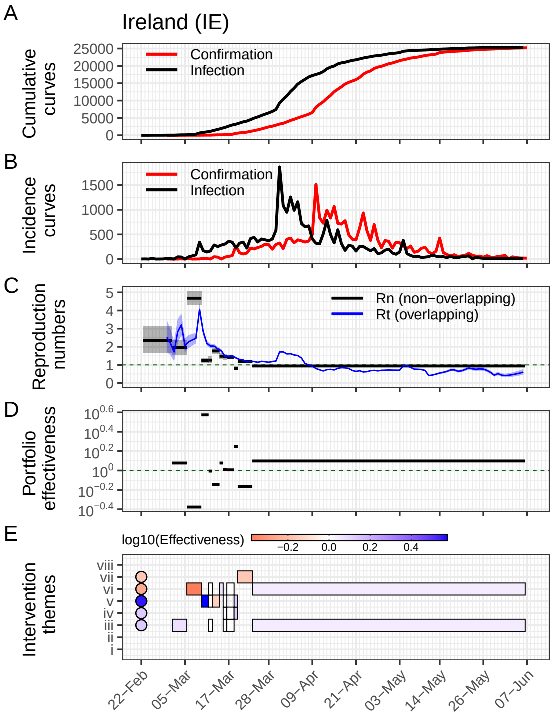

(D) **Estimates using informative initial condition.** A. The cumulative curves. B. The incidence curves. C. The instantaneous reproduction numbers R_n and R_t. D. The portfolio effectiveness η_n. E. The intervention $\eta_n^{(m)}$ (in rectangles) and aggregated $\eta^{(m)}$ (in circles) effectiveness.

FIGURE S22. **The reconstructed epidemic curves and subsequent evaluation of interventions implemented in Ireland.** Estimation using uninformative (baseline setting, A and B) and informative (alternative setting, C and D) initial condition.

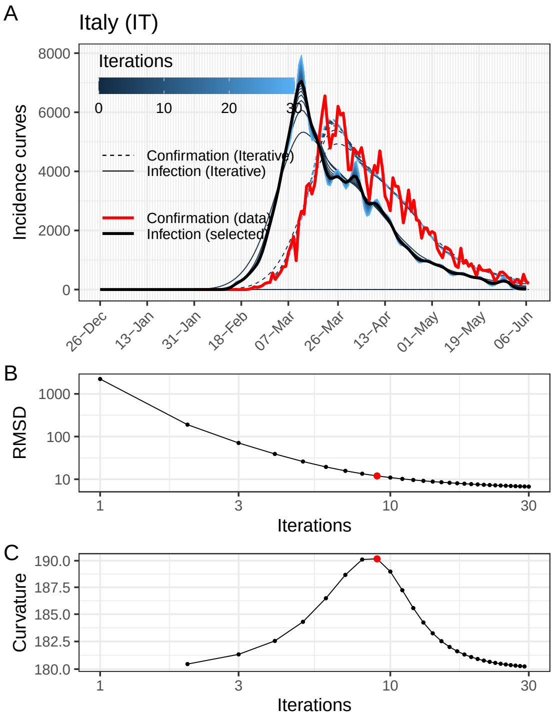

(A) **Deconvolution iterations using uninformative initial condition.** A. The RL deconvolution iterative curves and observed confirmation curve. B: The root-mean-square difference (RMSD) of all iterations. C: The curvature of RMSD. The red dot represents the selected iteration with maximum curvature.

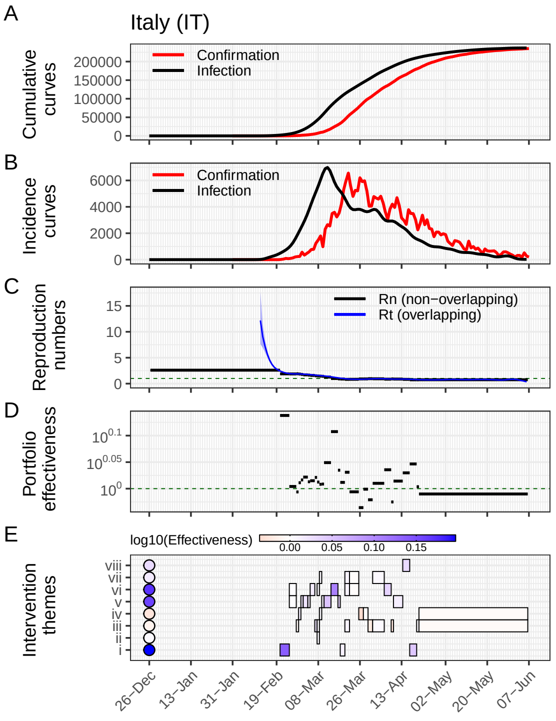

(B) **Estimates using uninformative initial condition.** A. The cumulative curves. B. The incidence curves. C. The instantaneous reproduction numbers R_n and R_t. D. The portfolio effectiveness η_n. E. The intervention $\eta_n^{(m)}$ (in rectangles) and aggregated $\eta^{(m)}$ (in circles) effectiveness.

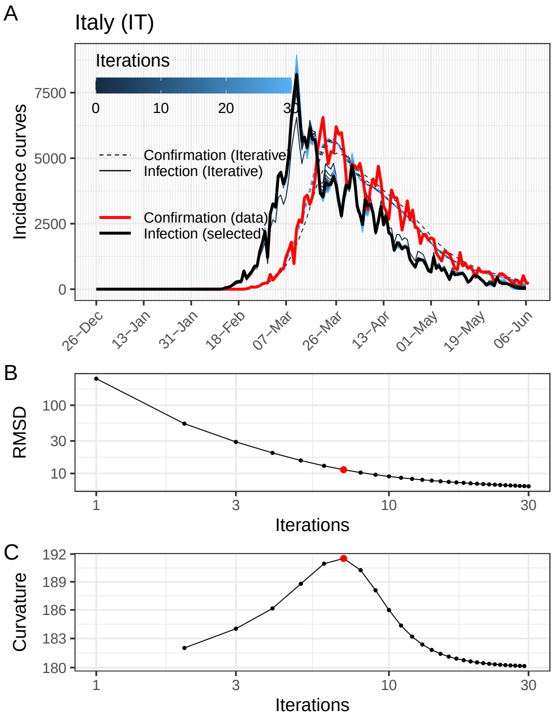

(C) **Deconvolution iterations using informative initial condition.** A. The RL deconvolution iterative curves and observed confirmation curve. B: The root-mean-square difference (RMSD) of all iterations. C: The curvature of RMSD. The red dot represents the selected iteration with maximum curvature.

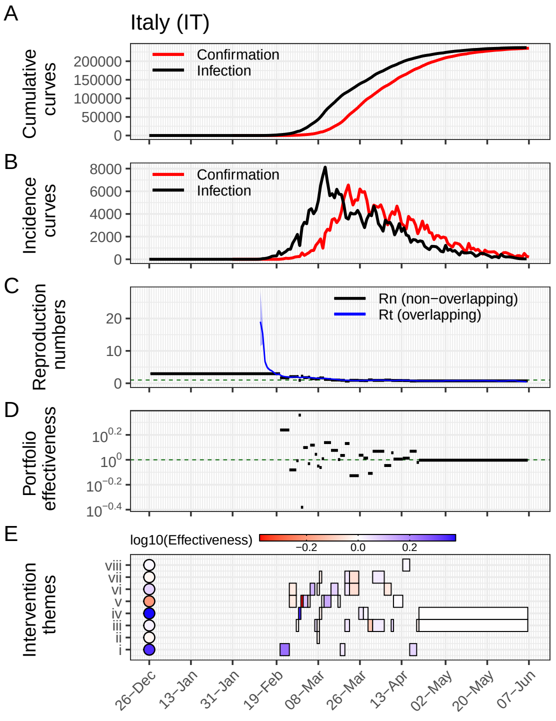

(D) **Estimates using informative initial condition.** A. The cumulative curves. B. The incidence curves. C. The instantaneous reproduction numbers R_n and R_t. D. The portfolio effectiveness η_n. E. The intervention $\eta_n^{(m)}$ (in rectangles) and aggregated $\eta^{(m)}$ (in circles) effectiveness.

FIGURE S23. **The reconstructed epidemic curves and subsequent evaluation of interventions implemented in Italy.** Estimation using uninformative (baseline setting, A and B) and informative (alternative setting, C and D) initial condition.

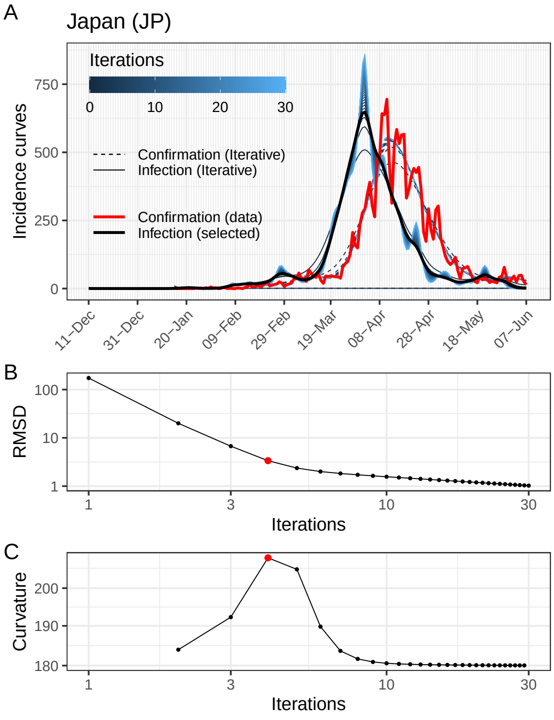

(A) **Deconvolution iterations using uninformative initial condition.** A. The RL deconvolution iterative curves and observed confirmation curve. B: The root-mean-square difference (RMSD) of all iterations. C: The curvature of RMSD. The red dot represents the selected iteration with maximum curvature.

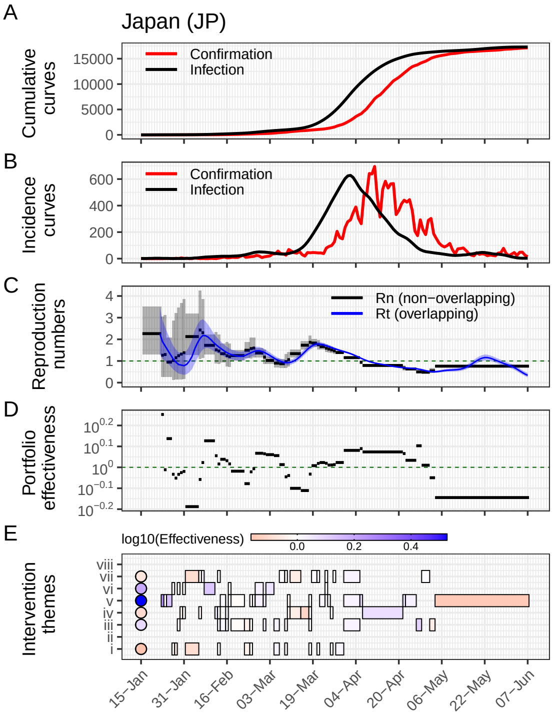

(B) **Estimates using uninformative initial condition.** A. The cumulative curves. B. The incidence curves. C. The instantaneous reproduction numbers R_n and R_t. D. The portfolio effectiveness η_n. E. The intervention $\eta_n^{(m)}$ (in rectangles) and aggregated $\eta^{(m)}$ (in circles) effectiveness.

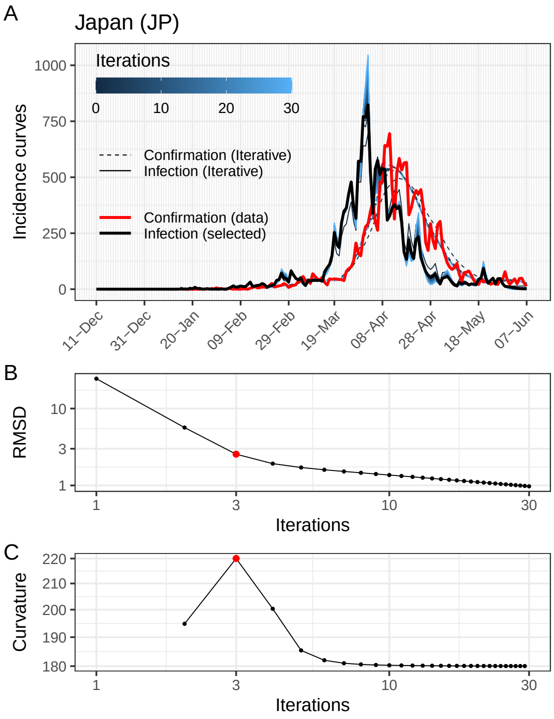

(C) **Deconvolution iterations using informative initial condition.** A. The RL deconvolution iterative curves and observed confirmation curve. B: The root-mean-square difference (RMSD) of all iterations. C: The curvature of RMSD. The red dot represents the selected iteration with maximum curvature.

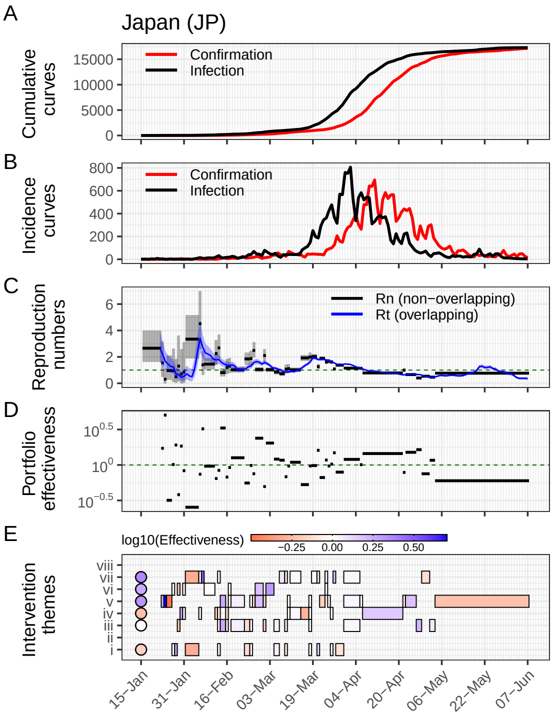

(D) **Estimates using informative initial condition.** A. The cumulative curves. B. The incidence curves. C. The instantaneous reproduction numbers R_n and R_t. D. The portfolio effectiveness η_n. E. The intervention $\eta_n^{(m)}$ (in rectangles) and aggregated $\eta^{(m)}$ (in circles) effectiveness.

FIGURE S24. **The reconstructed epidemic curves and subsequent evaluation of interventions implemented in Japan.** Estimation using uninformative (baseline setting, A and B) and informative (alternative setting, C and D) initial condition.

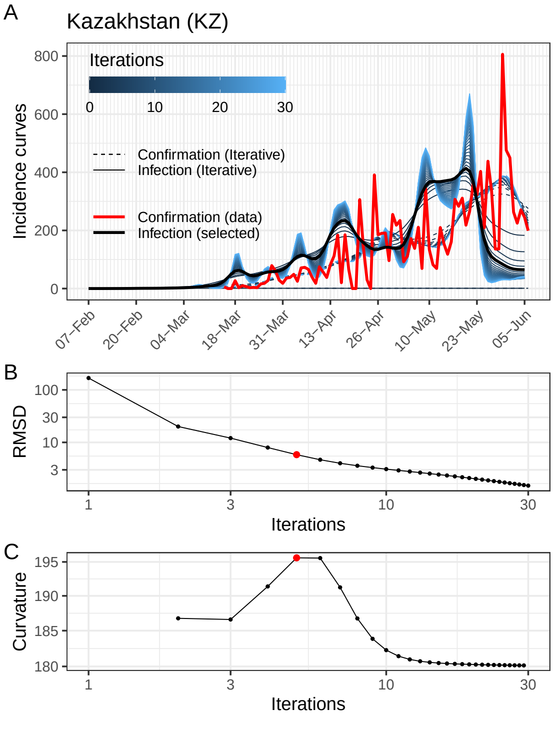

(A) **Deconvolution iterations using uninformative initial condition.** A. The RL deconvolution iterative curves and observed confirmation curve. B: The root-mean-square difference (RMSD) of all iterations. C: The curvature of RMSD. The red dot represents the selected iteration with maximum curvature.

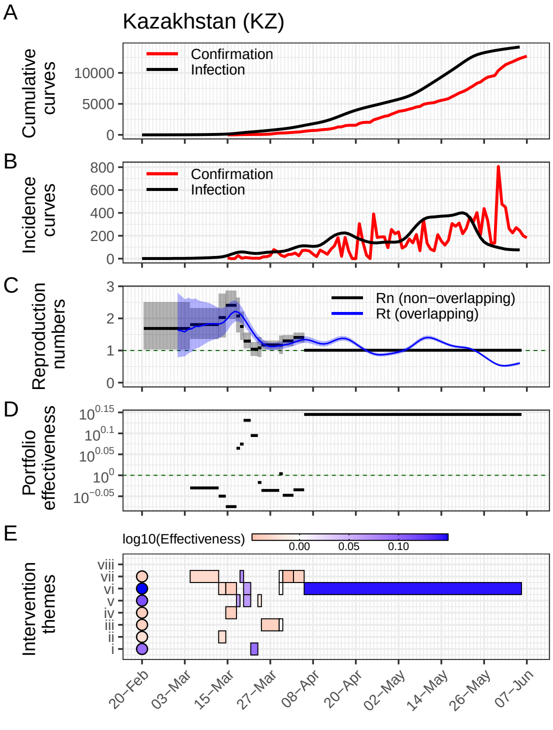

(B) **Estimates using uninformative initial condition.** A. The cumulative curves. B. The incidence curves. C. The instantaneous reproduction numbers R_n and R_t. D. The portfolio effectiveness η_n. E. The intervention $\eta_n^{(m)}$ (in rectangles) and aggregated $\eta^{(m)}$ (in circles) effectiveness.

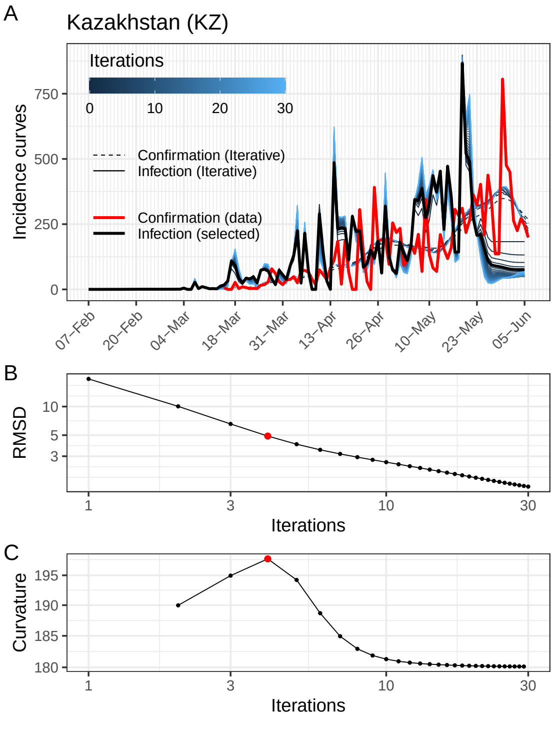

(C) **Deconvolution iterations using informative initial condition.** A. The RL deconvolution iterative curves and observed confirmation curve. B: The root-mean-square difference (RMSD) of all iterations. C: The curvature of RMSD. The red dot represents the selected iteration with maximum curvature.

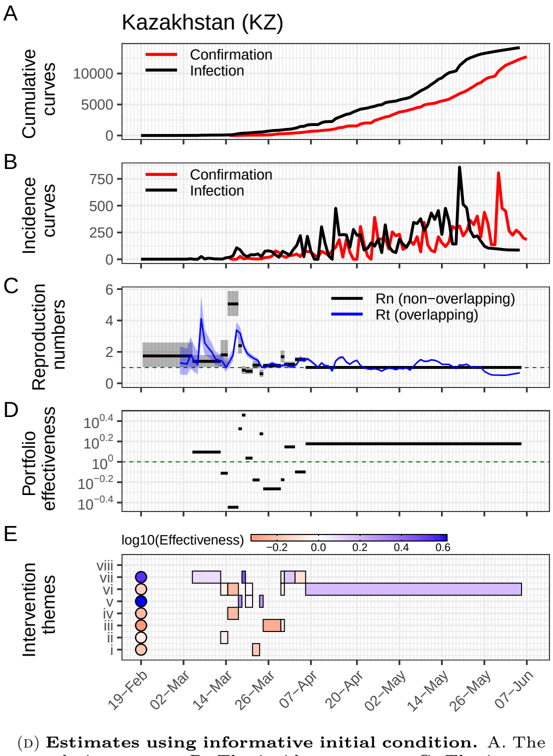

(D) **Estimates using informative initial condition.** A. The cumulative curves. B. The incidence curves. C. The instantaneous reproduction numbers R_n and R_t. D. The portfolio effectiveness η_n. E. The intervention $\eta_n^{(m)}$ (in rectangles) and aggregated $\eta^{(m)}$ (in circles) effectiveness.

FIGURE S25. **The reconstructed epidemic curves and subsequent evaluation of interventions implemented in Kazakhstan.** Estimation using uninformative (baseline setting, A and B) and informative (alternative setting, C and D) initial condition.

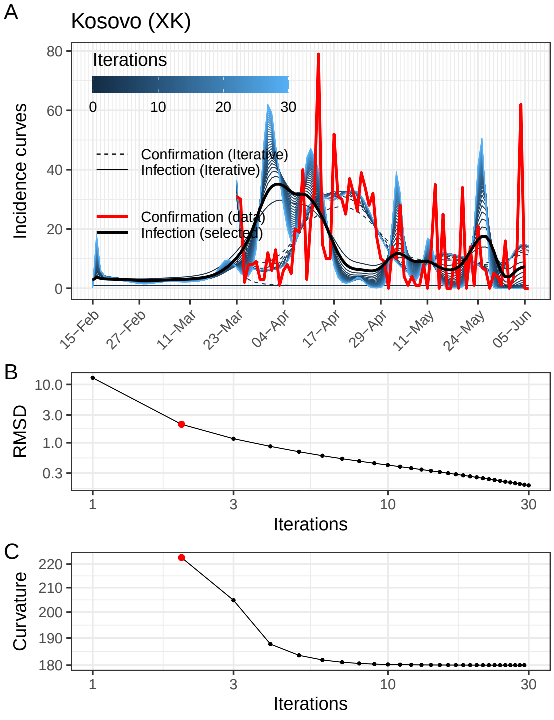

(A) **Deconvolution iterations using uninformative initial condition.** A. The RL deconvolution iterative curves and observed confirmation curve. B: The root-mean-square difference (RMSD) of all iterations. C: The curvature of RMSD. The red dot represents the selected iteration with maximum curvature.

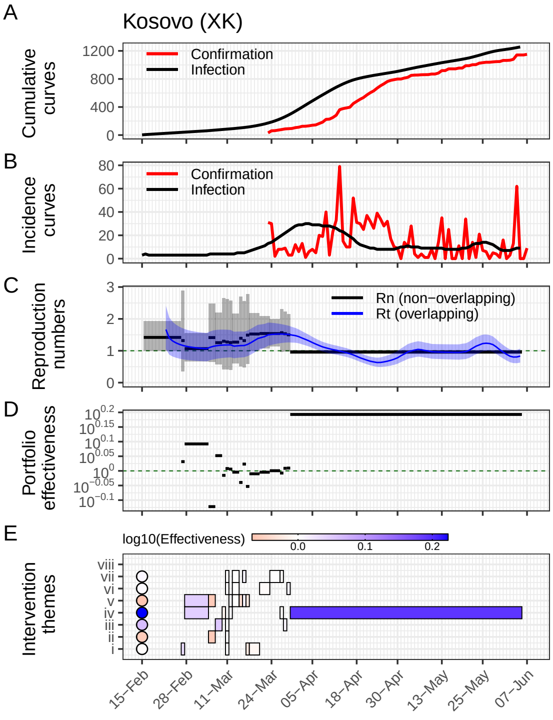

(B) **Estimates using uninformative initial condition.** A. The cumulative curves. B. The incidence curves. C. The instantaneous reproduction numbers R_n and R_t. D. The portfolio effectiveness η_n. E. The intervention $\eta_n^{(m)}$ (in rectangles) and aggregated $\eta^{(m)}$ (in circles) effectiveness.

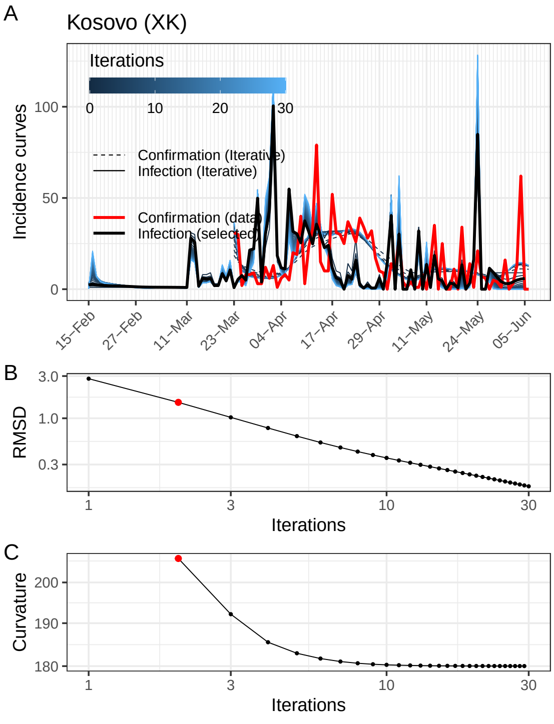

(C) **Deconvolution iterations using informative initial condition.** A. The RL deconvolution iterative curves and observed confirmation curve. B: The root-mean-square difference (RMSD) of all iterations. C: The curvature of RMSD. The red dot represents the selected iteration with maximum curvature.

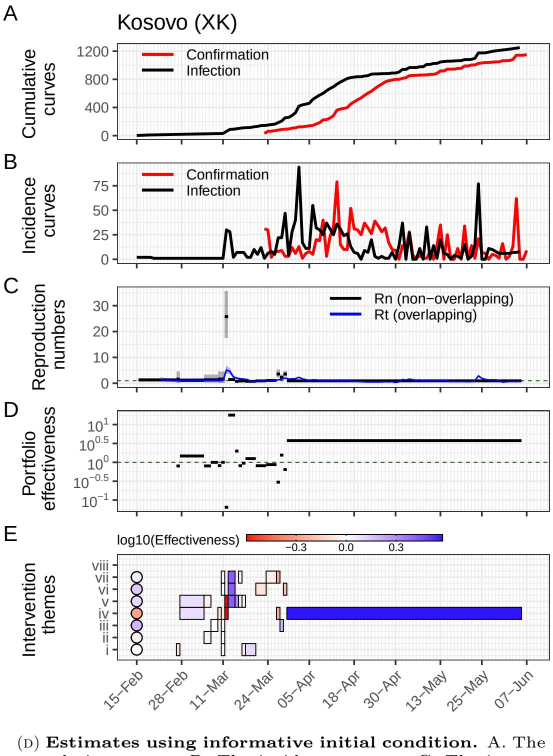

(D) **Estimates using informative initial condition.** A. The cumulative curves. B. The incidence curves. C. The instantaneous reproduction numbers R_n and R_t. D. The portfolio effectiveness η_n. E. The intervention $\eta_n^{(m)}$ (in rectangles) and aggregated $\eta^{(m)}$ (in circles) effectiveness.

FIGURE S26. **The reconstructed epidemic curves and subsequent evaluation of interventions implemented in Kosovo.** Estimation using uninformative (baseline setting, A and B) and informative (alternative setting, C and D) initial condition.

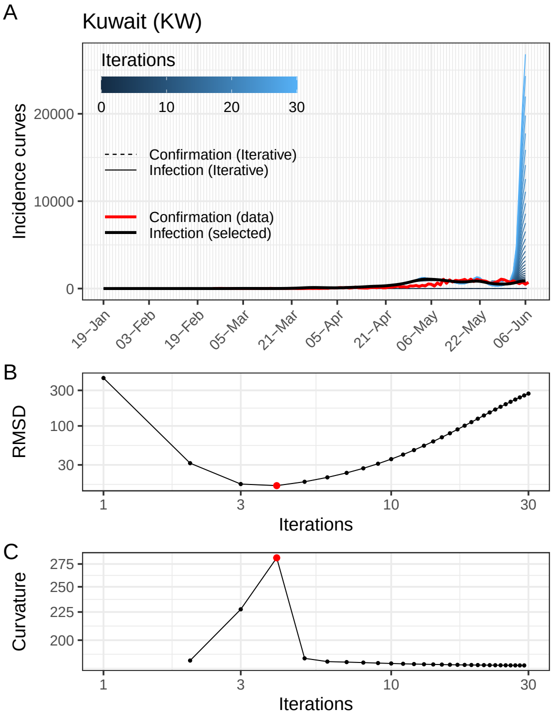

(A) **Deconvolution iterations using uninformative initial condition.** A. The RL deconvolution iterative curves and observed confirmation curve. B: The root-mean-square difference (RMSD) of all iterations. C: The curvature of RMSD. The red dot represents the selected iteration with maximum curvature.

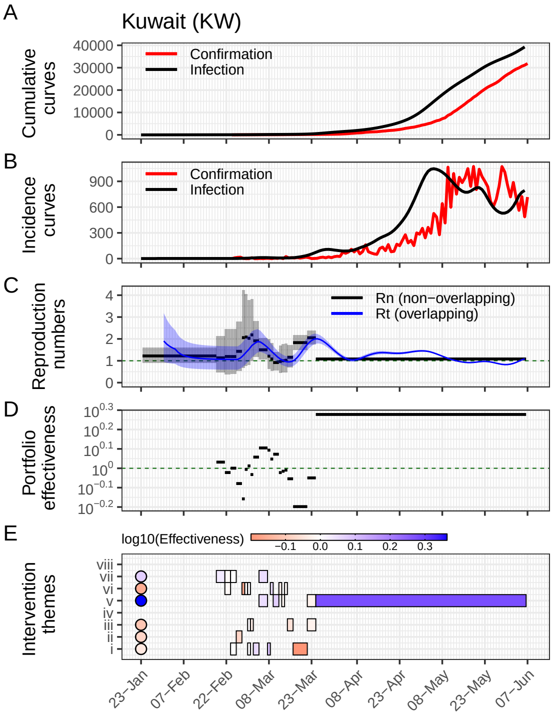

(B) **Estimates using uninformative initial condition.** A. The cumulative curves. B. The incidence curves. C. The instantaneous reproduction numbers R_n and R_t. D. The portfolio effectiveness η_n. E. The intervention $\eta_n^{(m)}$ (in rectangles) and aggregated $\eta^{(m)}$ (in circles) effectiveness.

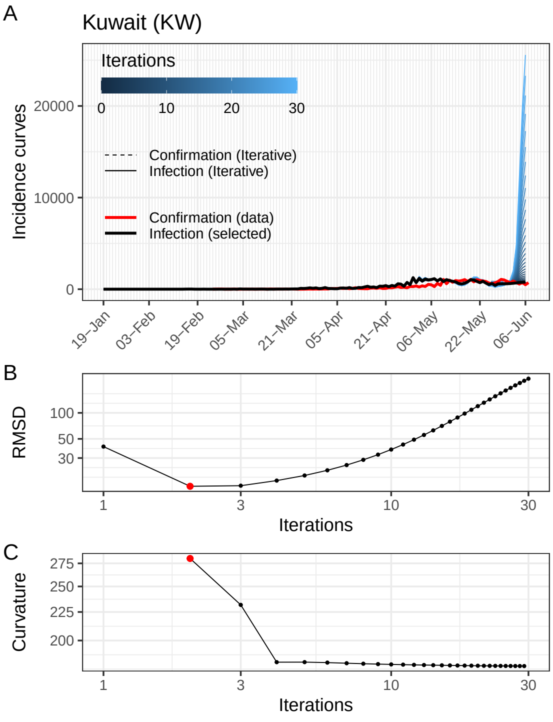

(C) **Deconvolution iterations using informative initial condition.** A. The RL deconvolution iterative curves and observed confirmation curve. B: The root-mean-square difference (RMSD) of all iterations. C: The curvature of RMSD. The red dot represents the selected iteration with maximum curvature.

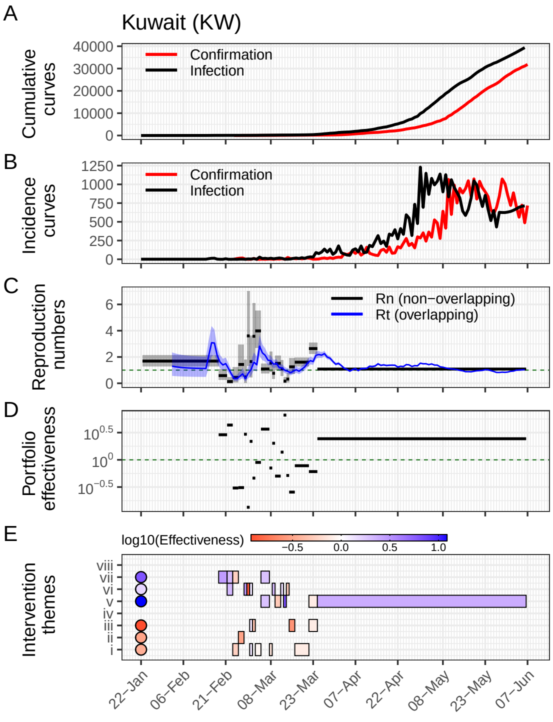

(D) **Estimates using informative initial condition.** A. The cumulative curves. B. The incidence curves. C. The instantaneous reproduction numbers R_n and R_t. D. The portfolio effectiveness η_n. E. The intervention $\eta_n^{(m)}$ (in rectangles) and aggregated $\eta^{(m)}$ (in circles) effectiveness.

Figure S27. **The reconstructed epidemic curves and subsequent evaluation of interventions implemented in Kuwait.** Estimation using uninformative (baseline setting, A and B) and informative (alternative setting, C and D) initial condition.

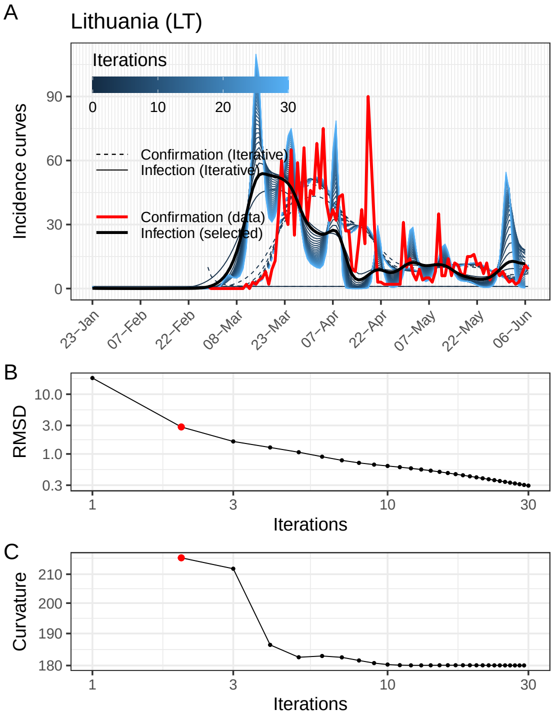

(A) **Deconvolution iterations using uninformative initial condition.** A. The RL deconvolution iterative curves and observed confirmation curve. B: The root-mean-square difference (RMSD) of all iterations. C: The curvature of RMSD. The red dot represents the selected iteration with maximum curvature.

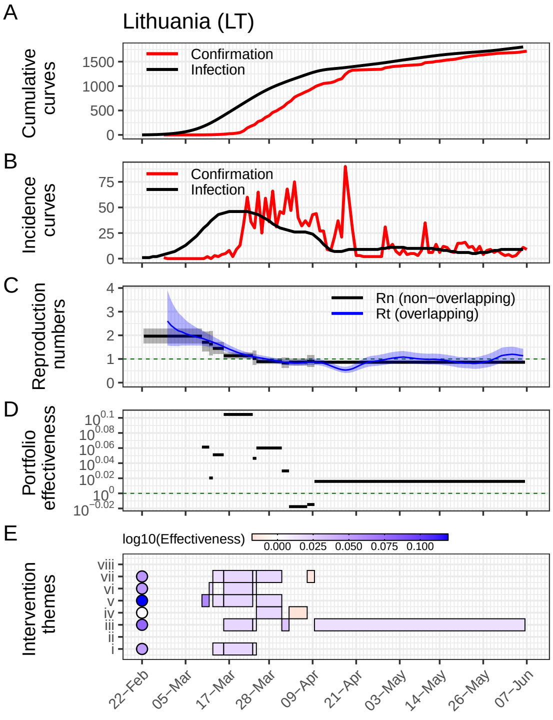

(B) **Estimates using uninformative initial condition.** A. The cumulative curves. B. The incidence curves. C. The instantaneous reproduction numbers R_n and R_t. D. The portfolio effectiveness η_n. E. The intervention $\eta_n^{(m)}$ (in rectangles) and aggregated $\eta^{(m)}$ (in circles) effectiveness.

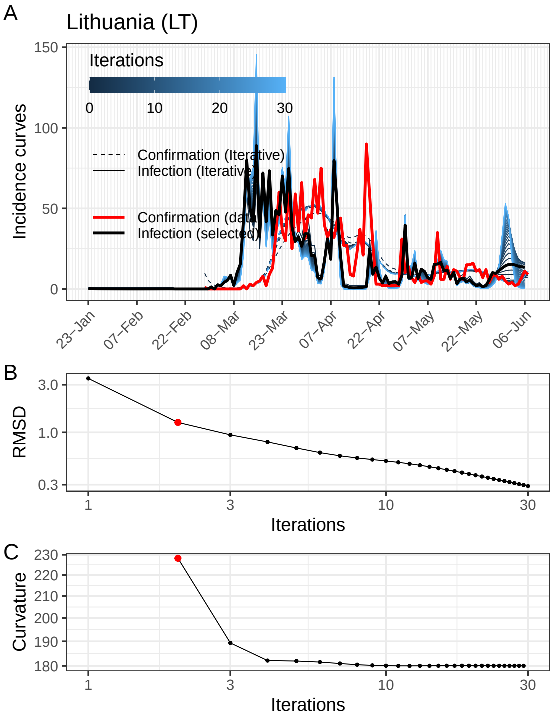

(C) **Deconvolution iterations using informative initial condition.** A. The RL deconvolution iterative curves and observed confirmation curve. B: The root-mean-square difference (RMSD) of all iterations. C: The curvature of RMSD. The red dot represents the selected iteration with maximum curvature.

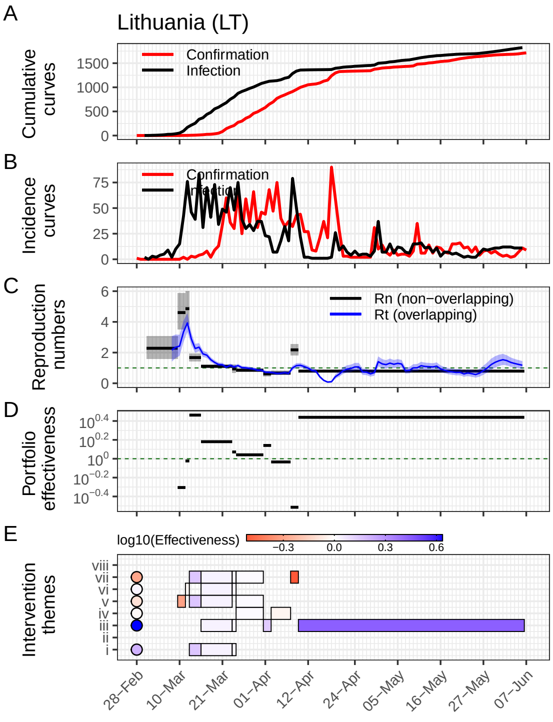

(D) **Estimates using informative initial condition.** A. The cumulative curves. B. The incidence curves. C. The instantaneous reproduction numbers R_n and R_t. D. The portfolio effectiveness η_n. E. The intervention $\eta_n^{(m)}$ (in rectangles) and aggregated $\eta^{(m)}$ (in circles) effectiveness.

Figure S28. **The reconstructed epidemic curves and subsequent evaluation of interventions implemented in Lithuania.** Estimation using uninformative (baseline setting, A and B) and informative (alternative setting, C and D) initial condition.

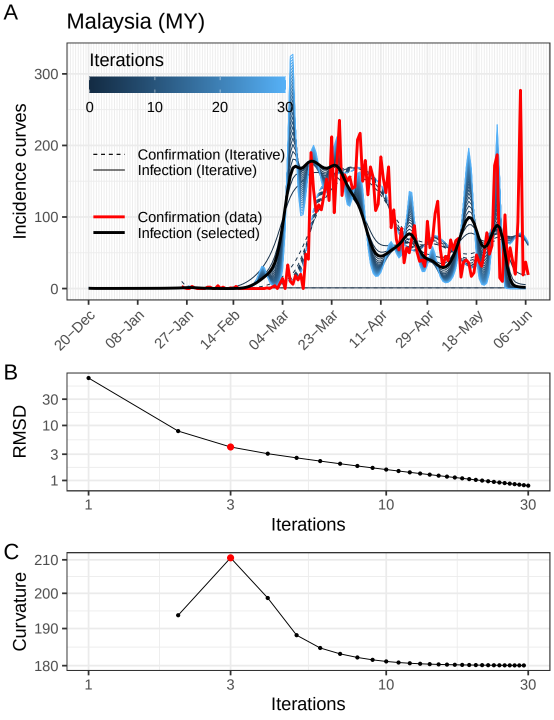

(A) **Deconvolution iterations using uninformative initial condition.** A. The RL deconvolution iterative curves and observed confirmation curve. B: The root-mean-square difference (RMSD) of all iterations. C: The curvature of RMSD. The red dot represents the selected iteration with maximum curvature.

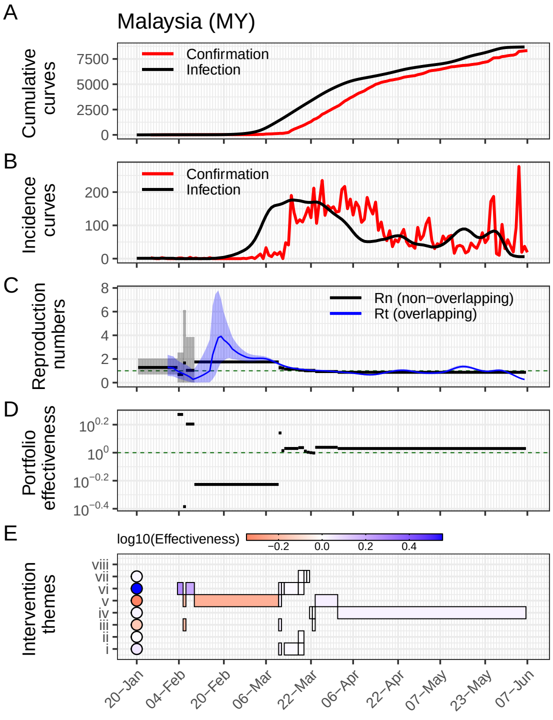

(B) **Estimates using uninformative initial condition.** A. The cumulative curves. B. The incidence curves. C. The instantaneous reproduction numbers R_n and R_t. D. The portfolio effectiveness η_n. E. The intervention $\eta_n^{(m)}$ (in rectangles) and aggregated $\eta^{(m)}$ (in circles) effectiveness.

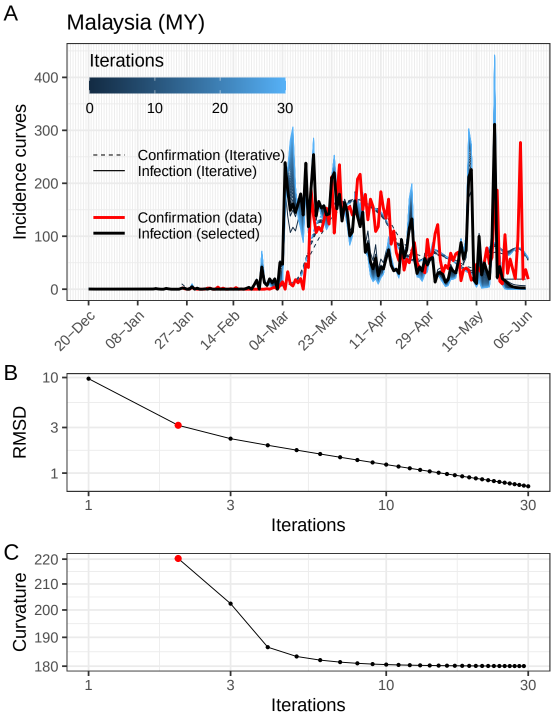

(C) **Deconvolution iterations using informative initial condition.** A. The RL deconvolution iterative curves and observed confirmation curve. B: The root-mean-square difference (RMSD) of all iterations. C: The curvature of RMSD. The red dot represents the selected iteration with maximum curvature.

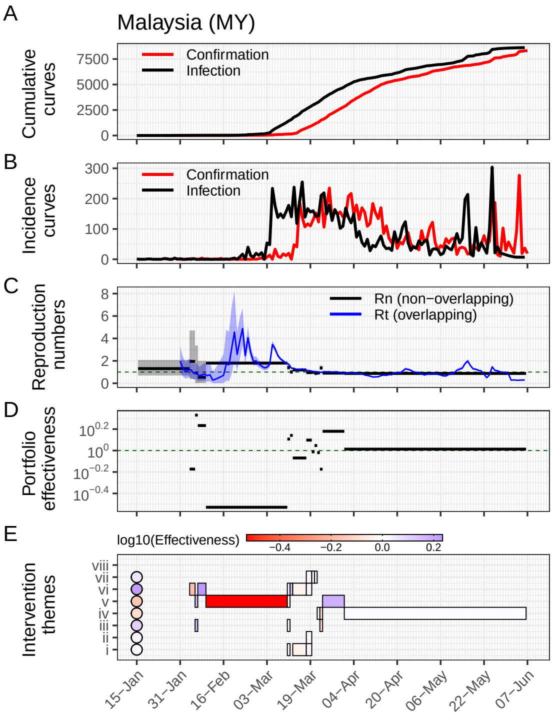

(D) **Estimates using informative initial condition.** A. The cumulative curves. B. The incidence curves. C. The instantaneous reproduction numbers R_n and R_t. D. The portfolio effectiveness η_n. E. The intervention $\eta_n^{(m)}$ (in rectangles) and aggregated $\eta^{(m)}$ (in circles) effectiveness.

FIGURE S29. **The reconstructed epidemic curves and subsequent evaluation of interventions implemented in Malaysia.** Estimation using uninformative (baseline setting, A and B) and informative (alternative setting, C and D) initial condition.

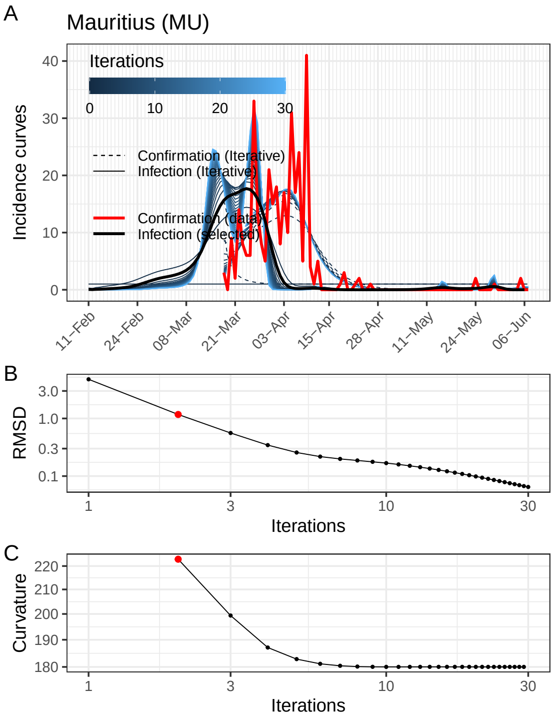

(A) **Deconvolution iterations using uninformative initial condition.** A. The RL deconvolution iterative curves and observed confirmation curve. B: The root-mean-square difference (RMSD) of all iterations. C: The curvature of RMSD. The red dot represents the selected iteration with maximum curvature.

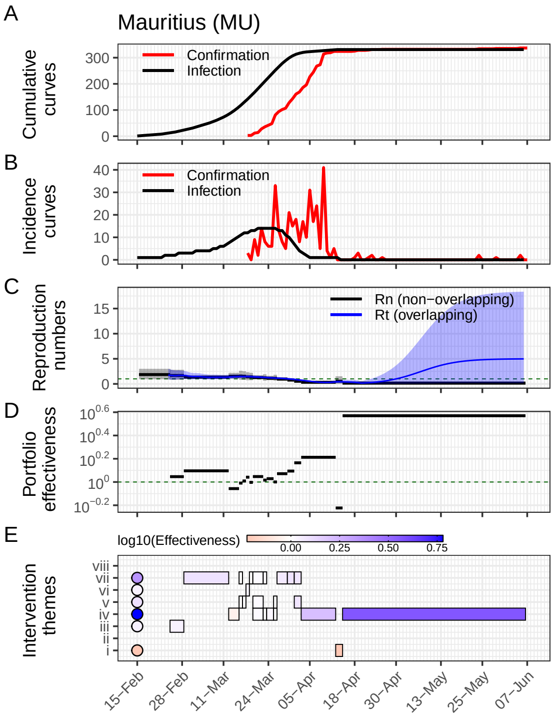

(B) **Estimates using uninformative initial condition.** A. The cumulative curves. B. The incidence curves. C. The instantaneous reproduction numbers R_n and R_t. D. The portfolio effectiveness η_n. E. The intervention $\eta_n^{(m)}$ (in rectangles) and aggregated $\eta^{(m)}$ (in circles) effectiveness.

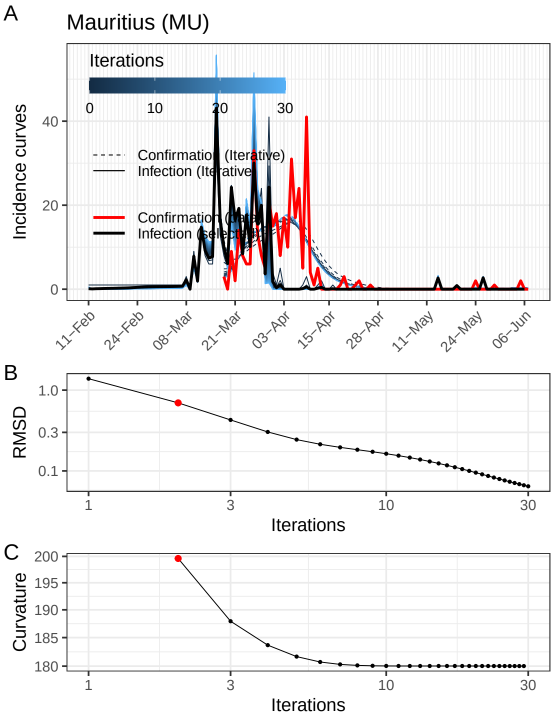

(C) **Deconvolution iterations using informative initial condition.** A. The RL deconvolution iterative curves and observed confirmation curve. B: The root-mean-square difference (RMSD) of all iterations. C: The curvature of RMSD. The red dot represents the selected iteration with maximum curvature.

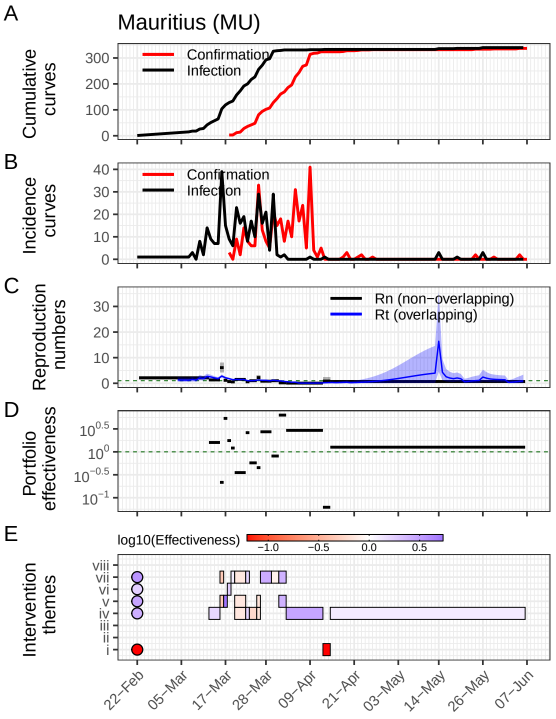

(D) **Estimates using informative initial condition.** A. The cumulative curves. B. The incidence curves. C. The instantaneous reproduction numbers R_n and R_t. D. The portfolio effectiveness η_n. E. The intervention $\eta_n^{(m)}$ (in rectangles) and aggregated $\eta^{(m)}$ (in circles) effectiveness.

FIGURE S30. **The reconstructed epidemic curves and subsequent evaluation of interventions implemented in Mauritius.** Estimation using uninformative (baseline setting, A and B) and informative (alternative setting, C and D) initial condition.

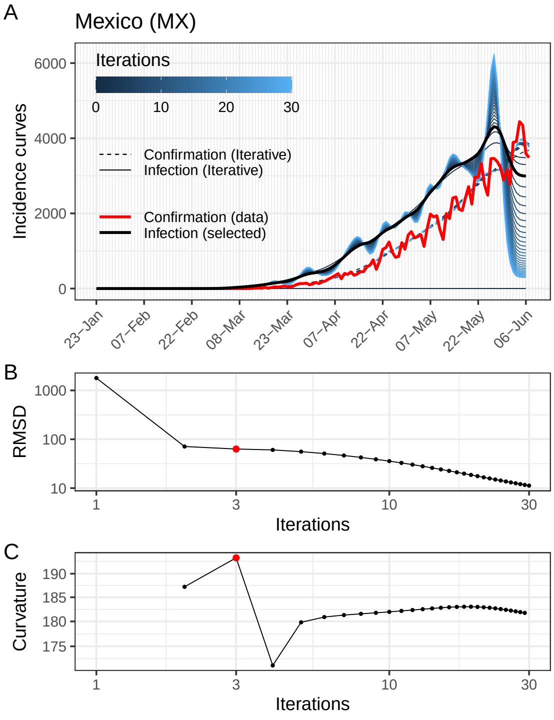

(A) **Deconvolution iterations using uninformative initial condition.** A. The RL deconvolution iterative curves and observed confirmation curve. B: The root-mean-square difference (RMSD) of all iterations. C: The curvature of RMSD. The red dot represents the selected iteration with maximum curvature.

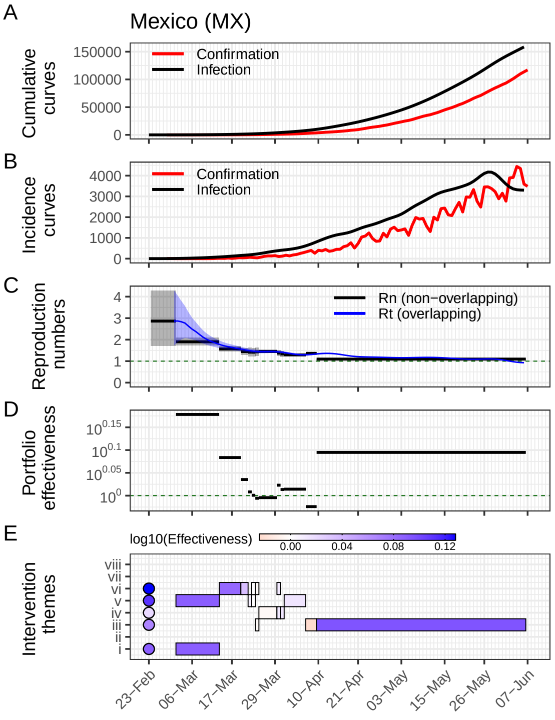

(B) **Estimates using uninformative initial condition.** A. The cumulative curves. B. The incidence curves. C. The instantaneous reproduction numbers R_n and R_t. D. The portfolio effectiveness η_n. E. The intervention $\eta_n^{(m)}$ (in rectangles) and aggregated $\eta^{(m)}$ (in circles) effectiveness.

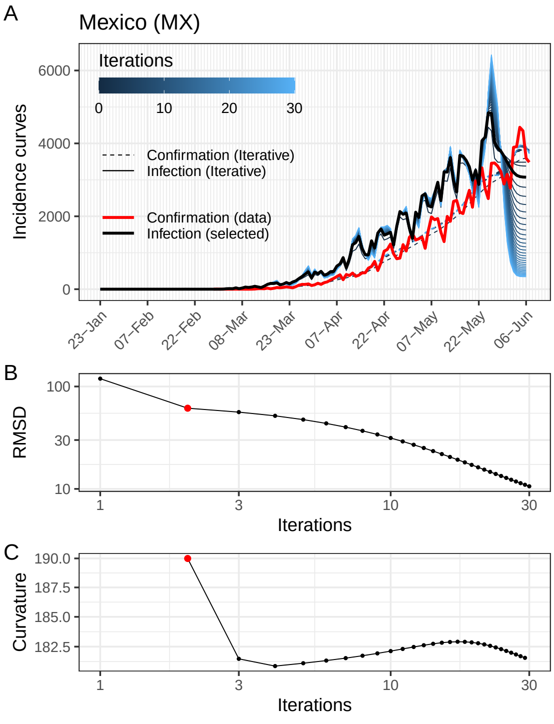

(C) **Deconvolution iterations using informative initial condition.** A. The RL deconvolution iterative curves and observed confirmation curve. B: The root-mean-square difference (RMSD) of all iterations. C: The curvature of RMSD. The red dot represents the selected iteration with maximum curvature.

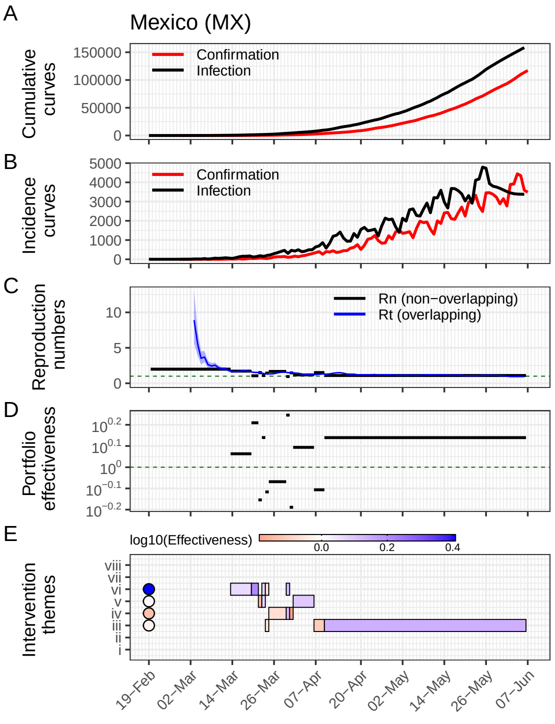

(D) **Estimates using informative initial condition.** A. The cumulative curves. B. The incidence curves. C. The instantaneous reproduction numbers R_n and R_t. D. The portfolio effectiveness η_n. E. The intervention $\eta_n^{(m)}$ (in rectangles) and aggregated $\eta^{(m)}$ (in circles) effectiveness.

Figure S31. **The reconstructed epidemic curves and subsequent evaluation of interventions implemented in Mexico.** Estimation using uninformative (baseline setting, A and B) and informative (alternative setting, C and D) initial condition.

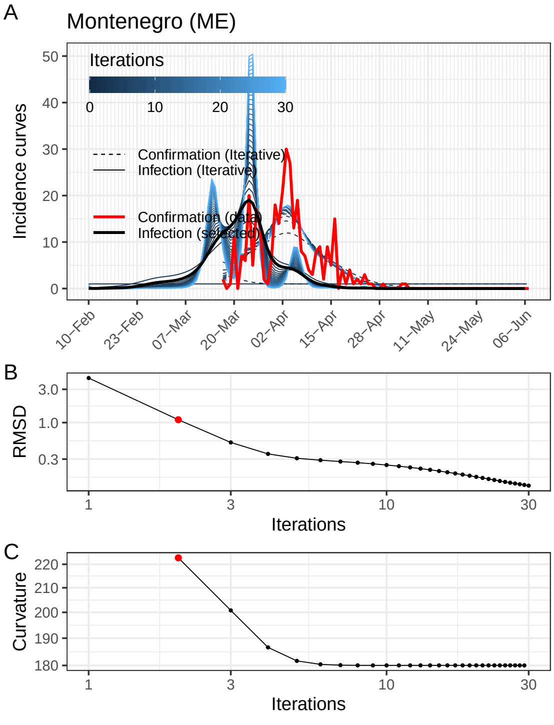

(A) **Deconvolution iterations using uninformative initial condition.** A. The RL deconvolution iterative curves and observed confirmation curve. B: The root-mean-square difference (RMSD) of all iterations. C: The curvature of RMSD. The red dot represents the selected iteration with maximum curvature.

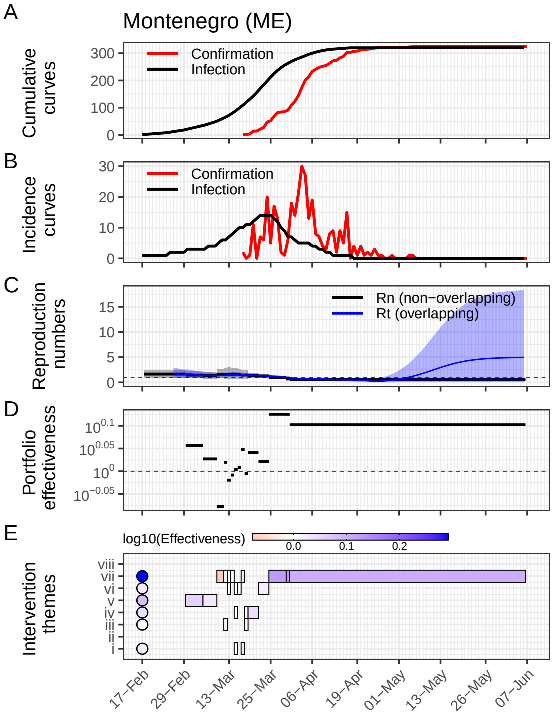

(B) **Estimates using uninformative initial condition.** A. The cumulative curves. B. The incidence curves. C. The instantaneous reproduction numbers R_n and R_t. D. The portfolio effectiveness η_n. E. The intervention $\eta_n^{(m)}$ (in rectangles) and aggregated $\eta^{(m)}$ (in circles) effectiveness.

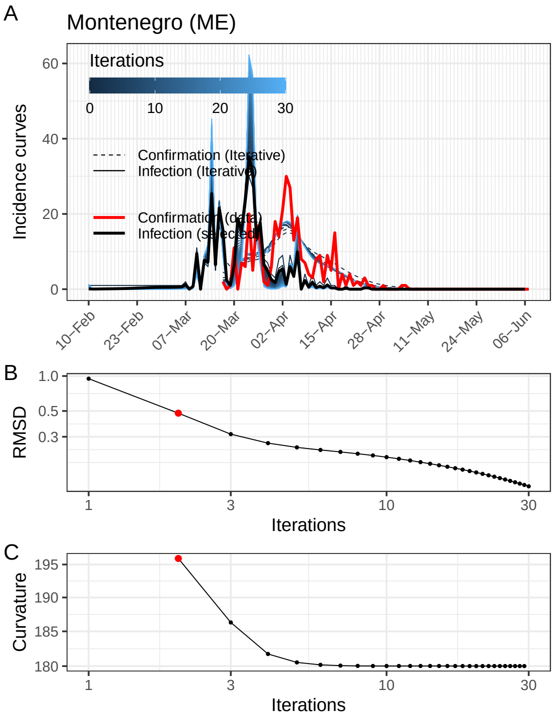

(C) **Deconvolution iterations using informative initial condition.** A. The RL deconvolution iterative curves and observed confirmation curve. B: The root-mean-square difference (RMSD) of all iterations. C: The curvature of RMSD. The red dot represents the selected iteration with maximum curvature.

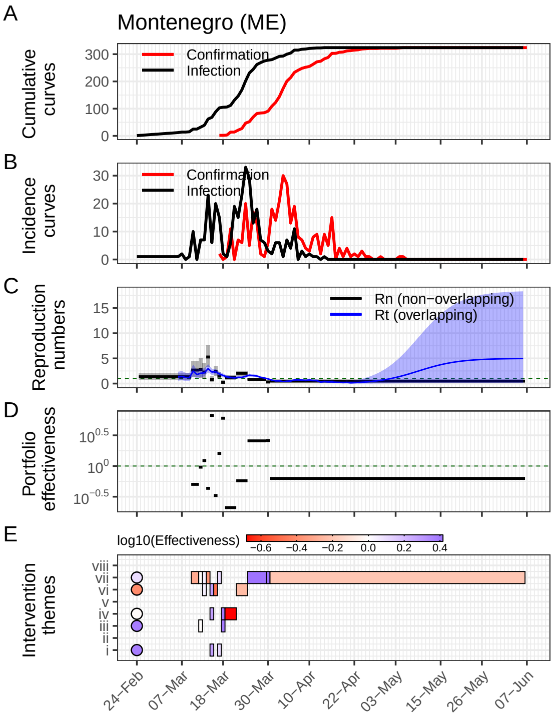

(D) **Estimates using informative initial condition.** A. The cumulative curves. B. The incidence curves. C. The instantaneous reproduction numbers R_n and R_t. D. The portfolio effectiveness η_n. E. The intervention $\eta_n^{(m)}$ (in rectangles) and aggregated $\eta^{(m)}$ (in circles) effectiveness.

FIGURE S32. **The reconstructed epidemic curves and subsequent evaluation of interventions implemented in Montenegro.** Estimation using uninformative (baseline setting, A and B) and informative (alternative setting, C and D) initial condition.

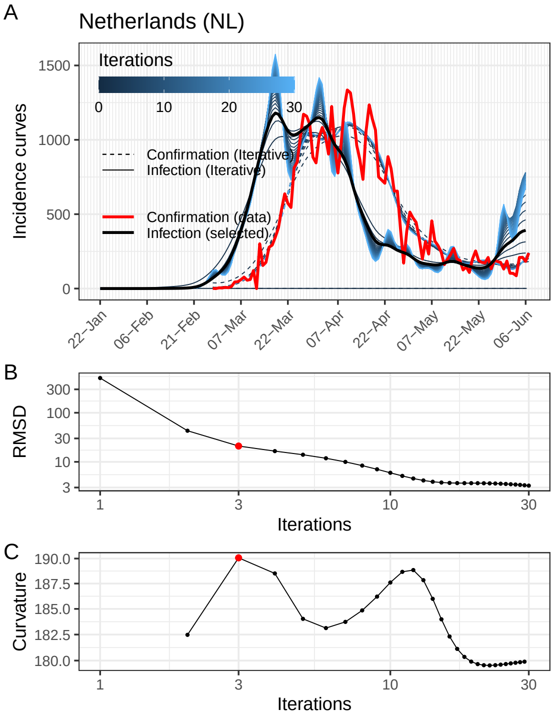

(A) **Deconvolution iterations using uninformative initial condition.** A. The RL deconvolution iterative curves and observed confirmation curve. B: The root-mean-square difference (RMSD) of all iterations. C: The curvature of RMSD. The red dot represents the selected iteration with maximum curvature.

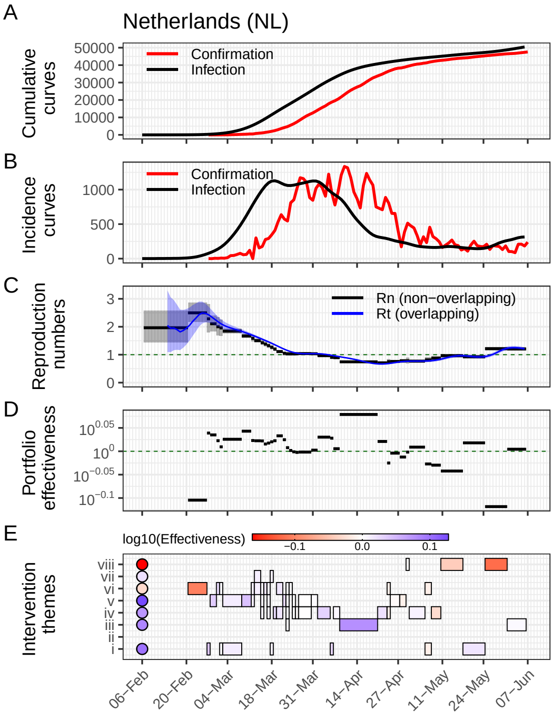

(B) **Estimates using uninformative initial condition.** A. The cumulative curves. B. The incidence curves. C. The instantaneous reproduction numbers R_n and R_t. D. The portfolio effectiveness η_n. E. The intervention $\eta_n^{(m)}$ (in rectangles) and aggregated $\eta^{(m)}$ (in circles) effectiveness.

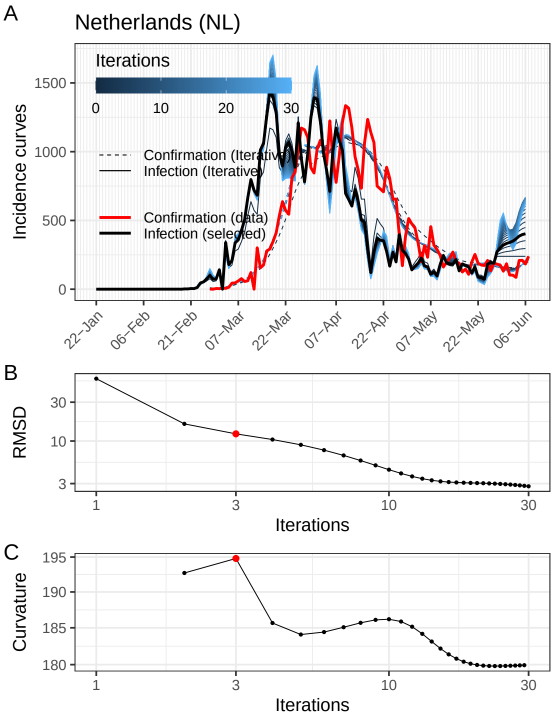

(C) **Deconvolution iterations using informative initial condition.** A. The RL deconvolution iterative curves and observed confirmation curve. B: The root-mean-square difference (RMSD) of all iterations. C: The curvature of RMSD. The red dot represents the selected iteration with maximum curvature.

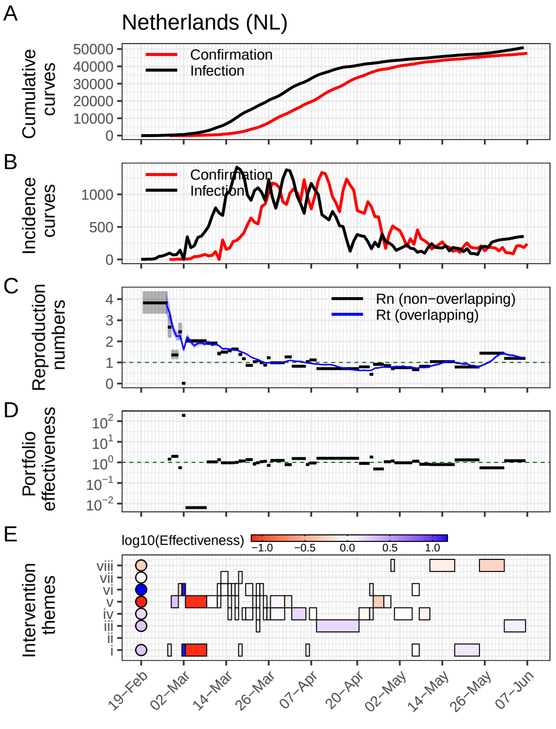

(D) **Estimates using informative initial condition.** A. The cumulative curves. B. The incidence curves. C. The instantaneous reproduction numbers R_n and R_t. D. The portfolio effectiveness η_n. E. The intervention $\eta_n^{(m)}$ (in rectangles) and aggregated $\eta^{(m)}$ (in circles) effectiveness.

FIGURE S33. **The reconstructed epidemic curves and subsequent evaluation of interventions implemented in Netherlands.** Estimation using uninformative (baseline setting, A and B) and informative (alternative setting, C and D) initial condition.

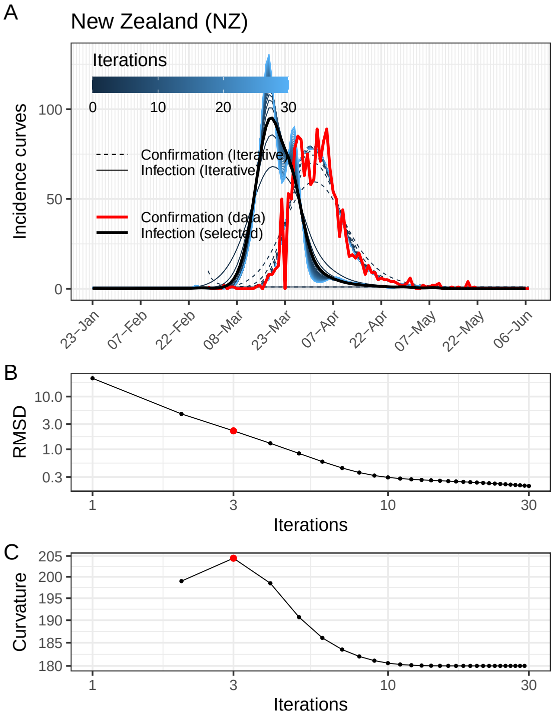

(A) **Deconvolution iterations using uninformative initial condition.** A. The RL deconvolution iterative curves and observed confirmation curve. B: The root-mean-square difference (RMSD) of all iterations. C: The curvature of RMSD. The red dot represents the selected iteration with maximum curvature.

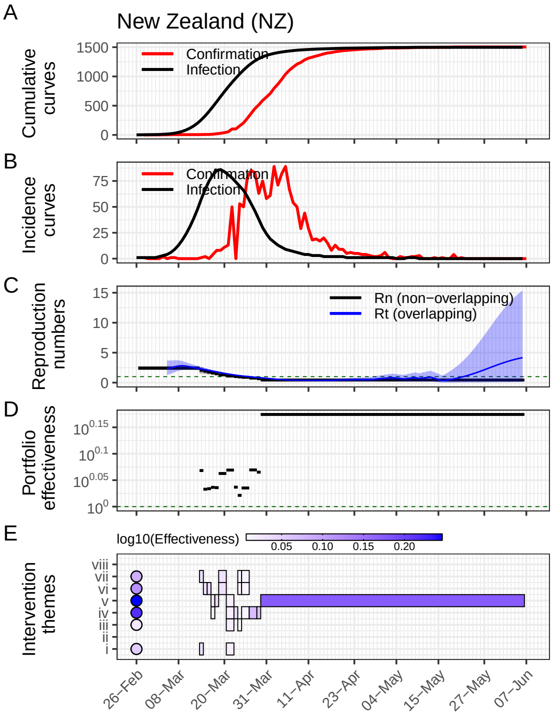

(B) **Estimates using uninformative initial condition.** A. The cumulative curves. B. The incidence curves. C. The instantaneous reproduction numbers R_n and R_t. D. The portfolio effectiveness η_n. E. The intervention $\eta_n^{(m)}$ (in rectangles) and aggregated $\eta^{(m)}$ (in circles) effectiveness.

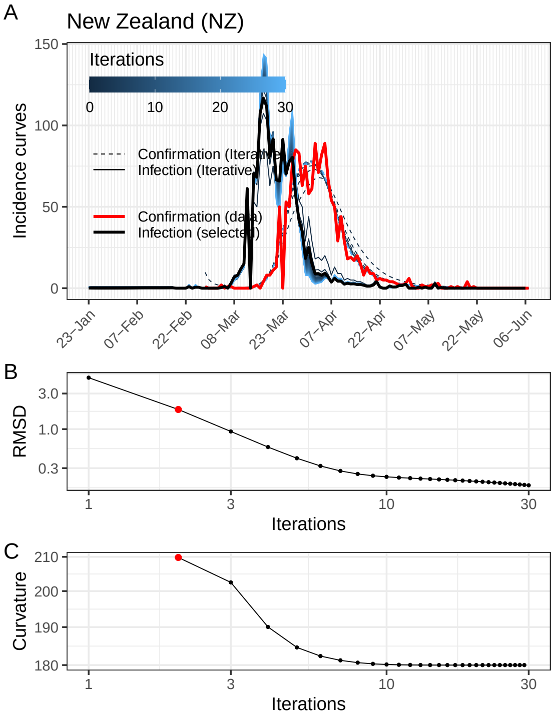

(C) **Deconvolution iterations using informative initial condition.** A. The RL deconvolution iterative curves and observed confirmation curve. B: The root-mean-square difference (RMSD) of all iterations. C: The curvature of RMSD. The red dot represents the selected iteration with maximum curvature.

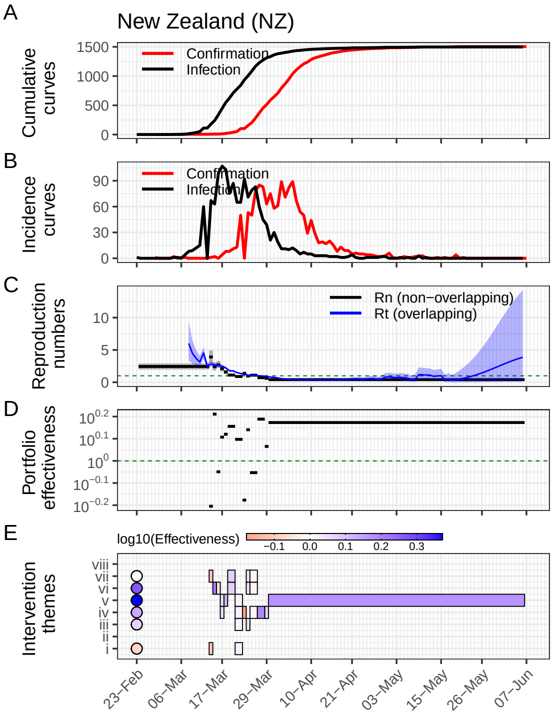

(D) **Estimates using informative initial condition.** A. The cumulative curves. B. The incidence curves. C. The instantaneous reproduction numbers R_n and R_t. D. The portfolio effectiveness η_n. E. The intervention $\eta_n^{(m)}$ (in rectangles) and aggregated $\eta^{(m)}$ (in circles) effectiveness.

FIGURE S34. **The reconstructed epidemic curves and subsequent evaluation of interventions implemented in New Zealand.** Estimation using uninformative (baseline setting, A and B) and informative (alternative setting, C and D) initial condition.

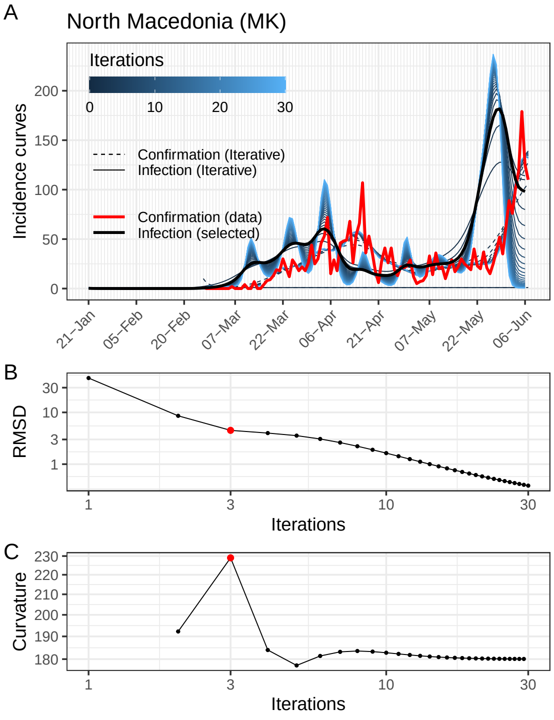

(A) **Deconvolution iterations using uninformative initial condition.** A. The RL deconvolution iterative curves and observed confirmation curve. B: The root-mean-square difference (RMSD) of all iterations. C: The curvature of RMSD. The red dot represents the selected iteration with maximum curvature.

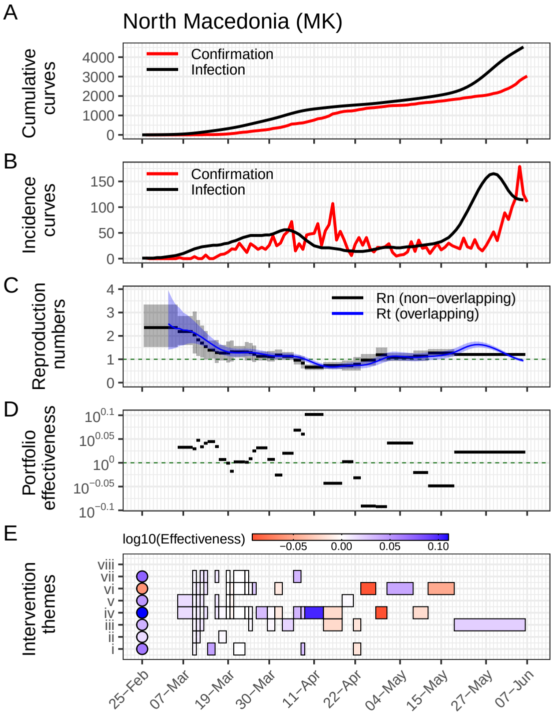

(B) **Estimates using uninformative initial condition.** A. The cumulative curves. B. The incidence curves. C. The instantaneous reproduction numbers R_n and R_t. D. The portfolio effectiveness η_n. E. The intervention $\eta_n^{(m)}$ (in rectangles) and aggregated $\eta^{(m)}$ (in circles) effectiveness.

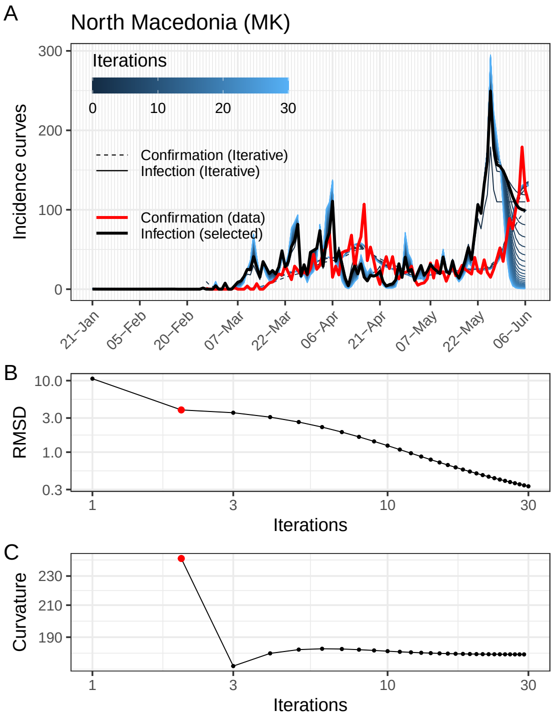

(C) **Deconvolution iterations using informative initial condition.** A. The RL deconvolution iterative curves and observed confirmation curve. B: The root-mean-square difference (RMSD) of all iterations. C: The curvature of RMSD. The red dot represents the selected iteration with maximum curvature.

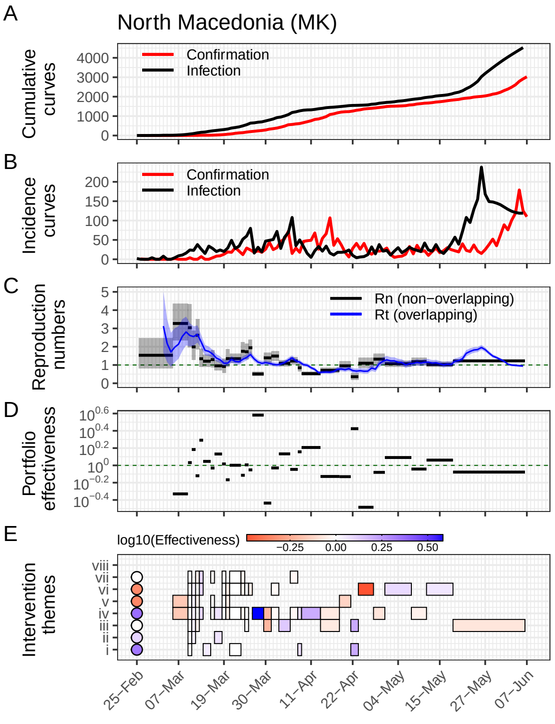

(D) **Estimates using informative initial condition.** A. The cumulative curves. B. The incidence curves. C. The instantaneous reproduction numbers R_n and R_t. D. The portfolio effectiveness η_n. E. The intervention $\eta_n^{(m)}$ (in rectangles) and aggregated $\eta^{(m)}$ (in circles) effectiveness.

FIGURE S35. **The reconstructed epidemic curves and subsequent evaluation of interventions implemented in North Macedonia.** Estimation using uninformative (baseline setting, A and B) and informative (alternative setting, C and D) initial condition.

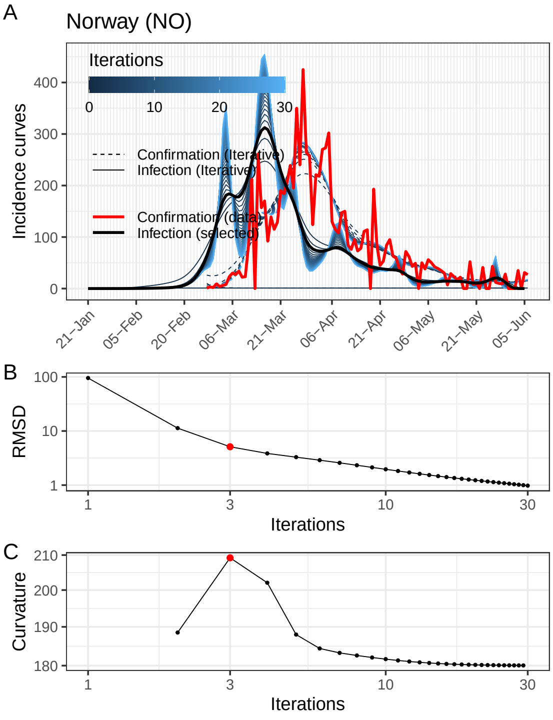

(A) **Deconvolution iterations using uninformative initial condition.** A. The RL deconvolution iterative curves and observed confirmation curve. B: The root-mean-square difference (RMSD) of all iterations. C: The curvature of RMSD. The red dot represents the selected iteration with maximum curvature.

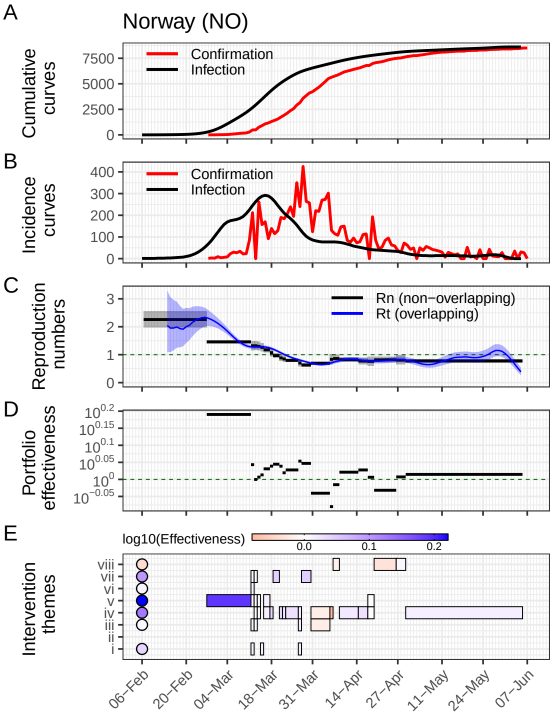

(B) **Estimates using uninformative initial condition.** A. The cumulative curves. B. The incidence curves. C. The instantaneous reproduction numbers R_n and R_t. D. The portfolio effectiveness η_n. E. The intervention $\eta_n^{(m)}$ (in rectangles) and aggregated $\eta^{(m)}$ (in circles) effectiveness.

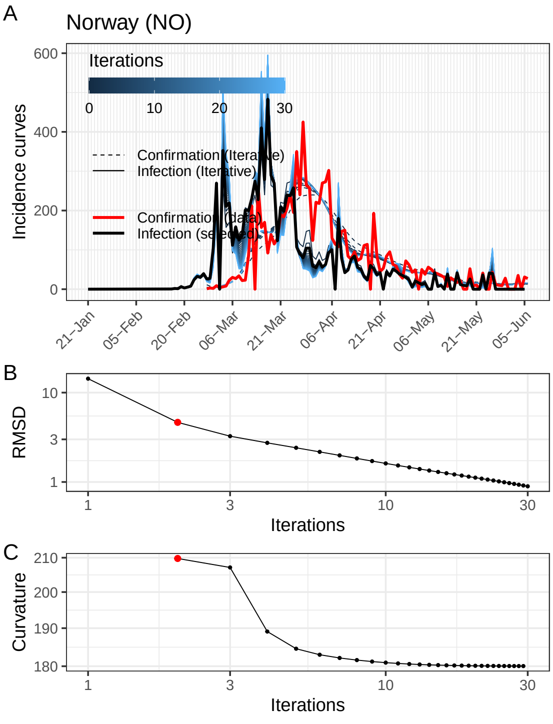

(C) **Deconvolution iterations using informative initial condition.** A. The RL deconvolution iterative curves and observed confirmation curve. B: The root-mean-square difference (RMSD) of all iterations. C: The curvature of RMSD. The red dot represents the selected iteration with maximum curvature.

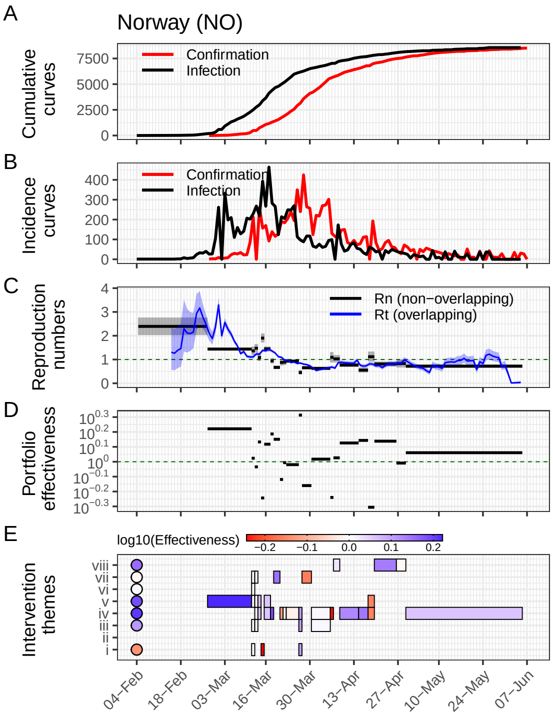

(D) **Estimates using informative initial condition.** A. The cumulative curves. B. The incidence curves. C. The instantaneous reproduction numbers R_n and R_t. D. The portfolio effectiveness η_n. E. The intervention $\eta_n^{(m)}$ (in rectangles) and aggregated $\eta^{(m)}$ (in circles) effectiveness.

FIGURE S36. **The reconstructed epidemic curves and subsequent evaluation of interventions implemented in Norway.** Estimation using uninformative (baseline setting, A and B) and informative (alternative setting, C and D) initial condition.

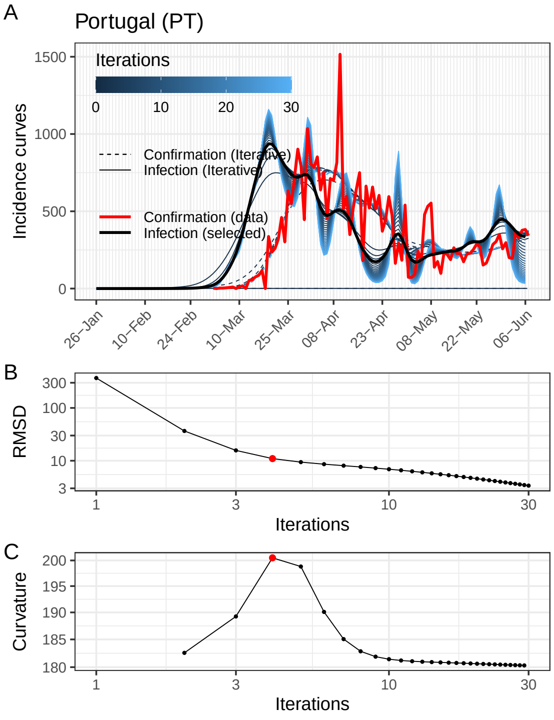

(A) **Deconvolution iterations using uninformative initial condition.** A. The RL deconvolution iterative curves and observed confirmation curve. B: The root-mean-square difference (RMSD) of all iterations. C: The curvature of RMSD. The red dot represents the selected iteration with maximum curvature.

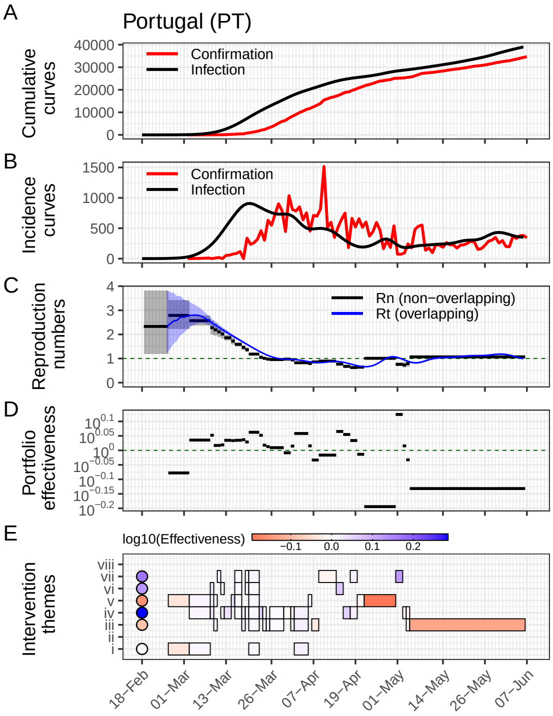

(B) **Estimates using uninformative initial condition.** A. The cumulative curves. B. The incidence curves. C. The instantaneous reproduction numbers R_n and R_t. D. The portfolio effectiveness η_n. E. The intervention $\eta_n^{(m)}$ (in rectangles) and aggregated $\eta^{(m)}$ (in circles) effectiveness.

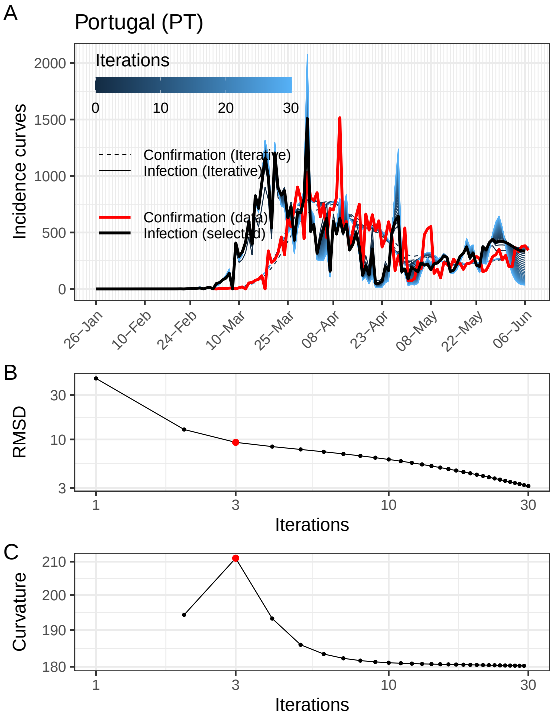

(C) **Deconvolution iterations using informative initial condition.** A. The RL deconvolution iterative curves and observed confirmation curve. B: The root-mean-square difference (RMSD) of all iterations. C: The curvature of RMSD. The red dot represents the selected iteration with maximum curvature.

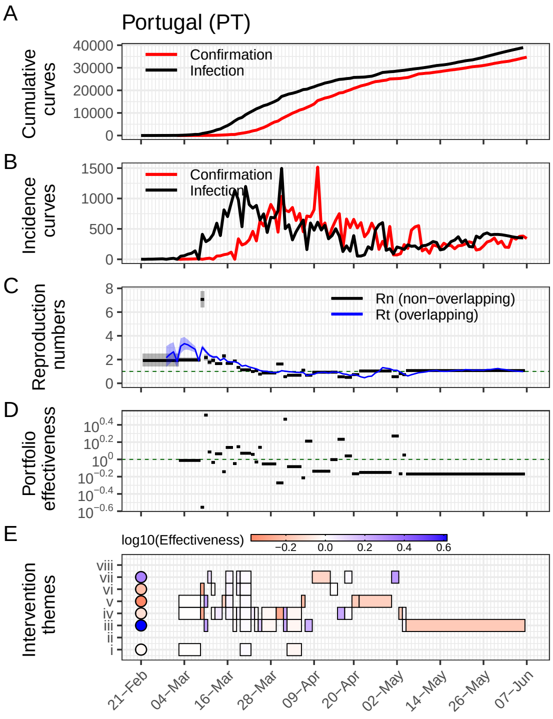

(D) **Estimates using informative initial condition.** A. The cumulative curves. B. The incidence curves. C. The instantaneous reproduction numbers R_n and R_t. D. The portfolio effectiveness η_n. E. The intervention $\eta_n^{(m)}$ (in rectangles) and aggregated $\eta^{(m)}$ (in circles) effectiveness.

FIGURE S37. **The reconstructed epidemic curves and subsequent evaluation of interventions implemented in Portugal.** Estimation using uninformative (baseline setting, A and B) and informative (alternative setting, C and D) initial condition.

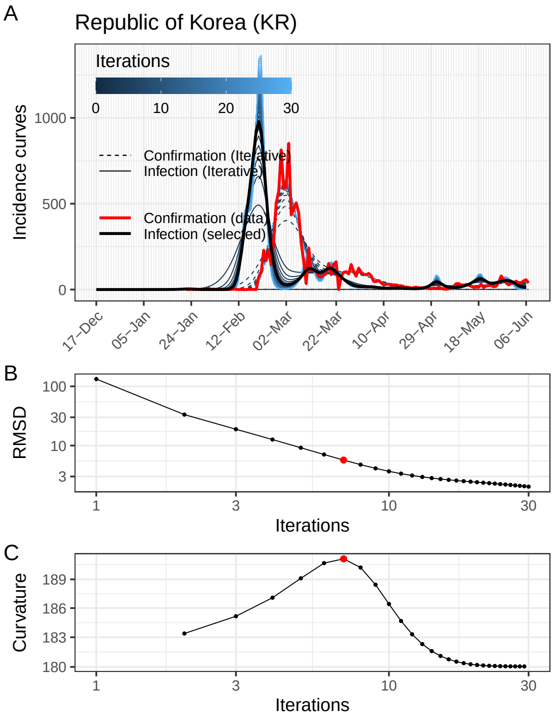

(A) **Deconvolution iterations using uninformative initial condition.** A. The RL deconvolution iterative curves and observed confirmation curve. B: The root-mean-square difference (RMSD) of all iterations. C: The curvature of RMSD. The red dot represents the selected iteration with maximum curvature.

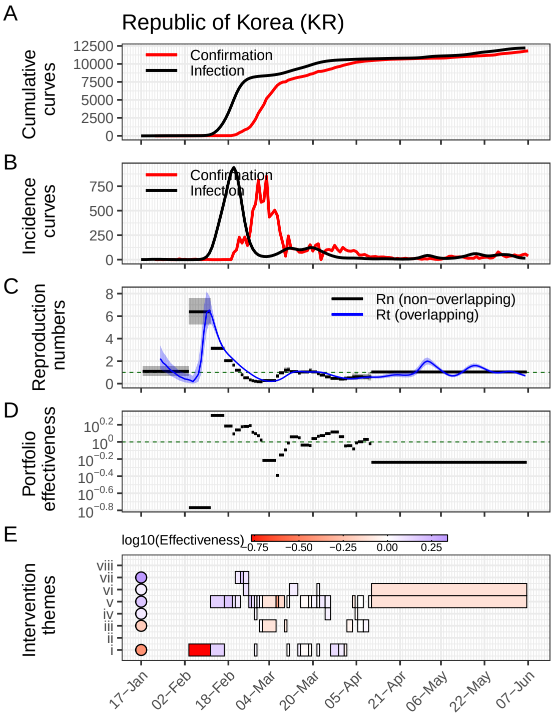

(B) **Estimates using uninformative initial condition.** A. The cumulative curves. B. The incidence curves. C. The instantaneous reproduction numbers R_n and R_t. D. The portfolio effectiveness η_n. E. The intervention $\eta_n^{(m)}$ (in rectangles) and aggregated $\eta^{(m)}$ (in circles) effectiveness.

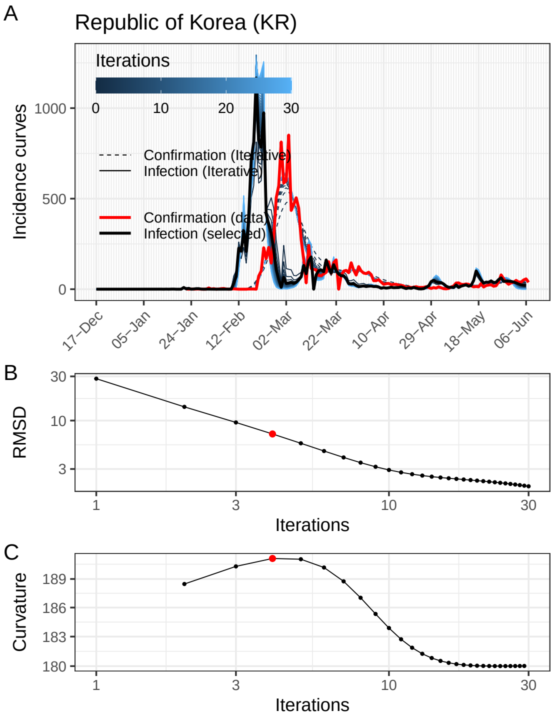

(C) **Deconvolution iterations using informative initial condition.** A. The RL deconvolution iterative curves and observed confirmation curve. B: The root-mean-square difference (RMSD) of all iterations. C: The curvature of RMSD. The red dot represents the selected iteration with maximum curvature.

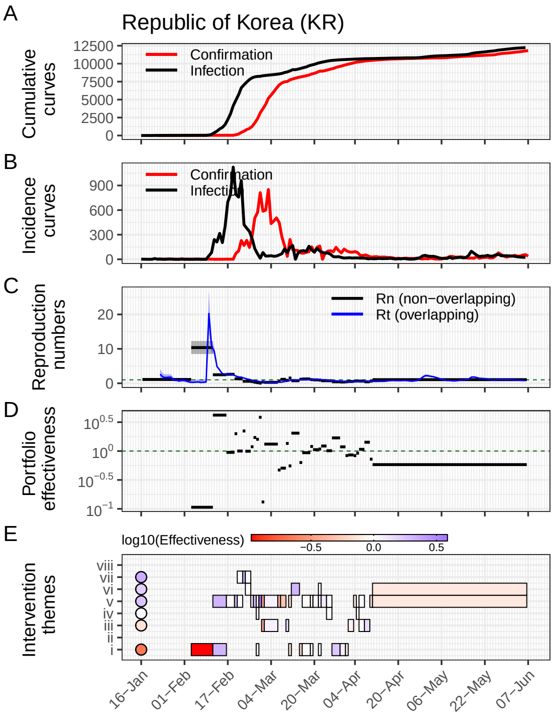

(D) **Estimates using informative initial condition.** A. The cumulative curves. B. The incidence curves. C. The instantaneous reproduction numbers R_n and R_t. D. The portfolio effectiveness η_n. E. The intervention $\eta_n^{(m)}$ (in rectangles) and aggregated $\eta^{(m)}$ (in circles) effectiveness.

Figure S38. **The reconstructed epidemic curves and subsequent evaluation of interventions implemented in Republic of Korea.** Estimation using uninformative (baseline setting, A and B) and informative (alternative setting, C and D) initial condition.

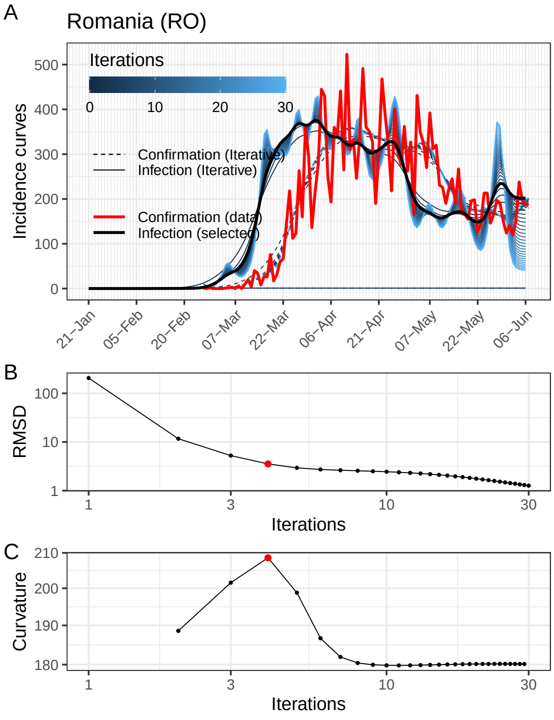

(A) **Deconvolution iterations using uninformative initial condition.** A. The RL deconvolution iterative curves and observed confirmation curve. B: The root-mean-square difference (RMSD) of all iterations. C: The curvature of RMSD. The red dot represents the selected iteration with maximum curvature.

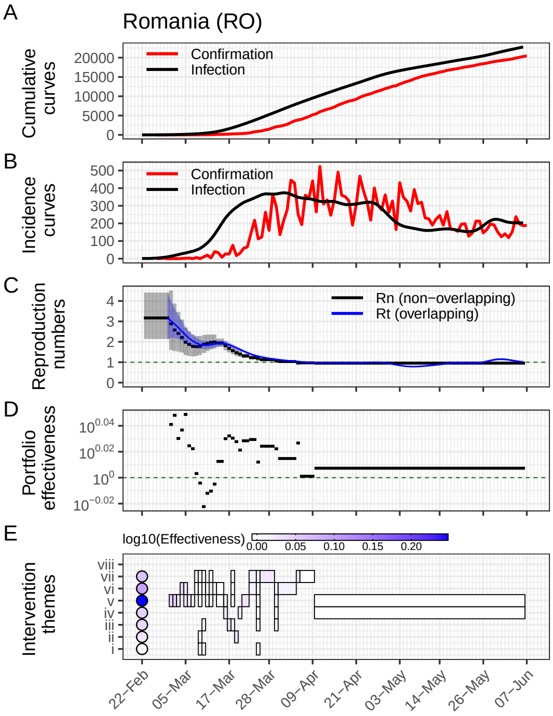

(B) **Estimates using uninformative initial condition.** A. The cumulative curves. B. The incidence curves. C. The instantaneous reproduction numbers R_n and R_t. D. The portfolio effectiveness η_n. E. The intervention $\eta_n^{(m)}$ (in rectangles) and aggregated $\eta^{(m)}$ (in circles) effectiveness.

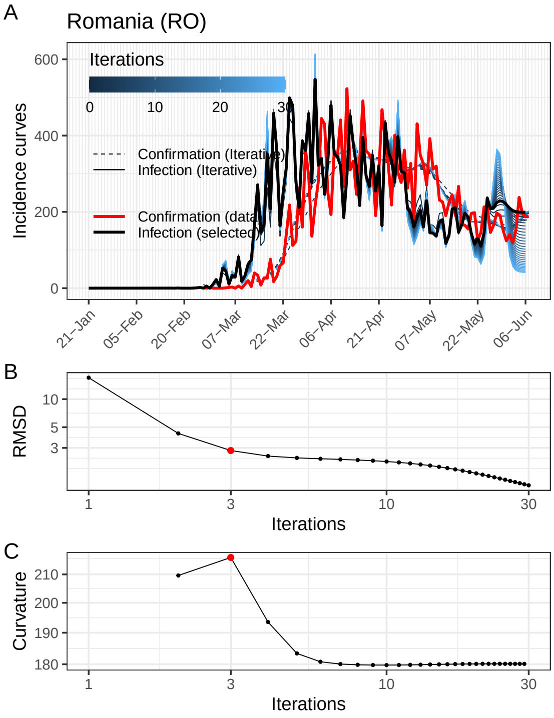

(C) **Deconvolution iterations using informative initial condition.** A. The RL deconvolution iterative curves and observed confirmation curve. B: The root-mean-square difference (RMSD) of all iterations. C: The curvature of RMSD. The red dot represents the selected iteration with maximum curvature.

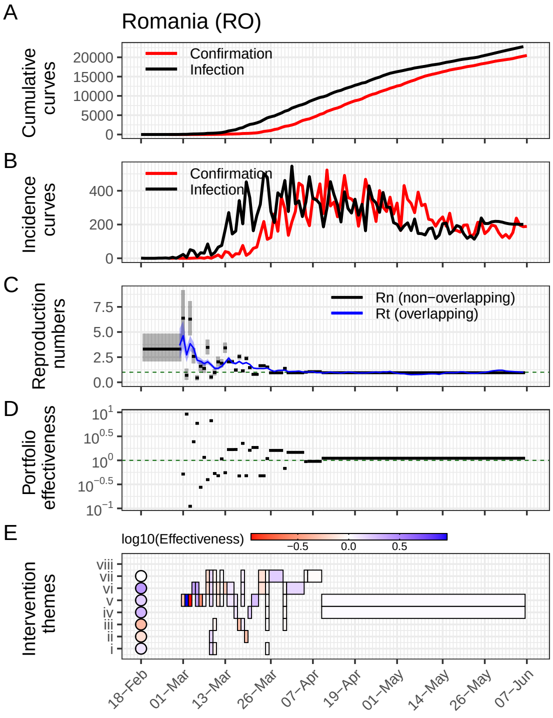

(D) **Estimates using informative initial condition.** A. The cumulative curves. B. The incidence curves. C. The instantaneous reproduction numbers R_n and R_t. D. The portfolio effectiveness η_n. E. The intervention $\eta_n^{(m)}$ (in rectangles) and aggregated $\eta^{(m)}$ (in circles) effectiveness.

FIGURE S39. **The reconstructed epidemic curves and subsequent evaluation of interventions implemented in Romania.** Estimation using uninformative (baseline setting, A and B) and informative (alternative setting, C and D) initial condition.

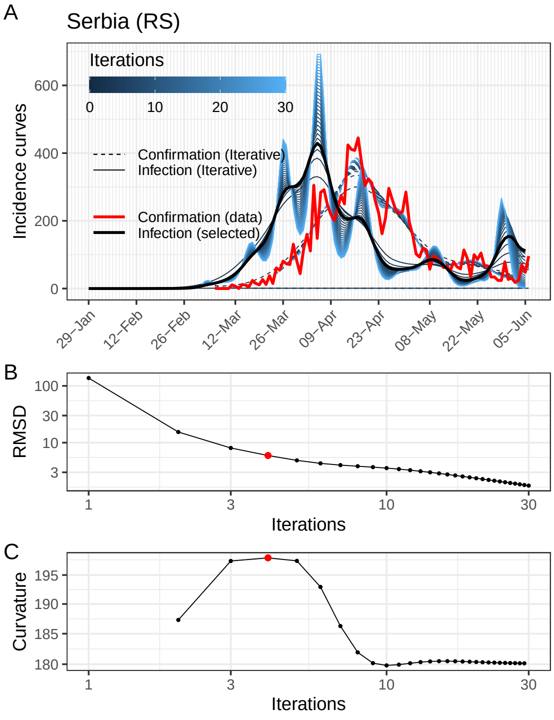

(A) **Deconvolution iterations using uninformative initial condition.** A. The RL deconvolution iterative curves and observed confirmation curve. B: The root-mean-square difference (RMSD) of all iterations. C: The curvature of RMSD. The red dot represents the selected iteration with maximum curvature.

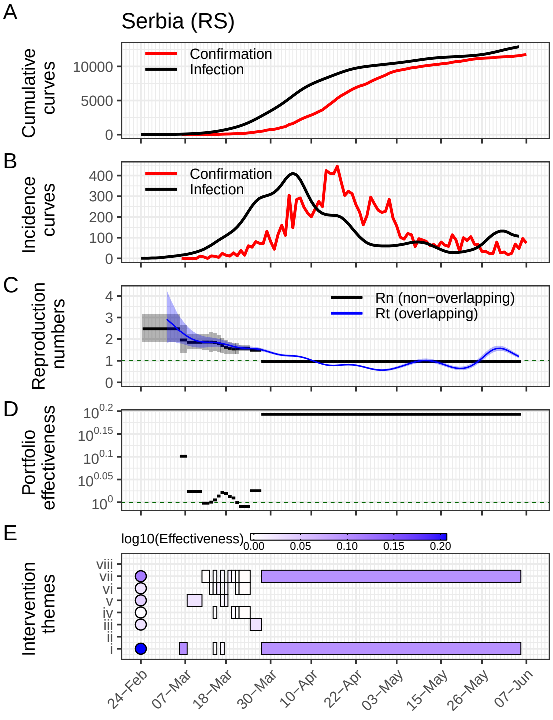

(B) **Estimates using uninformative initial condition.** A. The cumulative curves. B. The incidence curves. C. The instantaneous reproduction numbers R_n and R_t. D. The portfolio effectiveness η_n. E. The intervention $\eta_n^{(m)}$ (in rectangles) and aggregated $\eta^{(m)}$ (in circles) effectiveness.

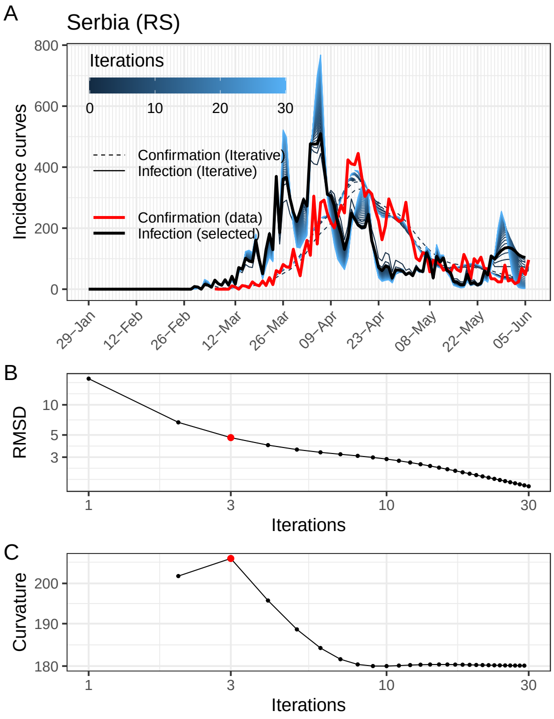

(C) **Deconvolution iterations using informative initial condition.** A. The RL deconvolution iterative curves and observed confirmation curve. B: The root-mean-square difference (RMSD) of all iterations. C: The curvature of RMSD. The red dot represents the selected iteration with maximum curvature.

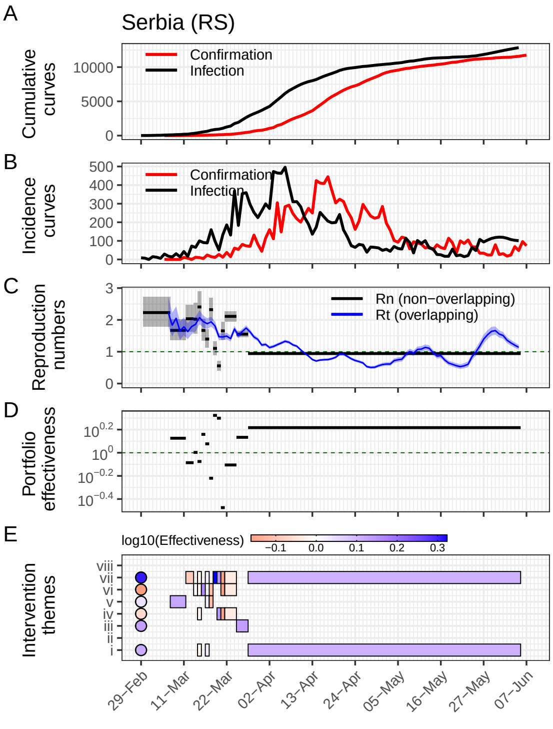

(D) **Estimates using informative initial condition.** A. The cumulative curves. B. The incidence curves. C. The instantaneous reproduction numbers R_n and R_t. D. The portfolio effectiveness η_n. E. The intervention $\eta_n^{(m)}$ (in rectangles) and aggregated $\eta^{(m)}$ (in circles) effectiveness.

Figure S40. **The reconstructed epidemic curves and subsequent evaluation of interventions implemented in Serbia.** Estimation using uninformative (baseline setting, A and B) and informative (alternative setting, C and D) initial condition.

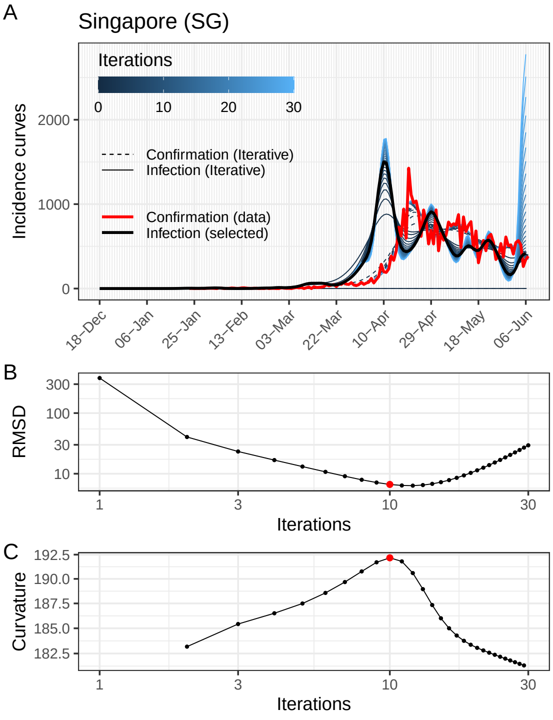

(A) **Deconvolution iterations using uninformative initial condition.** A. The RL deconvolution iterative curves and observed confirmation curve. B: The root-mean-square difference (RMSD) of all iterations. C: The curvature of RMSD. The red dot represents the selected iteration with maximum curvature.

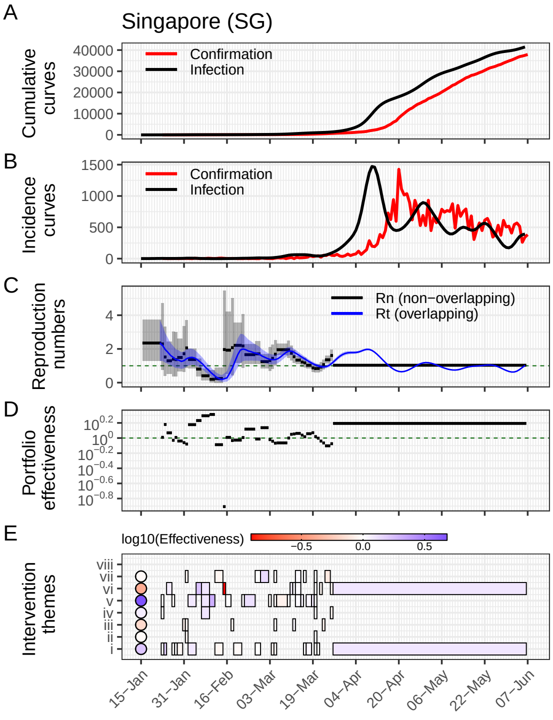

(B) **Estimates using uninformative initial condition.** A. The cumulative curves. B. The incidence curves. C. The instantaneous reproduction numbers R_n and R_t. D. The portfolio effectiveness η_n. E. The intervention $\eta_n^{(m)}$ (in rectangles) and aggregated $\eta^{(m)}$ (in circles) effectiveness.

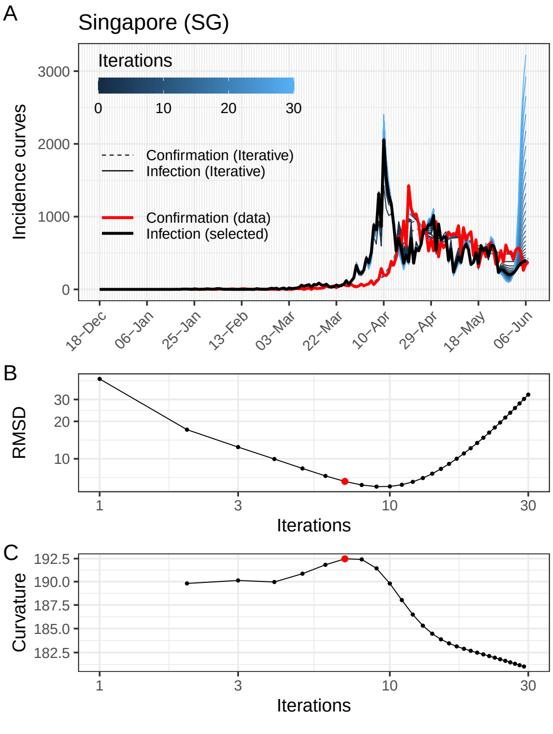

(C) **Deconvolution iterations using informative initial condition.** A. The RL deconvolution iterative curves and observed confirmation curve. B: The root-mean-square difference (RMSD) of all iterations. C: The curvature of RMSD. The red dot represents the selected iteration with maximum curvature.

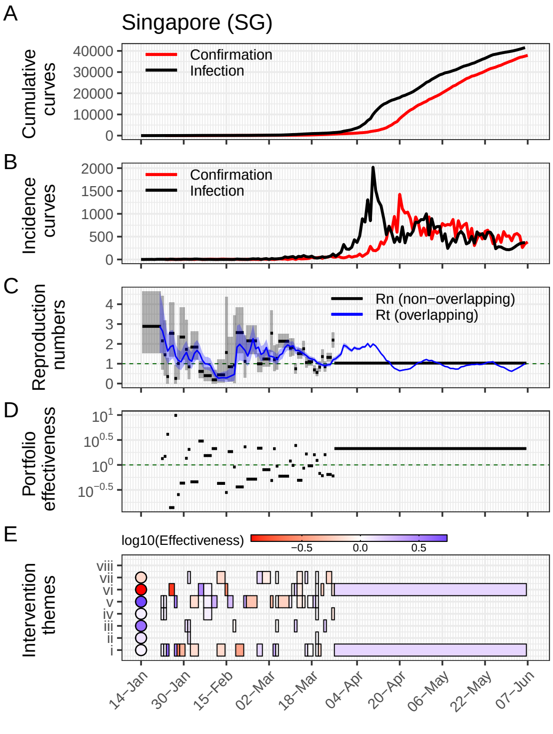

(D) **Estimates using informative initial condition.** A. The cumulative curves. B. The incidence curves. C. The instantaneous reproduction numbers R_n and R_t. D. The portfolio effectiveness η_n. E. The intervention $\eta_n^{(m)}$ (in rectangles) and aggregated $\eta^{(m)}$ (in circles) effectiveness.

Figure S41. **The reconstructed epidemic curves and subsequent evaluation of interventions implemented in Singapore.** Estimation using uninformative (baseline setting, A and B) and informative (alternative setting, C and D) initial condition.

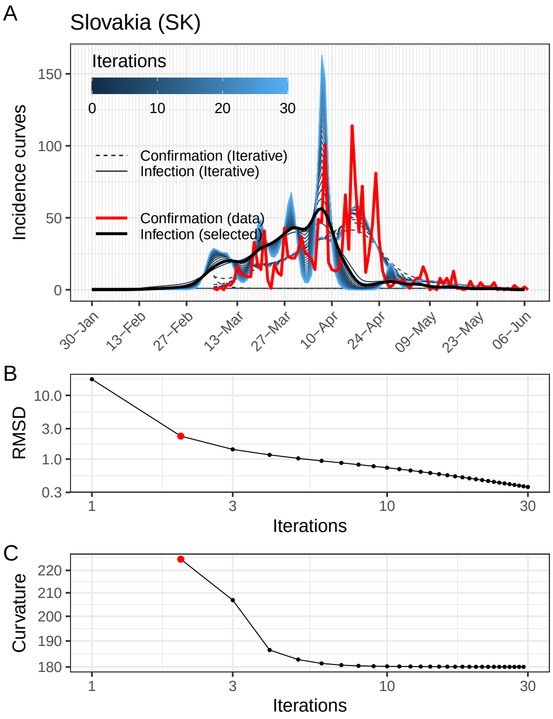

(A) **Deconvolution iterations using uninformative initial condition.** A. The RL deconvolution iterative curves and observed confirmation curve. B: The root-mean-square difference (RMSD) of all iterations. C: The curvature of RMSD. The red dot represents the selected iteration with maximum curvature.

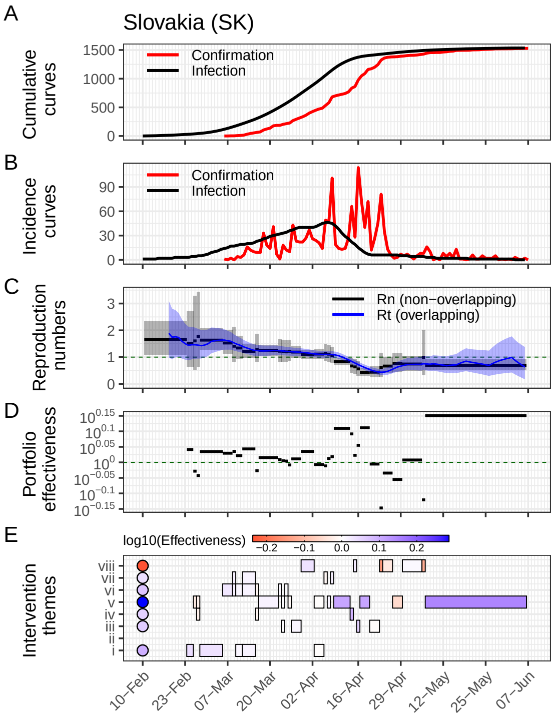

(B) **Estimates using uninformative initial condition.** A. The cumulative curves. B. The incidence curves. C. The instantaneous reproduction numbers R_n and R_t. D. The portfolio effectiveness η_n. E. The intervention $\eta_n^{(m)}$ (in rectangles) and aggregated $\eta^{(m)}$ (in circles) effectiveness.

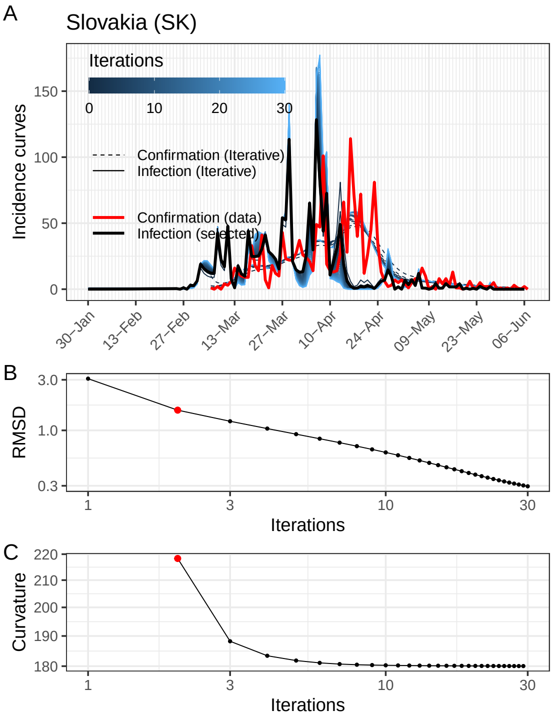

(C) **Deconvolution iterations using informative initial condition.** A. The RL deconvolution iterative curves and observed confirmation curve. B: The root-mean-square difference (RMSD) of all iterations. C: The curvature of RMSD. The red dot represents the selected iteration with maximum curvature.

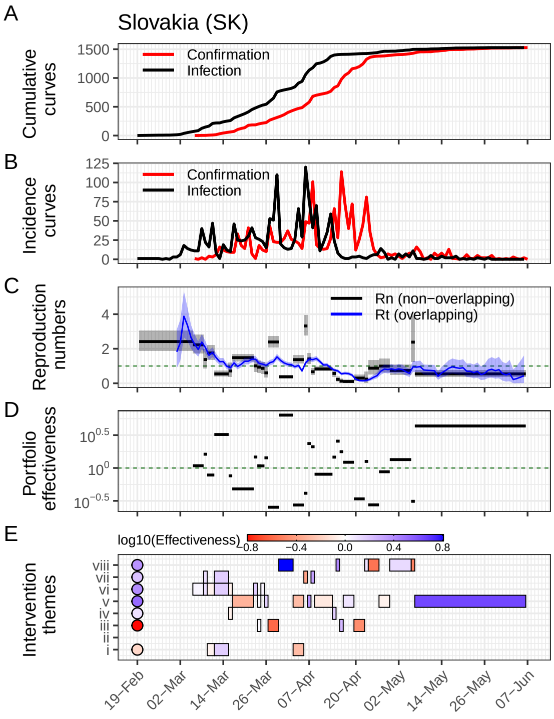

(D) **Estimates using informative initial condition.** A. The cumulative curves. B. The incidence curves. C. The instantaneous reproduction numbers R_n and R_t. D. The portfolio effectiveness η_n. E. The intervention $\eta_n^{(m)}$ (in rectangles) and aggregated $\eta^{(m)}$ (in circles) effectiveness.

Figure S42. **The reconstructed epidemic curves and subsequent evaluation of interventions implemented in Slovakia.** Estimation using uninformative (baseline setting, A and B) and informative (alternative setting, C and D) initial condition.

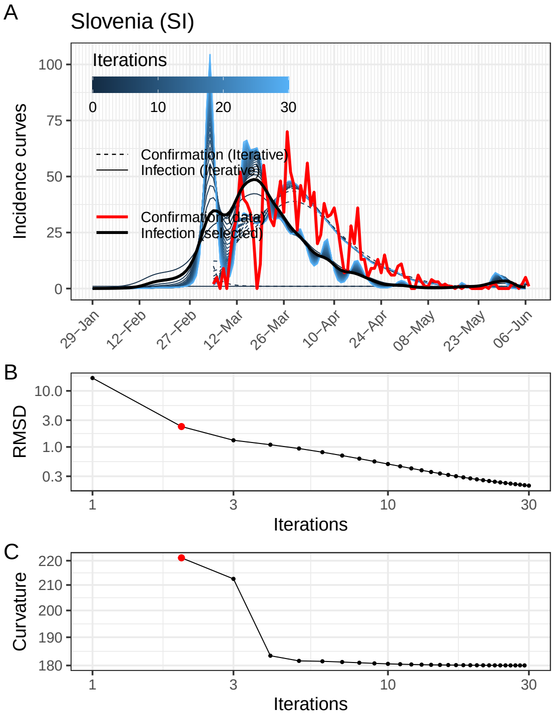

(A) **Deconvolution iterations using uninformative initial condition.** A. The RL deconvolution iterative curves and observed confirmation curve. B: The root-mean-square difference (RMSD) of all iterations. C: The curvature of RMSD. The red dot represents the selected iteration with maximum curvature.

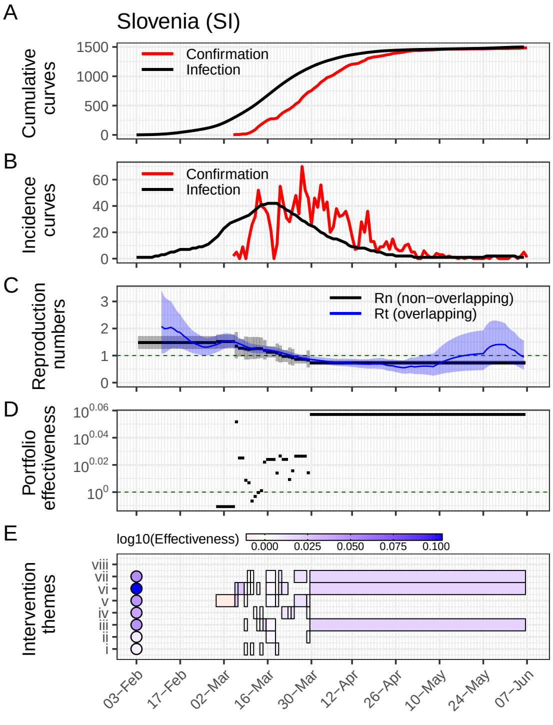

(B) **Estimates using uninformative initial condition.** A. The cumulative curves. B. The incidence curves. C. The instantaneous reproduction numbers R_n and R_t. D. The portfolio effectiveness η_n. E. The intervention $\eta_n^{(m)}$ (in rectangles) and aggregated $\eta^{(m)}$ (in circles) effectiveness.

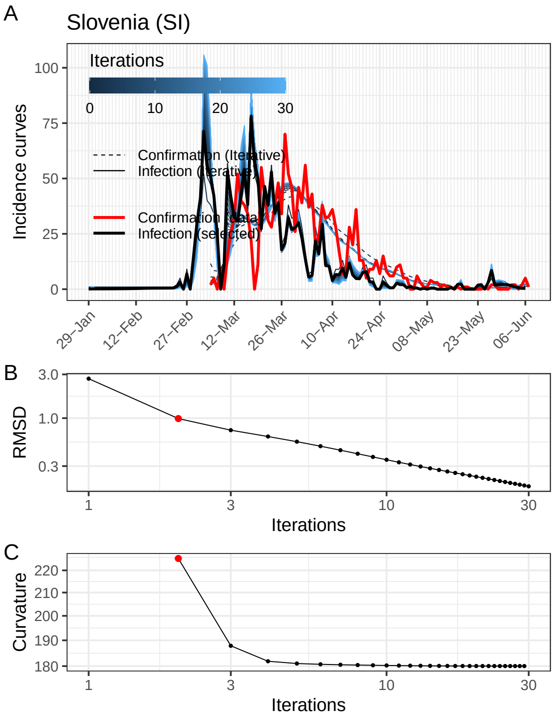

(C) **Deconvolution iterations using informative initial condition.** A. The RL deconvolution iterative curves and observed confirmation curve. B: The root-mean-square difference (RMSD) of all iterations. C: The curvature of RMSD. The red dot represents the selected iteration with maximum curvature.

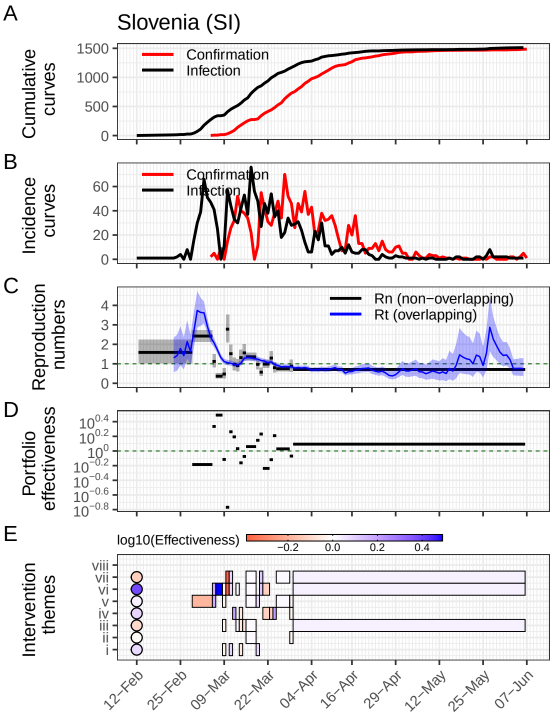

(D) **Estimates using informative initial condition.** A. The cumulative curves. B. The incidence curves. C. The instantaneous reproduction numbers R_n and R_t. D. The portfolio effectiveness η_n. E. The intervention $\eta_n^{(m)}$ (in rectangles) and aggregated $\eta^{(m)}$ (in circles) effectiveness.

FIGURE S43. **The reconstructed epidemic curves and subsequent evaluation of interventions implemented in Slovenia.** Estimation using uninformative (baseline setting, A and B) and informative (alternative setting, C and D) initial condition.

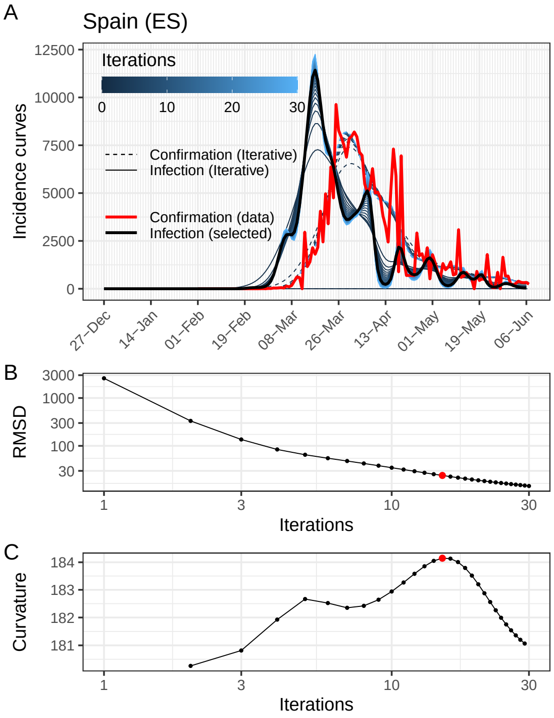

(A) **Deconvolution iterations using uninformative initial condition.** A. The RL deconvolution iterative curves and observed confirmation curve. B: The root-mean-square difference (RMSD) of all iterations. C: The curvature of RMSD. The red dot represents the selected iteration with maximum curvature.

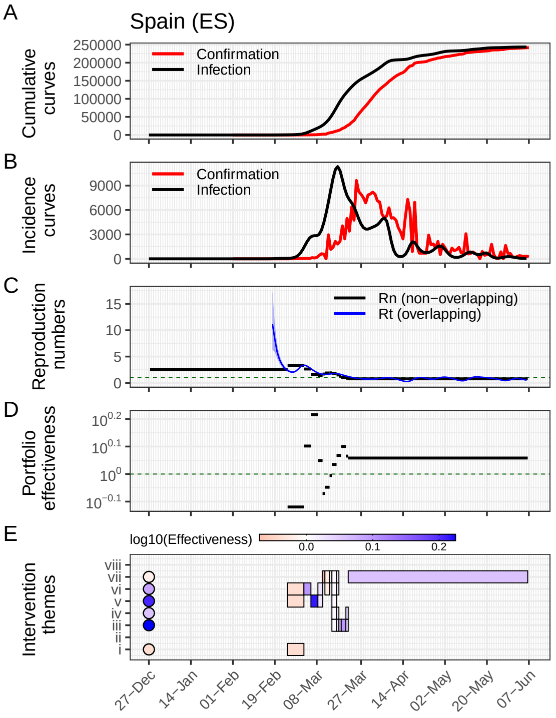

(B) **Estimates using uninformative initial condition.** A. The cumulative curves. B. The incidence curves. C. The instantaneous reproduction numbers R_n and R_t. D. The portfolio effectiveness η_n. E. The intervention $\eta_n^{(m)}$ (in rectangles) and aggregated $\eta^{(m)}$ (in circles) effectiveness.

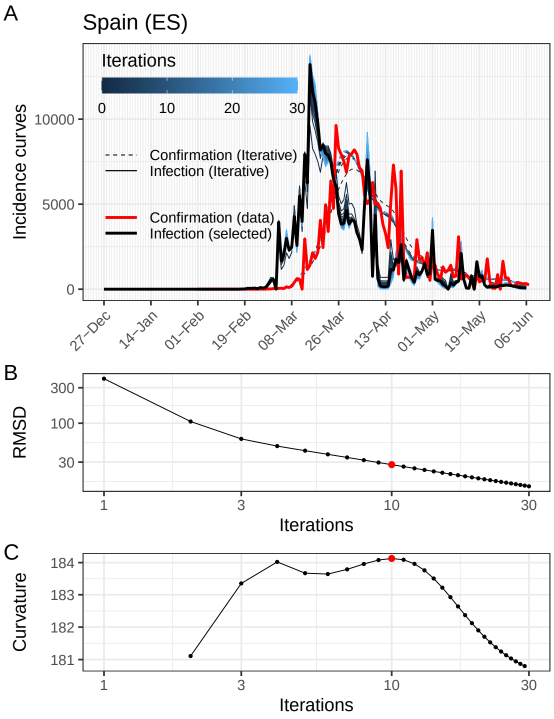

(C) **Deconvolution iterations using informative initial condition.** A. The RL deconvolution iterative curves and observed confirmation curve. B: The root-mean-square difference (RMSD) of all iterations. C: The curvature of RMSD. The red dot represents the selected iteration with maximum curvature.

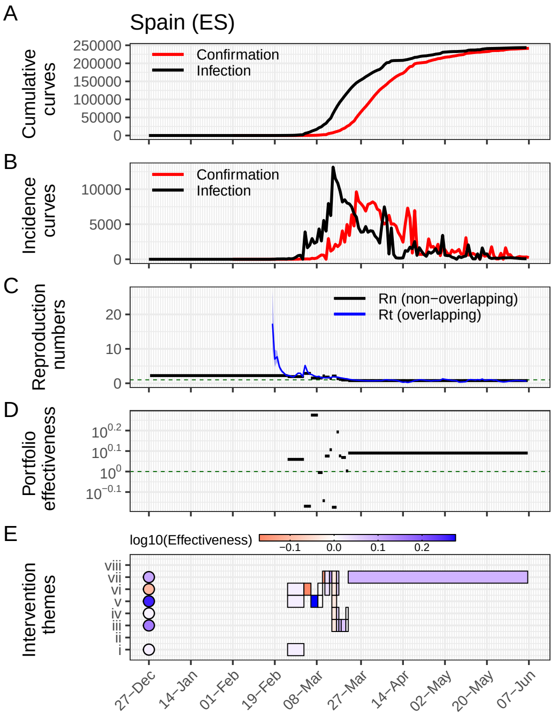

(D) **Estimates using informative initial condition.** A. The cumulative curves. B. The incidence curves. C. The instantaneous reproduction numbers R_n and R_t. D. The portfolio effectiveness η_n. E. The intervention $\eta_n^{(m)}$ (in rectangles) and aggregated $\eta^{(m)}$ (in circles) effectiveness.

Figure S44. **The reconstructed epidemic curves and subsequent evaluation of interventions implemented in Spain.** Estimation using uninformative (baseline setting, A and B) and informative (alternative setting, C and D) initial condition.

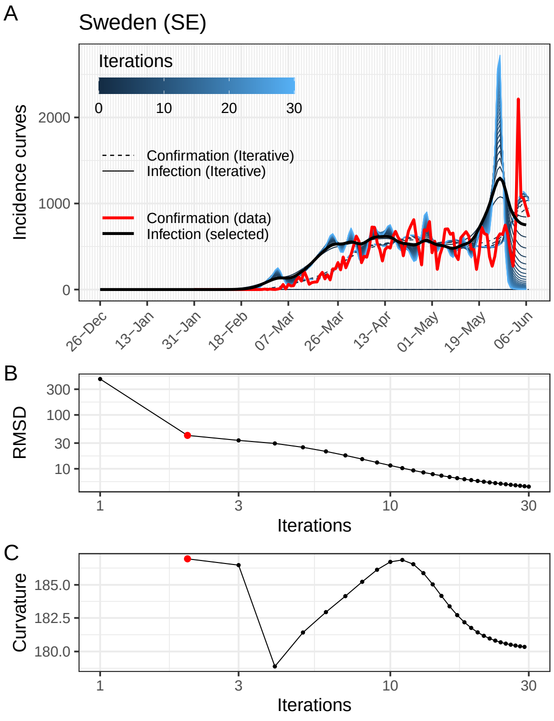

(A) **Deconvolution iterations using uninformative initial condition.** A. The RL deconvolution iterative curves and observed confirmation curve. B: The root-mean-square difference (RMSD) of all iterations. C: The curvature of RMSD. The red dot represents the selected iteration with maximum curvature.

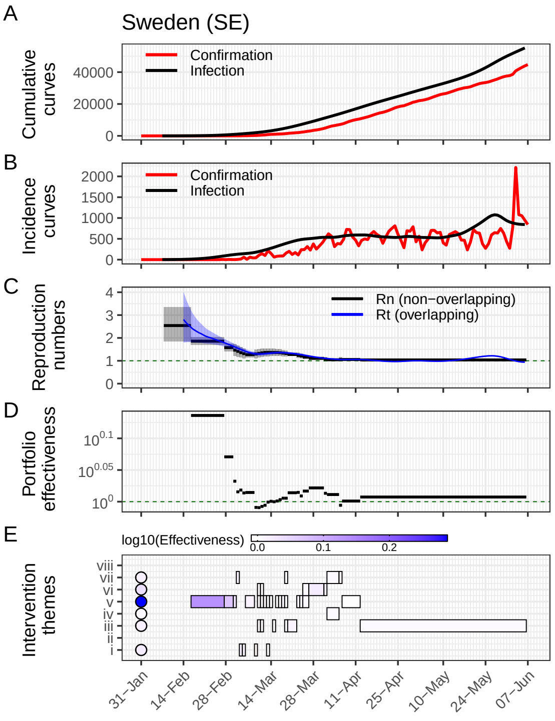

(B) **Estimates using uninformative initial condition.** A. The cumulative curves. B. The incidence curves. C. The instantaneous reproduction numbers R_n and R_t. D. The portfolio effectiveness η_n. E. The intervention $\eta_n^{(m)}$ (in rectangles) and aggregated $\eta^{(m)}$ (in circles) effectiveness.

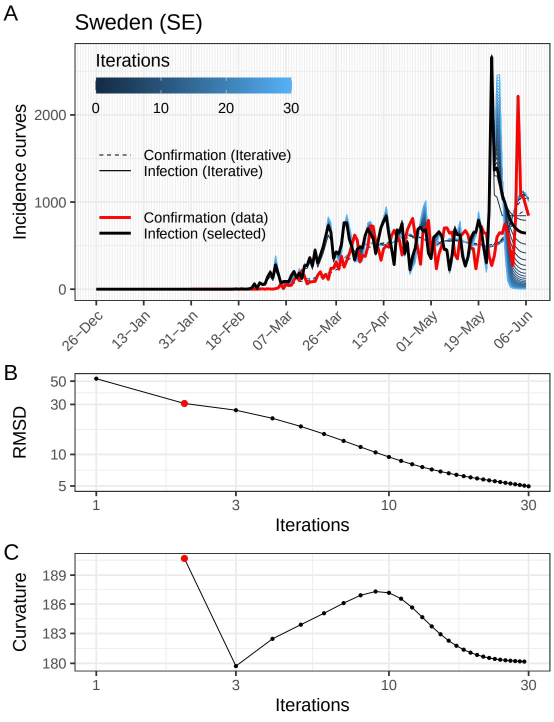

(C) **Deconvolution iterations using informative initial condition.** A. The RL deconvolution iterative curves and observed confirmation curve. B: The root-mean-square difference (RMSD) of all iterations. C: The curvature of RMSD. The red dot represents the selected iteration with maximum curvature.

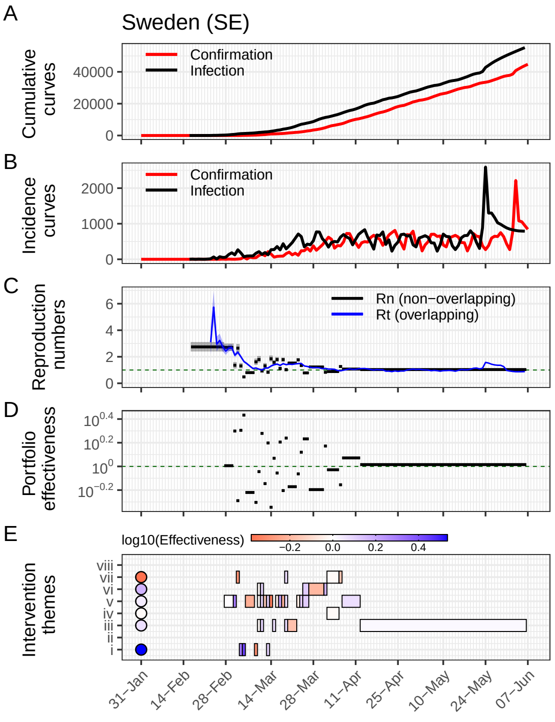

(D) **Estimates using informative initial condition.** A. The cumulative curves. B. The incidence curves. C. The instantaneous reproduction numbers R_n and R_t. D. The portfolio effectiveness η_n. E. The intervention $\eta_n^{(m)}$ (in rectangles) and aggregated $\eta^{(m)}$ (in circles) effectiveness.

FIGURE S45. **The reconstructed epidemic curves and subsequent evaluation of interventions implemented in Sweden.** Estimation using uninformative (baseline setting, A and B) and informative (alternative setting, C and D) initial condition.

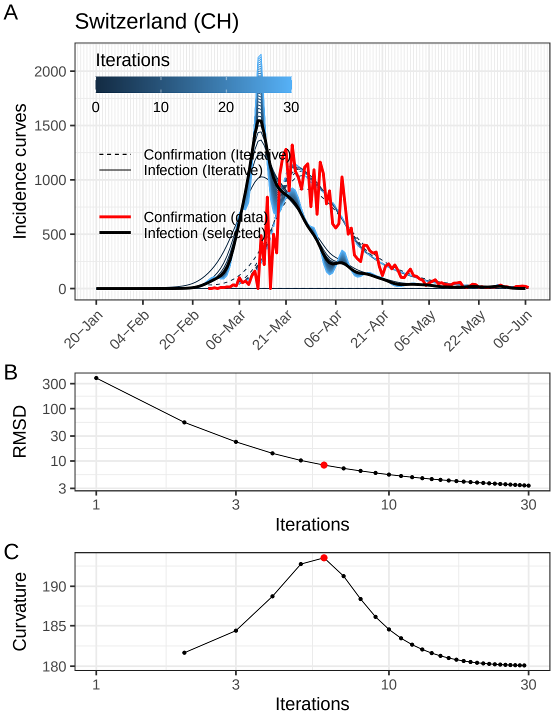

(A) **Deconvolution iterations using uninformative initial condition.** A. The RL deconvolution iterative curves and observed confirmation curve. B: The root-mean-square difference (RMSD) of all iterations. C: The curvature of RMSD. The red dot represents the selected iteration with maximum curvature.

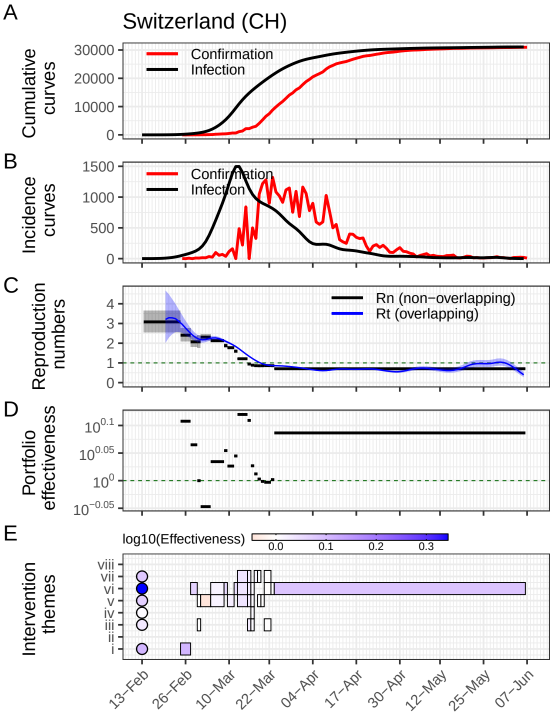

(B) **Estimates using uninformative initial condition.** A. The cumulative curves. B. The incidence curves. C. The instantaneous reproduction numbers R_n and R_t. D. The portfolio effectiveness η_n. E. The intervention $\eta_n^{(m)}$ (in rectangles) and aggregated $\eta^{(m)}$ (in circles) effectiveness.

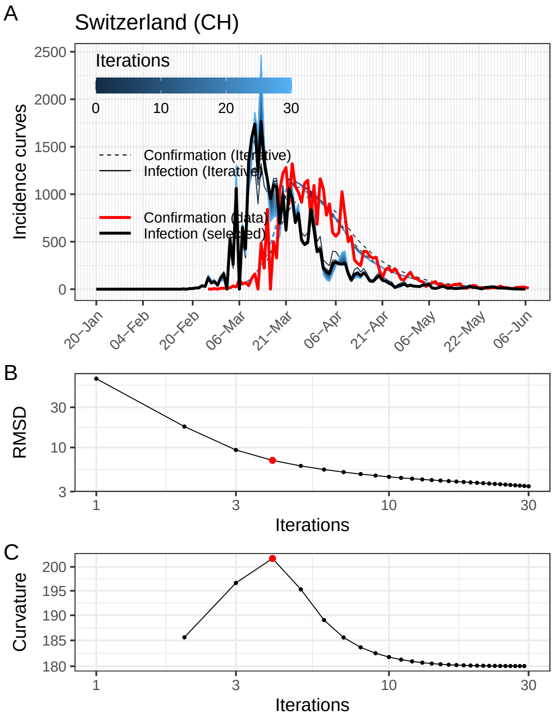

(C) **Deconvolution iterations using informative initial condition.** A. The RL deconvolution iterative curves and observed confirmation curve. B: The root-mean-square difference (RMSD) of all iterations. C: The curvature of RMSD. The red dot represents the selected iteration with maximum curvature.

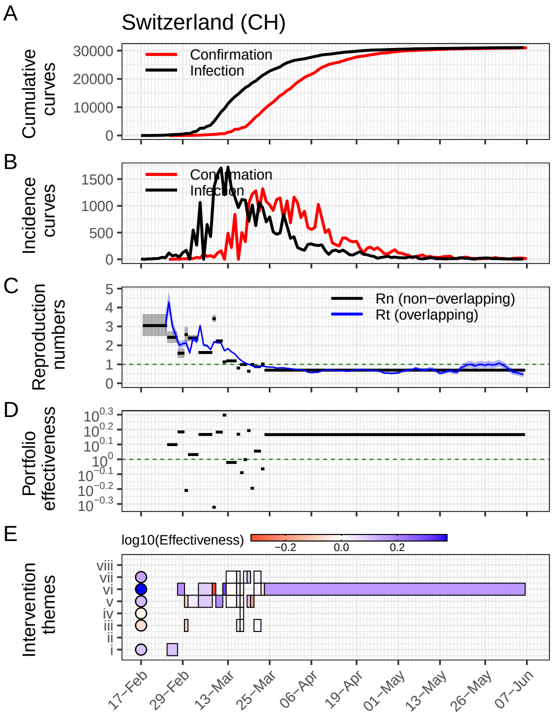

(D) **Estimates using informative initial condition.** A. The cumulative curves. B. The incidence curves. C. The instantaneous reproduction numbers R_n and R_t. D. The portfolio effectiveness η_n. E. The intervention $\eta_n^{(m)}$ (in rectangles) and aggregated $\eta^{(m)}$ (in circles) effectiveness.

FIGURE S46. **The reconstructed epidemic curves and subsequent evaluation of interventions implemented in Switzerland.** Estimation using uninformative (baseline setting, A and B) and informative (alternative setting, C and D) initial condition.

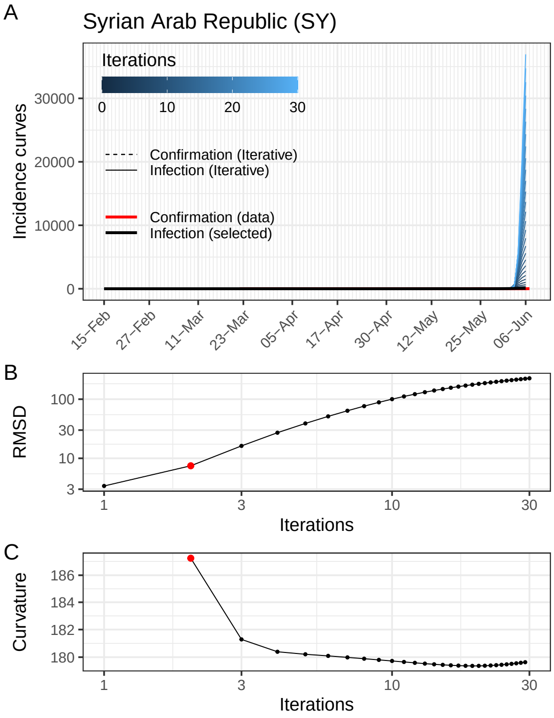

(A) **Deconvolution iterations using uninformative initial condition.** A. The RL deconvolution iterative curves and observed confirmation curve. B: The root-mean-square difference (RMSD) of all iterations. C: The curvature of RMSD. The red dot represents the selected iteration with maximum curvature.

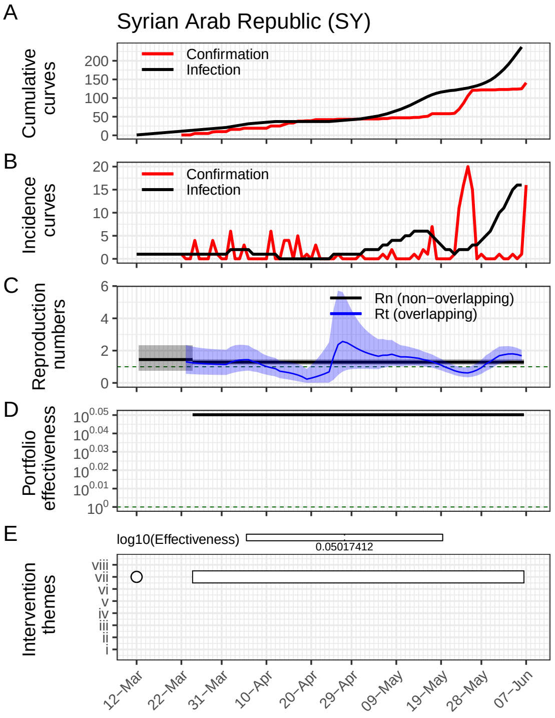

(B) **Estimates using uninformative initial condition.** A. The cumulative curves. B. The incidence curves. C. The instantaneous reproduction numbers R_n and R_t. D. The portfolio effectiveness η_n. E. The intervention $\eta_n^{(m)}$ (in rectangles) and aggregated $\eta^{(m)}$ (in circles) effectiveness.

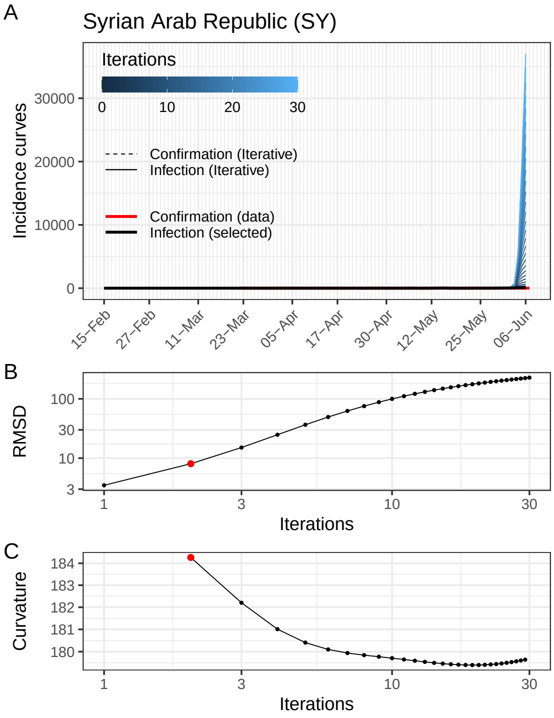

(C) **Deconvolution iterations using informative initial condition.** A. The RL deconvolution iterative curves and observed confirmation curve. B: The root-mean-square difference (RMSD) of all iterations. C: The curvature of RMSD. The red dot represents the selected iteration with maximum curvature.

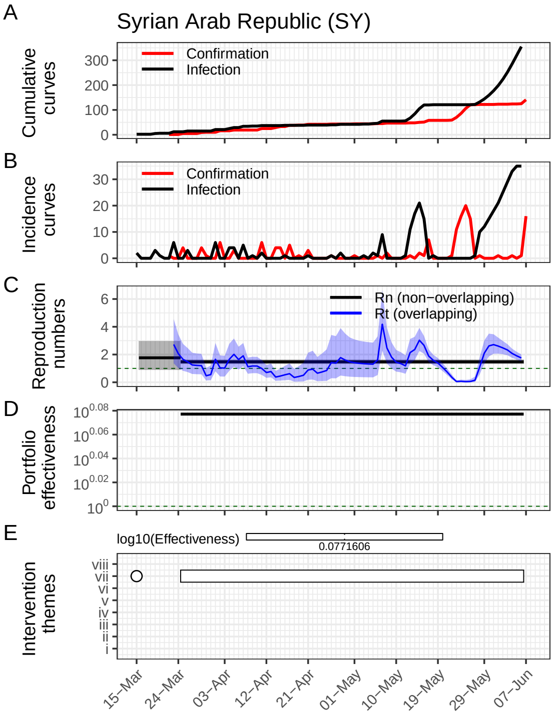

(D) **Estimates using informative initial condition.** A. The cumulative curves. B. The incidence curves. C. The instantaneous reproduction numbers R_n and R_t. D. The portfolio effectiveness η_n. E. The intervention $\eta_n^{(m)}$ (in rectangles) and aggregated $\eta^{(m)}$ (in circles) effectiveness.

FIGURE S47. **The reconstructed epidemic curves and subsequent evaluation of interventions implemented in Syrian Arab Republic.** Estimation using uninformative (baseline setting, A and B) and informative (alternative setting, C and D) initial condition.

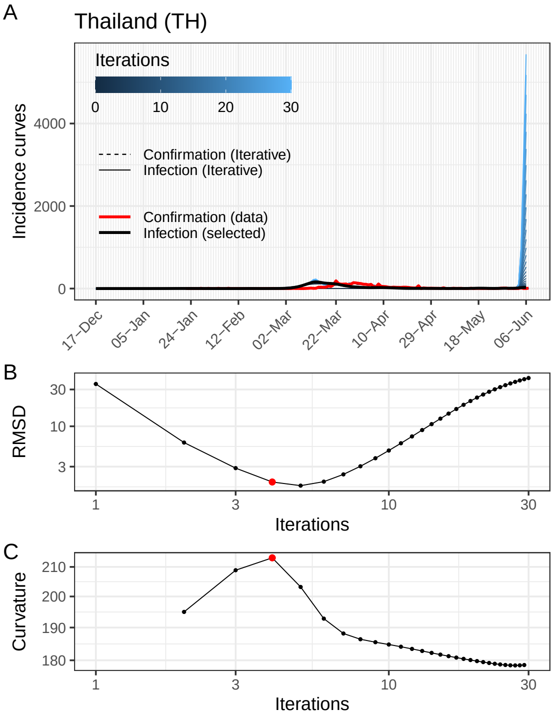

(A) **Deconvolution iterations using uninformative initial condition.** A. The RL deconvolution iterative curves and observed confirmation curve. B: The root-mean-square difference (RMSD) of all iterations. C: The curvature of RMSD. The red dot represents the selected iteration with maximum curvature.

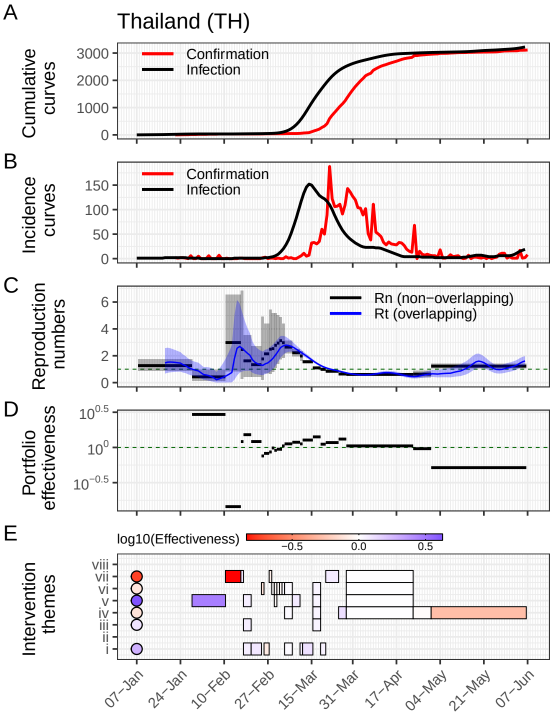

(B) **Estimates using uninformative initial condition.** A. The cumulative curves. B. The incidence curves. C. The instantaneous reproduction numbers R_n and R_t. D. The portfolio effectiveness η_n. E. The intervention $\eta_n^{(m)}$ (in rectangles) and aggregated $\eta^{(m)}$ (in circles) effectiveness.

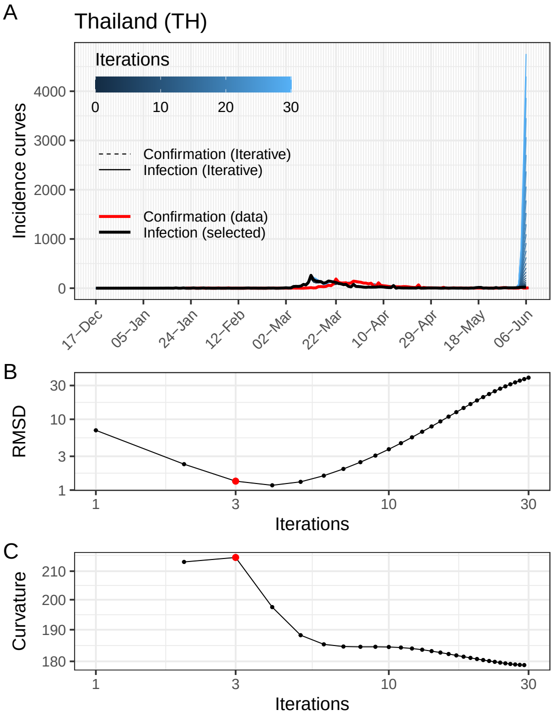

(C) **Deconvolution iterations using informative initial condition.** A. The RL deconvolution iterative curves and observed confirmation curve. B: The root-mean-square difference (RMSD) of all iterations. C: The curvature of RMSD. The red dot represents the selected iteration with maximum curvature.

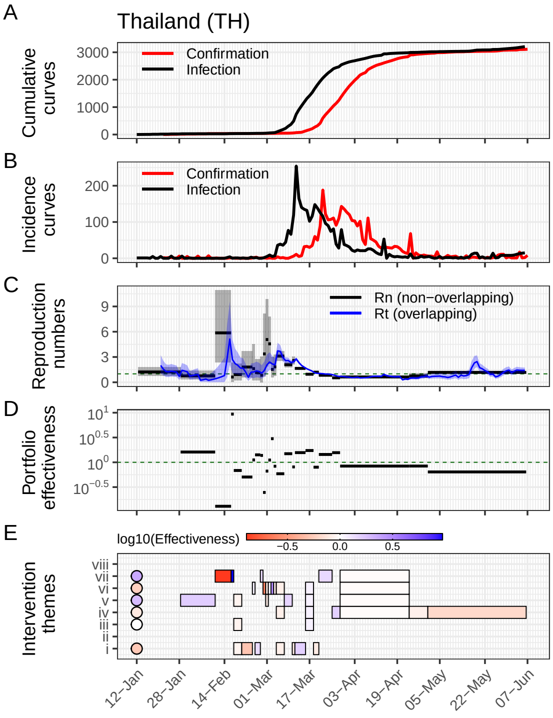

(D) **Estimates using informative initial condition.** A. The cumulative curves. B. The incidence curves. C. The instantaneous reproduction numbers R_n and R_t. D. The portfolio effectiveness η_n. E. The intervention $\eta_n^{(m)}$ (in rectangles) and aggregated $\eta^{(m)}$ (in circles) effectiveness.

FIGURE S48. **The reconstructed epidemic curves and subsequent evaluation of interventions implemented in Thailand.** Estimation using uninformative (baseline setting, A and B) and informative (alternative setting, C and D) initial condition.

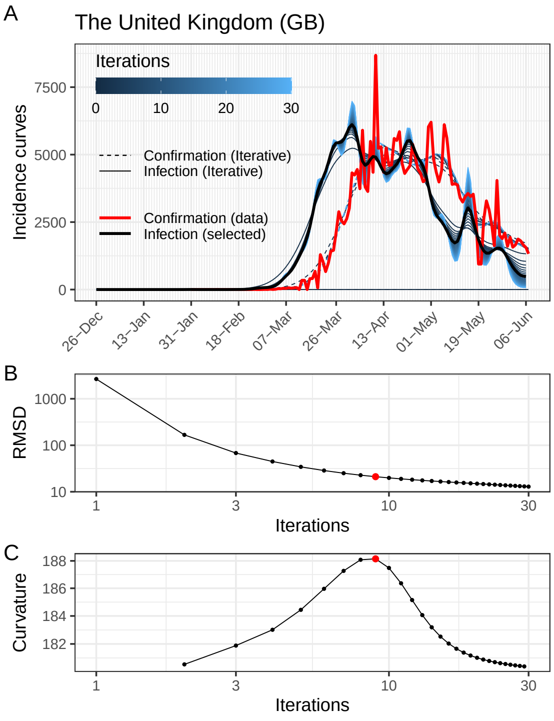

(A) **Deconvolution iterations using uninformative initial condition.** A. The RL deconvolution iterative curves and observed confirmation curve. B: The root-mean-square difference (RMSD) of all iterations. C: The curvature of RMSD. The red dot represents the selected iteration with maximum curvature.

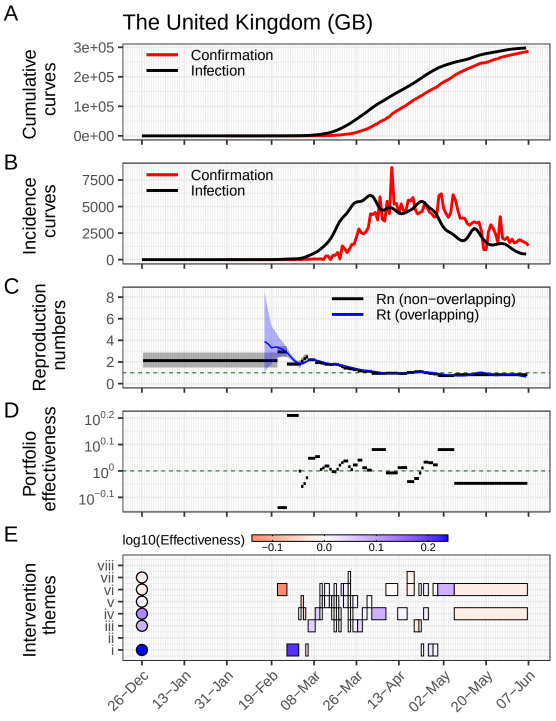

(B) **Estimates using uninformative initial condition.** A. The cumulative curves. B. The incidence curves. C. The instantaneous reproduction numbers R_n and R_t. D. The portfolio effectiveness η_n. E. The intervention $\eta_n^{(m)}$ (in rectangles) and aggregated $\eta^{(m)}$ (in circles) effectiveness.

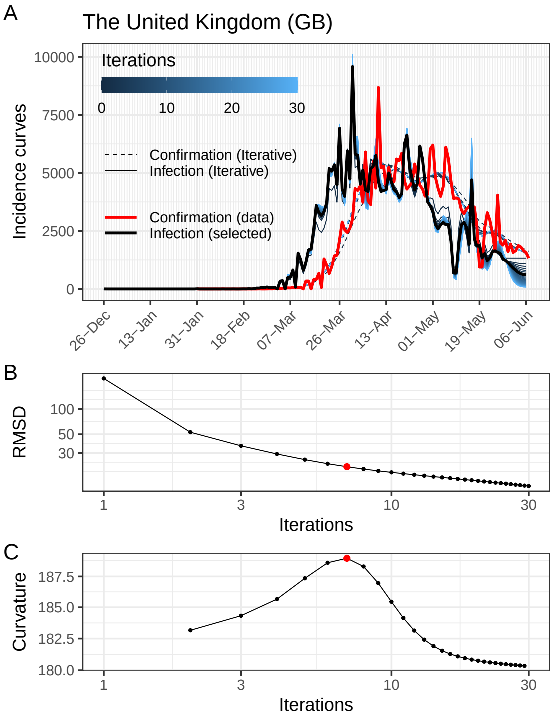

(C) **Deconvolution iterations using informative initial condition.** A. The RL deconvolution iterative curves and observed confirmation curve. B: The root-mean-square difference (RMSD) of all iterations. C: The curvature of RMSD. The red dot represents the selected iteration with maximum curvature.

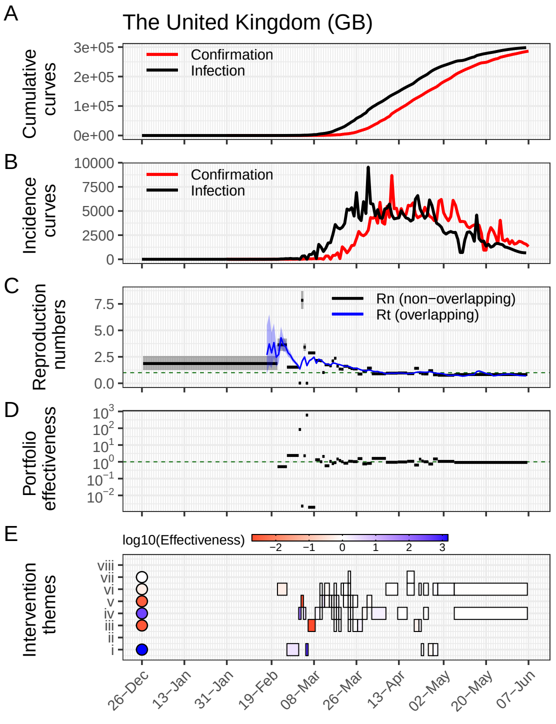

(D) **Estimates using informative initial condition.** A. The cumulative curves. B. The incidence curves. C. The instantaneous reproduction numbers R_n and R_t. D. The portfolio effectiveness η_n. E. The intervention $\eta_n^{(m)}$ (in rectangles) and aggregated $\eta^{(m)}$ (in circles) effectiveness.

FIGURE S49. **The reconstructed epidemic curves and subsequent evaluation of interventions implemented in The United Kingdom.** Estimation using uninformative (baseline setting, A and B) and informative (alternative setting, C and D) initial condition.

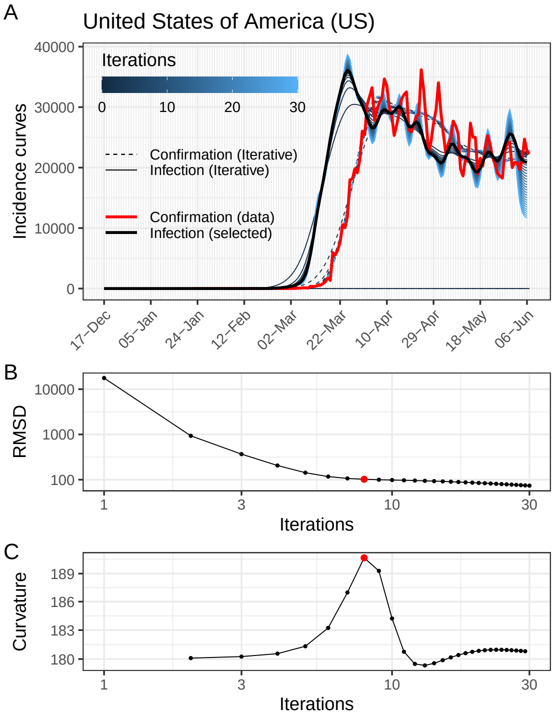

(A) **Deconvolution iterations using uninformative initial condition.** A. The RL deconvolution iterative curves and observed confirmation curve. B: The root-mean-square difference (RMSD) of all iterations. C: The curvature of RMSD. The red dot represents the selected iteration with maximum curvature.

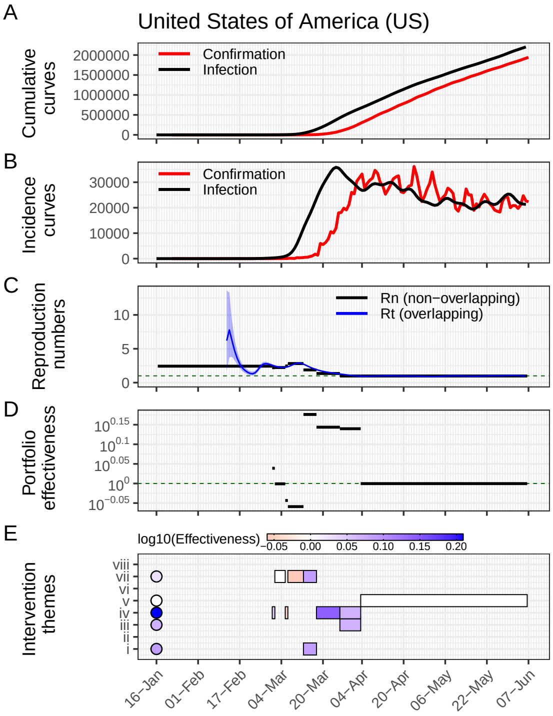

(B) **Estimates using uninformative initial condition.** A. The cumulative curves. B. The incidence curves. C. The instantaneous reproduction numbers R_n and R_t. D. The portfolio effectiveness η_n. E. The intervention $\eta_n^{(m)}$ (in rectangles) and aggregated $\eta^{(m)}$ (in circles) effectiveness.

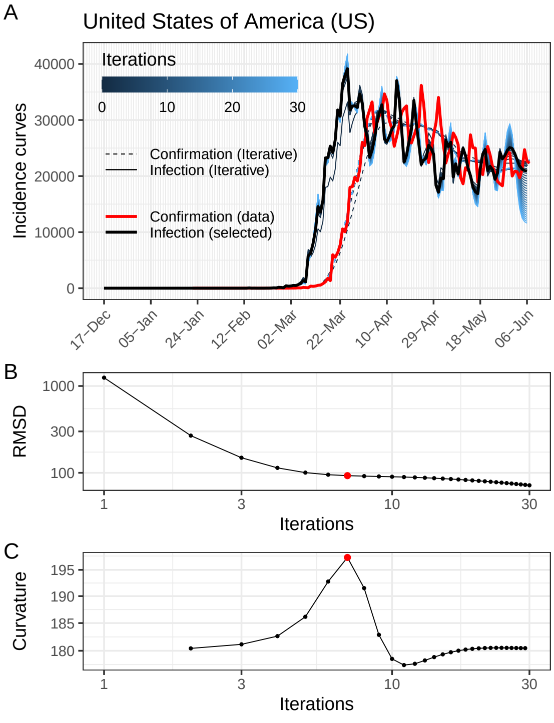

(C) **Deconvolution iterations using informative initial condition.** A. The RL deconvolution iterative curves and observed confirmation curve. B: The root-mean-square difference (RMSD) of all iterations. C: The curvature of RMSD. The red dot represents the selected iteration with maximum curvature.

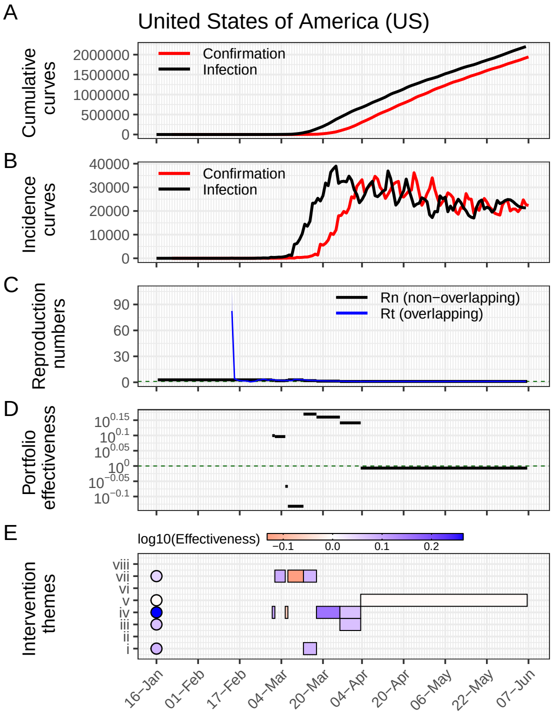

(D) **Estimates using informative initial condition.** A. The cumulative curves. B. The incidence curves. C. The instantaneous reproduction numbers R_n and R_t. D. The portfolio effectiveness η_n. E. The intervention $\eta_n^{(m)}$ (in rectangles) and aggregated $\eta^{(m)}$ (in circles) effectiveness.

FIGURE S50. **The reconstructed epidemic curves and subsequent evaluation of interventions implemented in United States of America.** Estimation using uninformative (baseline setting, A and B) and informative (alternative setting, C and D) initial condition.

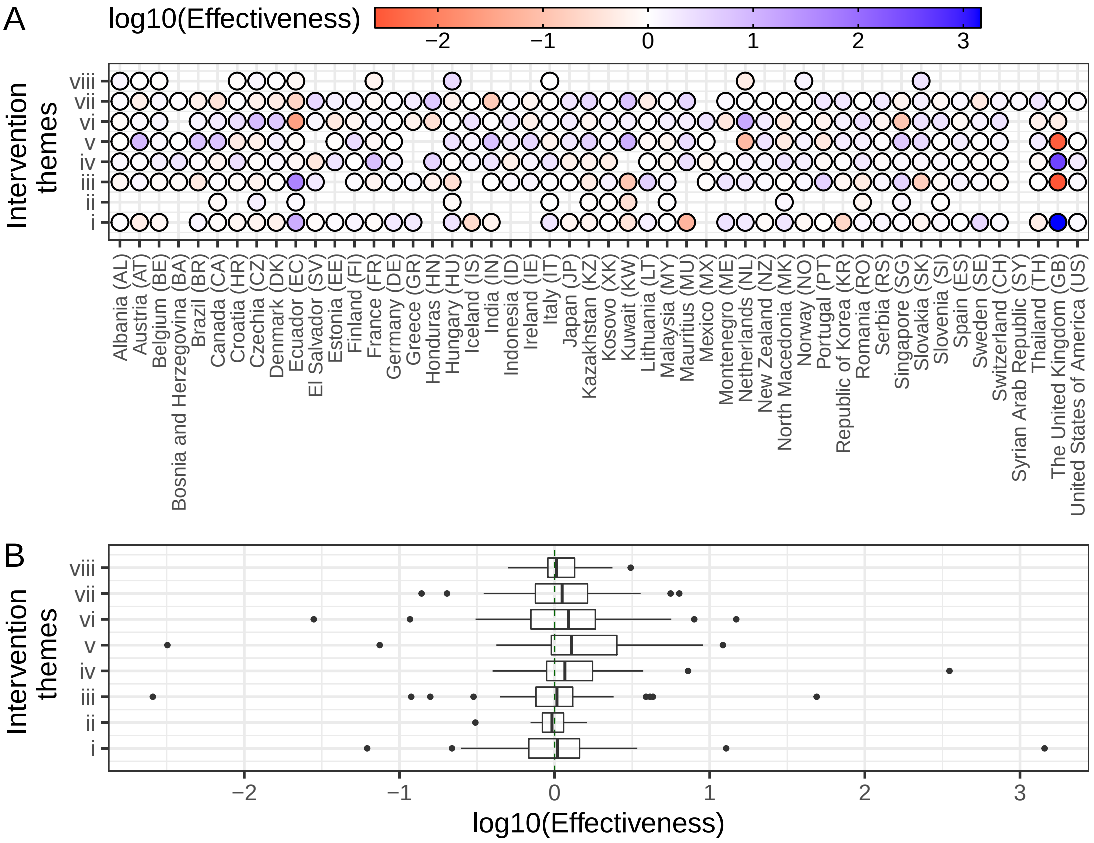

Figure S51. **Aggregated portfolio effectiveness of intervention themes implemented in 50 selected countries using informative initial condition (alternative setting).** The eight themes are *(i) Case identification, contact tracing and related measures*, *(ii) Environmental measures*, *(iii) Healthcare and public health capacity*, *(iv) Resource allocation*, *(v) Risk communication*, *(vi) Social distancing*, *(vii) Travel restriction*, and *(viii) Returning to normal life*. A. The blue (or red) color represents the interventions that successfully reduced infections (or increment of infections). B. The boxes shows the distributions of the aggregated effectiveness in log10-scale. The median values of the eight themes were 0.02, -0.02, 0.01, 0.07, 0.16, 0.10, 0.04, and 0.01, respectively.

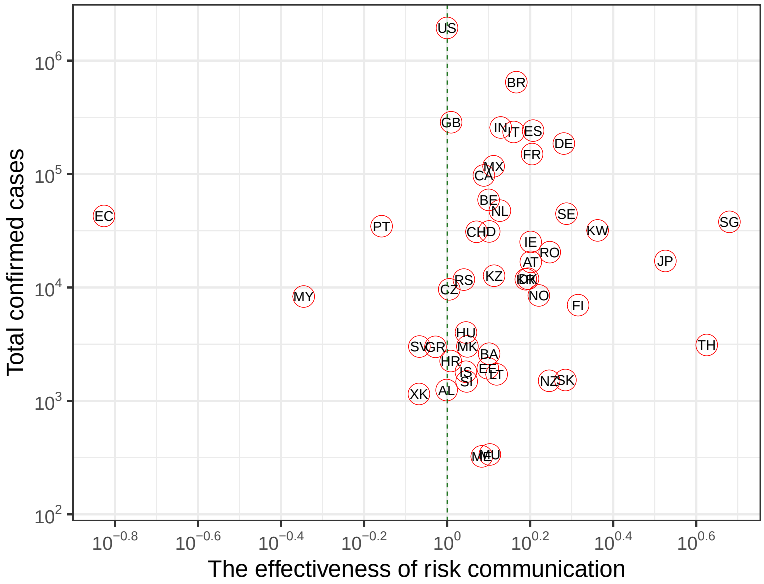

FIGURE S52. **Total confirmed cases as a function of the effectiveness of risk communication.** 48 countries (implemented risk communication intervention) are shown in red circles with their ISO codes. The vertical dashed green line represents the criticality of effectiveness. Note that the total confirmed cases on y-axis is shown in log10-scale.

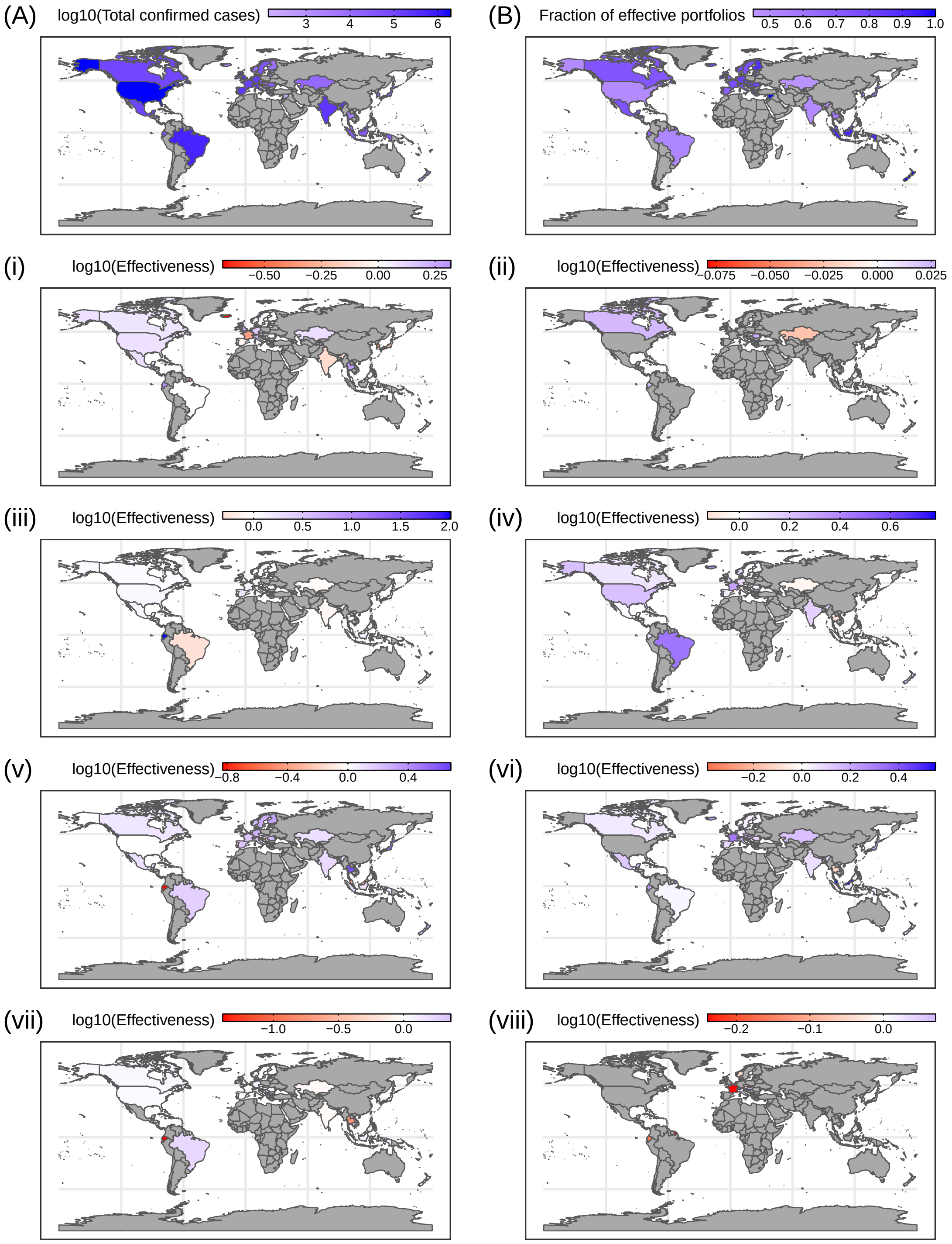

FIGURE S53. **Maps of 50 countries.** (A) The total confirmed cases. (B) The fraction of effective portfolios. (i-viii) The aggregated portfolio effectiveness of eight themes: *(i) Case identification, contact tracing and related measures*, *(ii) Environmental measures*, *(iii) Healthcare and public health capacity*, *(iv) Resource allocation*, *(v) Risk communication*, *(vi) Social distancing*, *(vii) Travel restriction*, and *(viii) Returning to normal life*.
